# Exploration of non-hydroxamate based quinoline analogues as HDAC8 inhibitors

**DOI:** 10.1039/d6ra06186a

**Published:** 2026-08-18

**Authors:** N. V. M. Rao Bandaru, Vandana Joshi, Markus Schweipert, Kosana Sai Chaitanya, Dharani Sen, Muzaffar-Ur-Rehman Mohammed, Vivek Sharma, Chandrasekhar Abbineni, Franz-Josef Meyer-Almes, Kondapalli Venkata Gowri Chandra Sekhar

**Affiliations:** a Department of Chemistry, Birla Institute of Technology and Science, Pilani, Hyderabad Campus Jawahar Nagar Hyderabad 500078 Telangana India kvgc@hyderabad.bits-pilani.ac.in; b Department of Biological Sciences, Birla Institute of Technology and Science, Pilani, Hyderabad Campus Jawahar Nagar Hyderabad 500078 Telangana India; c Aurigene Oncology Limited 39-40 KIADB Industrial Area Electronic City Phase II, Hosur Road Bangalore 560100 India; d Department of Chemical Engineering and Biotechnology, University of Applied Sciences Darmstadt Haardtring 100 64295 Darmstadt Germany franz-josef.meyer-almes@h-da.de; e European University of Technology European Union; f Department of Pharmacy, Birla Institute of Technology and Science, Pilani Campus Pilani 333031 Rajasthan India

## Abstract

Histone deacetylase 8 (HDAC8) has emerged as a promising therapeutic target due to its role in epigenetic regulation. In this study, novel non-hydroxamate-based quinoline analogues were designed and explored as HDAC8-selective inhibitors that exhibit nanomolar HDAC8 enzymatic inhibition. Notably, compounds BHC-10 and BHC-13 showed 30 and 42 nM activity, respectively. Compounds exhibiting below 500 nM activity were evaluated for their *in vitro* cellular activity, and two compounds, BHC-2 and BHC-10, showed promising activity in neuroblastoma cell lines (IMR-32 and SH-SY5Y). Based on both enzymatic and cellular assays, compounds BHC-2, BHC-10, and BHC-13 were selected for further evaluation using clonogenic growth assays, apoptosis assays, and cell cycle analysis. Clonogenic assays revealed a significant, dose-dependent reduction in colony formation. Flow cytometry analysis indicated a dose-dependent increase in apoptotic cell populations and induction of cell cycle arrest at the S phase. Mechanistic studies confirmed target engagement through enhanced acetylation of SMC3, as demonstrated by western blot analysis. Additionally, molecular docking analysis provided insights into the binding interactions of these compounds with the HDAC8 protein. Two compounds from the current work, BHC-10 and BHC-13, emerged as more potent and selective than the reference compound B. Together, the current findings establish these compounds as promising HDAC8 modulators with potential for therapeutic development in neuroblastoma.

## Abstract 2

Histone deacetylase 8 (HDAC8) has emerged as a promising therapeutic target due to its role in epigenetic regulation.

## Graphical abstract

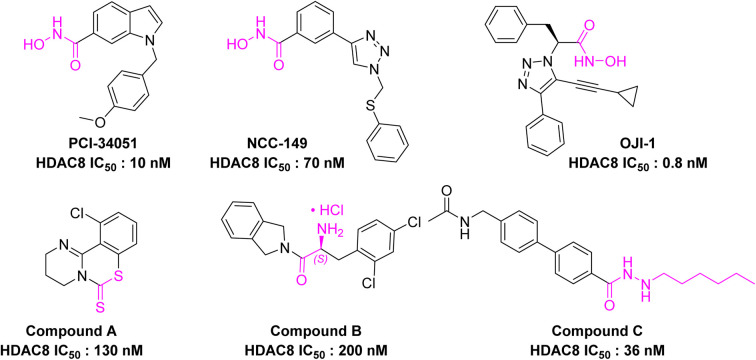

## 1. Introduction

The histone deacetylase (HDAC) family of enzymes plays a crucial role in regulating gene expression by catalysing the removal of acetyl groups from histone and non-histone proteins.^1^ This post-translational modification is a key epigenetic mechanism that modulates chromatin structure and transcription, impacting fundamental cellular processes such as proliferation, differentiation, and apoptosis.^2,3^ Dysregulation of HDACs has been implicated in the pathophysiology of numerous diseases, including various cancers, neurodegenerative disorders, inflammatory disorders, and infectious diseases.^4–6^ As a result, HDACs have become a significant class of drug targets, with pan-HDAC inhibitors already demonstrating clinical utility in the treatment of certain haematological malignancies.^7–15^ However, while pan-HDAC inhibitors show therapeutic promise, their broad-spectrum activity often causes dose-limiting toxicities that hinder their widespread application.^16^ This limitation has prompted a shift towards developing isoform-selective inhibitors to enhance efficacy and reduce off-target effects. Among the 18 human HDAC isoforms, HDAC8 stands out as a compelling target. As a member of the class I HDACs, HDAC8 is a metalloenzyme requiring a zinc ion for catalytic activity.^17,18^ It is ubiquitously expressed in various tissues and involved in diverse cellular functions, including smooth muscle contraction, T-cell activation, and the regulation of gene expression in neural development. In oncology, HDAC8 is overexpressed in several cancers, including neuroblastoma, breast cancer, and colorectal cancer, where it promotes tumour cell survival and proliferation.^19–22^ Beyond oncology, its dysregulation has been implicated in a rare genetic disorder known as Cornelia de Lange Syndrome (CdLS), where mutations in the NIPBL gene lead to altered ohesion complex function, and HDAC8-mediated deacetylation of the ohesion subunit has been implicated in the disease phenotype.^23^

**Fig. 1:**
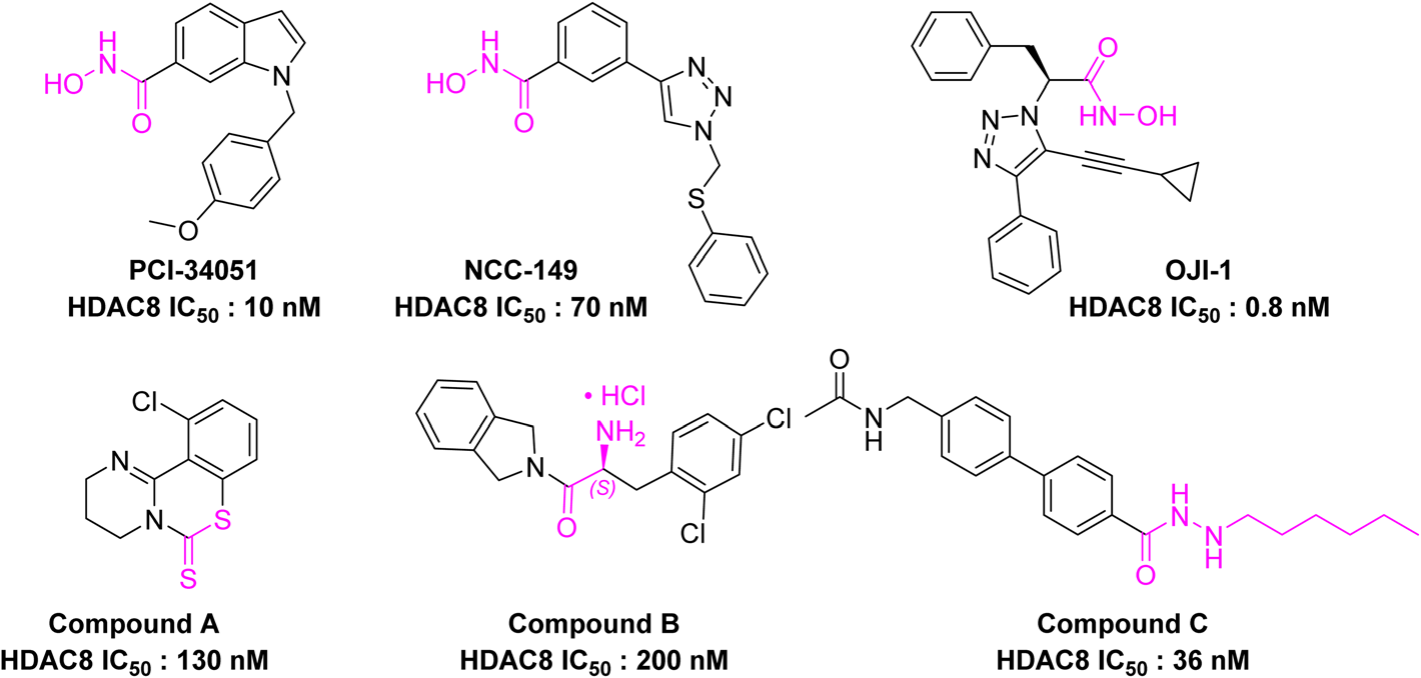
Representation of selective HDAC8 inhibitors.

Most potent HDAC inhibitors share a canonical pharmacophore comprising a zinc-binding group (ZBG), a linker, and a cap group.^24,25^ Early HDAC8 inhibitors relied on hydroxamate groups to achieve strong activity; these compounds have shown shortcomings, including suboptimal metabolic stability, risk of adverse effects, and limited selectivity among HDAC isoforms.^26,27^ These challenges have sparked considerable interest in the discovery and development of non-hydroxamate-based HDAC8 inhibitors. Non-hydroxamate alternatives, including α-amino amides, benzamides, *o*-aminoanilides, hydrazides, carboxylic acids, electrophilic ketones, thiols, and mercaptoamides, offer unique and often improved pharmacological properties.^28–32^ Notably, representative selective HDAC8 inhibitors are shown in Fig. 1. PCI-34051, NCC-149, and OJI-1 are well-established hydroxamate-based HDAC8 inhibitors.^33–35^ In recent years, besides hydroxymate-based HDAC8 inhibitors, non-hydroxymate-based inhibitors have been reported as selective HDAC8 inhibitors. Compound A, a benzothiazinethione derivative, showed good selectivity for HDAC8, with an IC_50_ of 0.13 µM.^36^ Whitehead *et al.* reported that the amino acid derivative B displayed good selectivity for HDAC8, with an IC_50_ of 0.20 µM.^36–38^ Alkyl hydrazide is used as a selective HDAC8 inhibitor by the Ping Sun group.^39^

## 2. Rational drug design

**Fig. 2:**
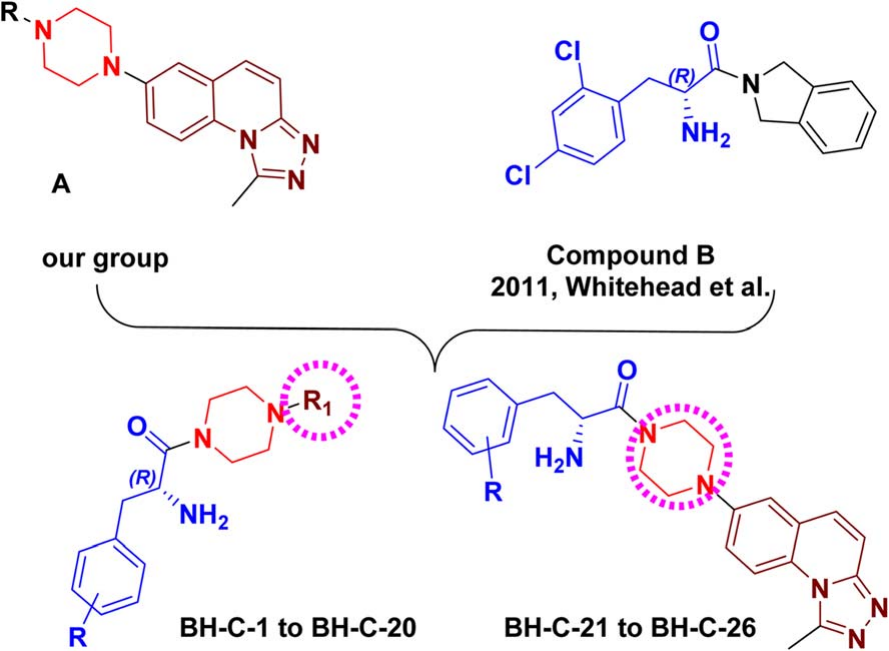
Design strategy for BHC- (quinoline derivatives) series.

Non-hydroximate HDAC8 inhibitors have taken priority over hydroximate-based HDAC8 inhibitors, as the latter are associated with adverse pharmacokinetics and putative mutagenicity. For instance, the whitehead group developed α-amino amides as a Zn-binding group, and compound B showed promising activity against HDAC8. Based on compound B, we previously reported a series of alpha-amino amides as HDAC8 inhibitors by varying the different cap groups, showing that the triazoloquinoline cap group with smaller hydrophobic groups, such as methyl, trifluoromethyl, and cyclopropyl, turned out to be potent compounds.^40^ From the docking analysis of our earlier reported compounds, we observed that the alpha amino amide and chloro substituted phenyl rings showed several interactions with the active site residue such as Gly140, Trp141, His142 and His143 while the 1,2,4-triazolo[4,3-*a*] quinoline ring had shown least interactions such as π-stacking interactions with Phe152.^40^ By this, it was clear that there was ample scope to improve the HDAC8 inhibitory activity of these compounds by catching the additional interactions by the quinoline moiety. To capture the additional surface interactions, we designed a series of quinoline derivatives, BHC-1 to BHC-20, by varying the nitrogen position in quinoline and by providing flexibility in the triazolo quinoline core with various substituents on quinoline in the surface region of the HDAC8 protein. We designed an additional set of compounds, BHC-21 to BHC-26, by varying the substituent on piperazine to occupy the pocket, as shown in Fig. 2.

## 3. Synthesis of designed compounds

**Scheme 1:**
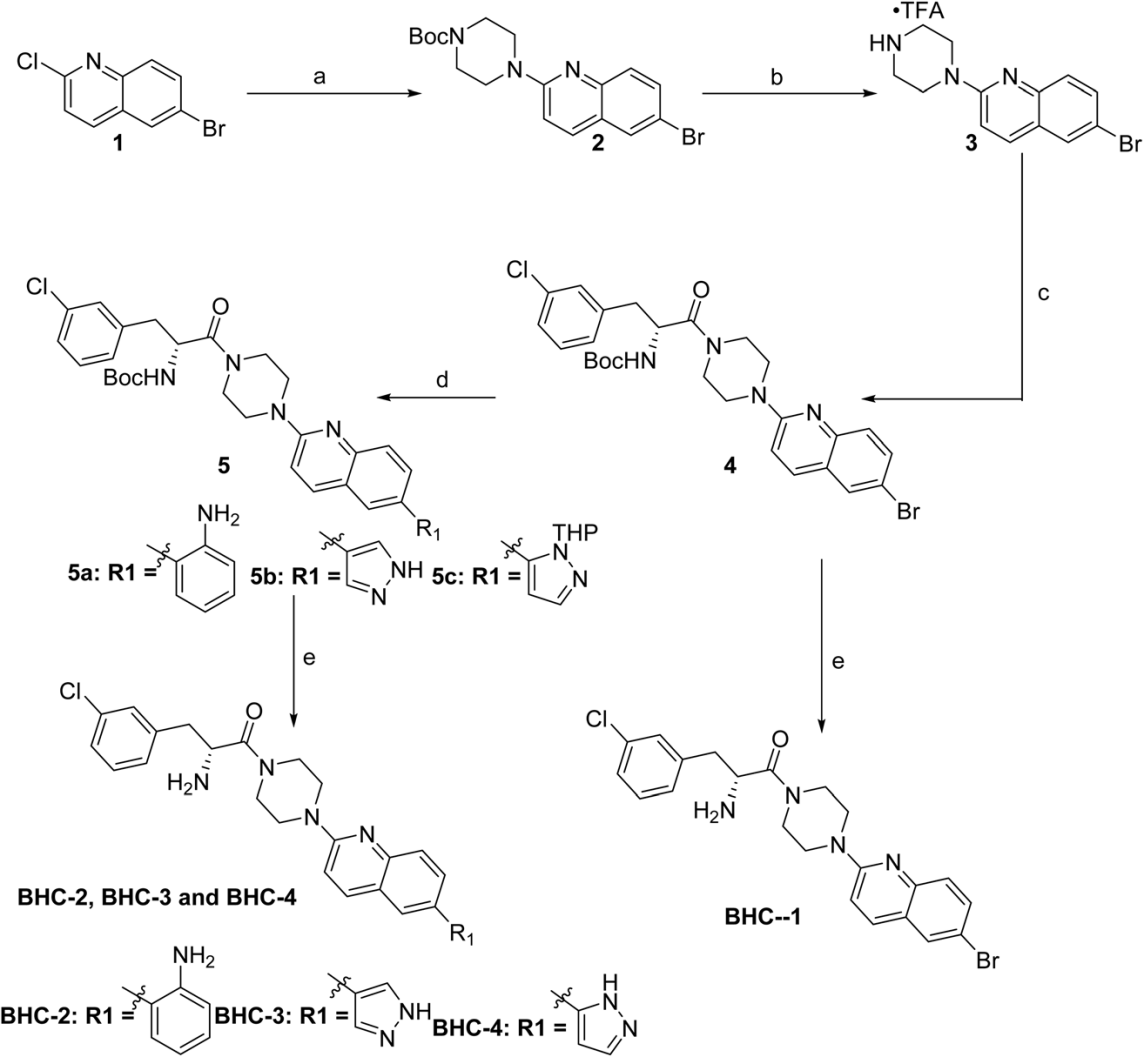
The synthetic route for compounds BHC-1 to BHC-4. Reagents and conditions: (a) *tert*-butyl piperazine-1-carboxylate, Cs_2_CO_3_, DMF, 90 °C, 16 h; (b) TFA, DCM, RT, 4 h; (c) HATU, DIPEA, DMF, RT, 2 h; (d) corresponding boronic acid or boronic ester, Cs_2_CO_3_, Pd(dppf)Cl_2_. DCM, 1,4-dioxane, H_2_O, 90 °C, 16 h; (e) 4N HCl in 1,4-dioxane, RT, 2 h.

**Scheme 2:**
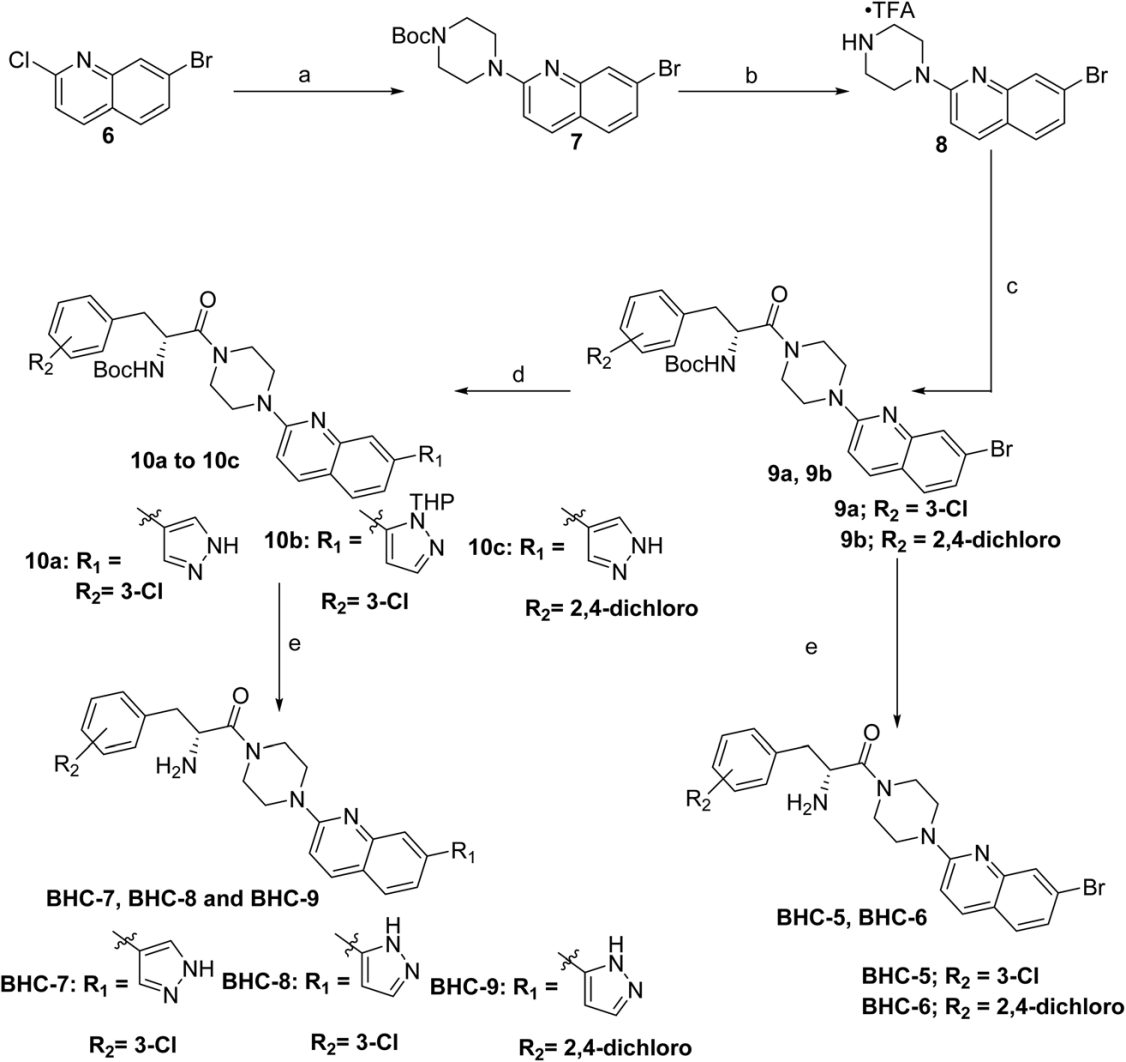
The synthetic route for compounds BHC-5 to BHC-9. Reagents and conditions: (a) *tert*-butyl piperazine-1-carboxylate, Cs_2_CO_3_, DMF, 90 °C, 16 h; (b) TFA, DCM, RT, 4 h; (c) HATU, DIPEA, DMF, RT, 2 h; (d) corresponding boronic acid or boronic ester, Cs_2_CO_3_, Pd(dppf)Cl_2_. DCM, 1,4-dioxane, H_2_O, 90 °C, 16 h; (e) 4N HCl in 1,4-dioxane, RT, 2 h.

**Scheme 3:**
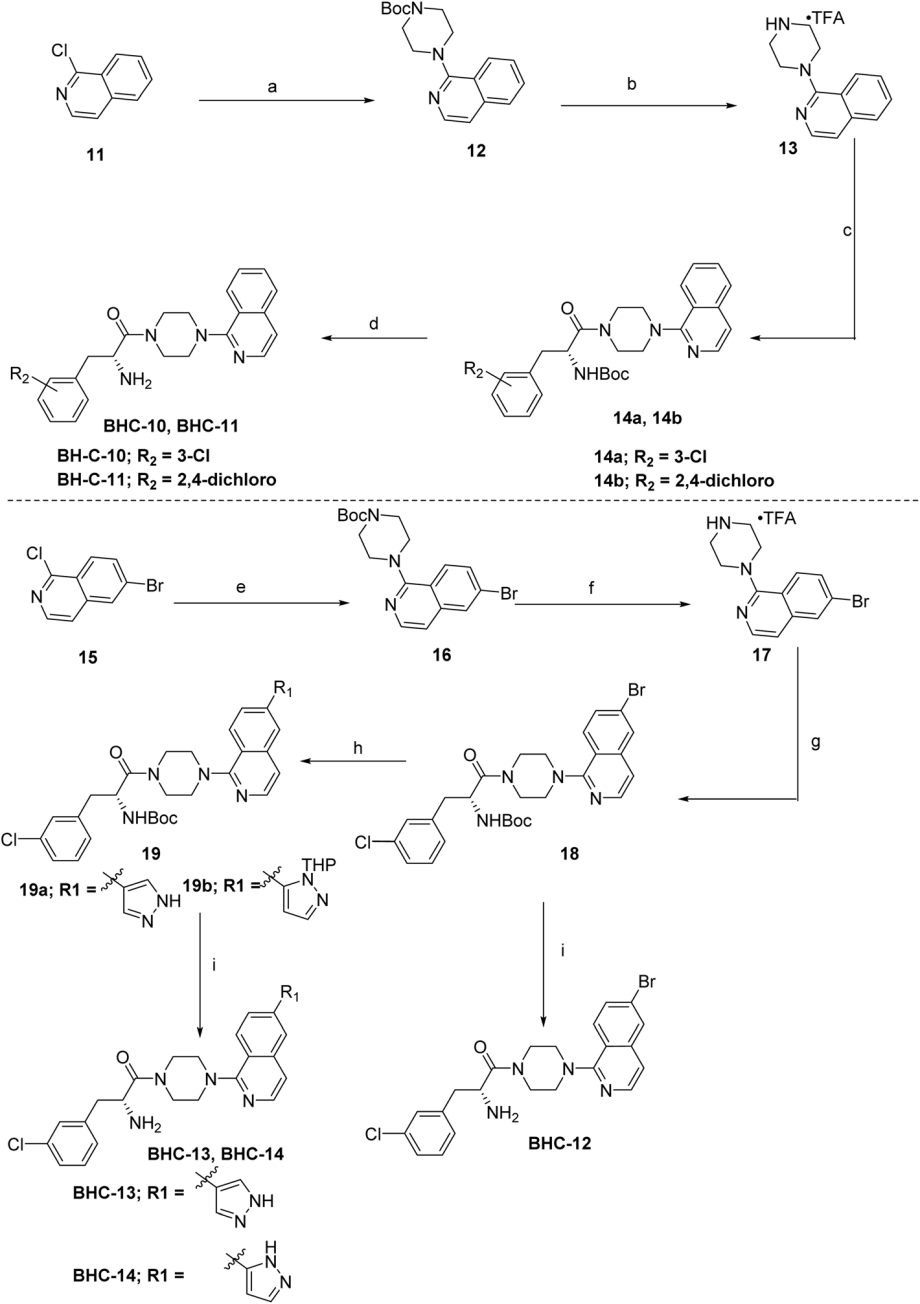
(A) The synthetic route for compounds BHC-10 & BHC-11. Reagents and conditions: (a) *tert*-butyl piperazine-1-carboxylate, Cs_2_CO_3_, DMF, 90 °C, 16 h; (b) TFA, DCM, RT, 4 h; (c) HATU, DIPEA, DMF, RT, 2 h (d) 4N HCl in 1,4-dioxane, RT, 2 h; (B) the synthetic route for compounds BHC-12. BHC-13 & BHC-14. Reagents and conditions: (e) *tert*-butyl piperazine-1-carboxylate, Cs_2_CO_3_, DMF, 90 °C, 16 h; (f) TFA, DCM, RT, 4 h; (g) HATU, DIPEA, DMF, RT, 2 h (h) corresponding boronic acid or boronic ester, Cs_2_CO_3_, Pd(dppf)Cl_2_. DCM, 1,4-dioxane, H_2_O, 90 °C, 16 h; (i) 4N HCl in 1,4-dioxane, RT, 2 h.

**Scheme 4:**
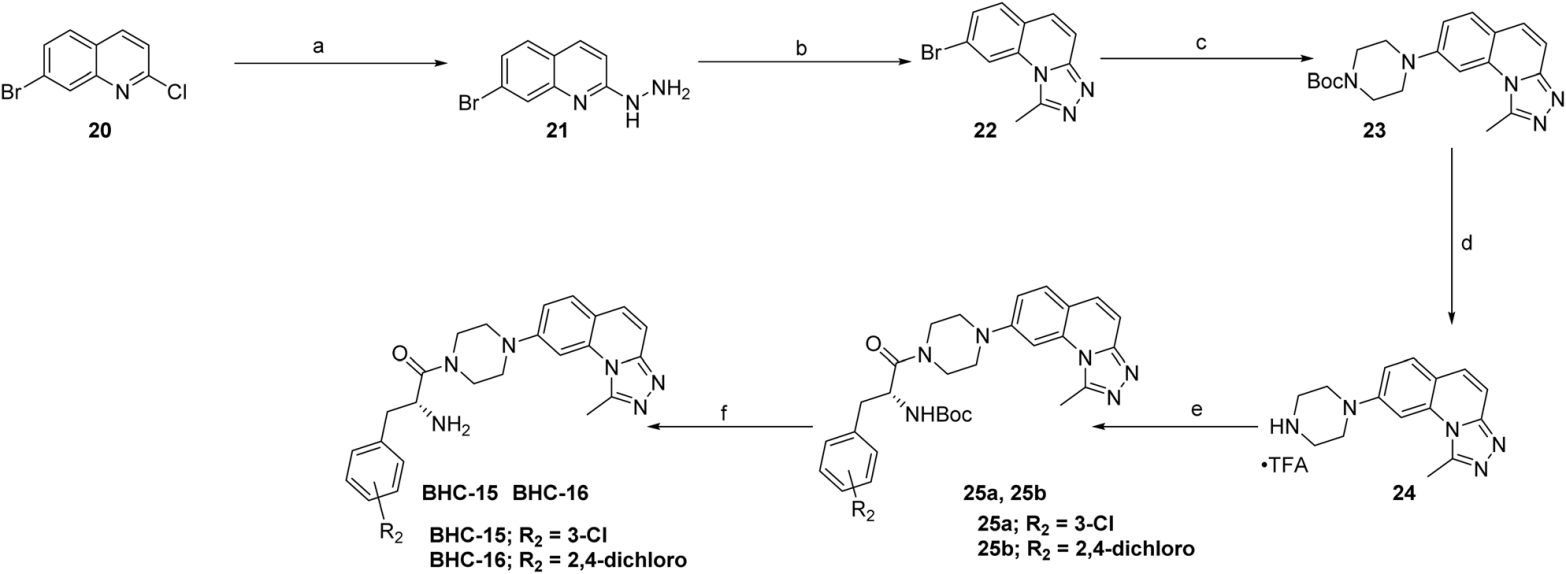
The synthetic route for compounds BHC-15 & BHC-16. Reagents and conditions: (a) NH_2_NH_2_ H_2_O, 100 °C, 16 h; (b) CH_3_COOH, 120 °C, 16 h; (c) *tert*-butyl piperazine-1-carboxylate, Pd_2_dba_3_, xantphos, Cs_2_CO_3_, 1,4-dioxane, 110 °C, 16 h; (d) TFA, CH_2_Cl_2_, RT, 4 h; (e) corresponding carboxylic acid, HATU, DIPEA, DMF, RT, 2 h; (f) 4N HCl in 1,4-dioxane, RT, 2 h.

**Scheme 5:**
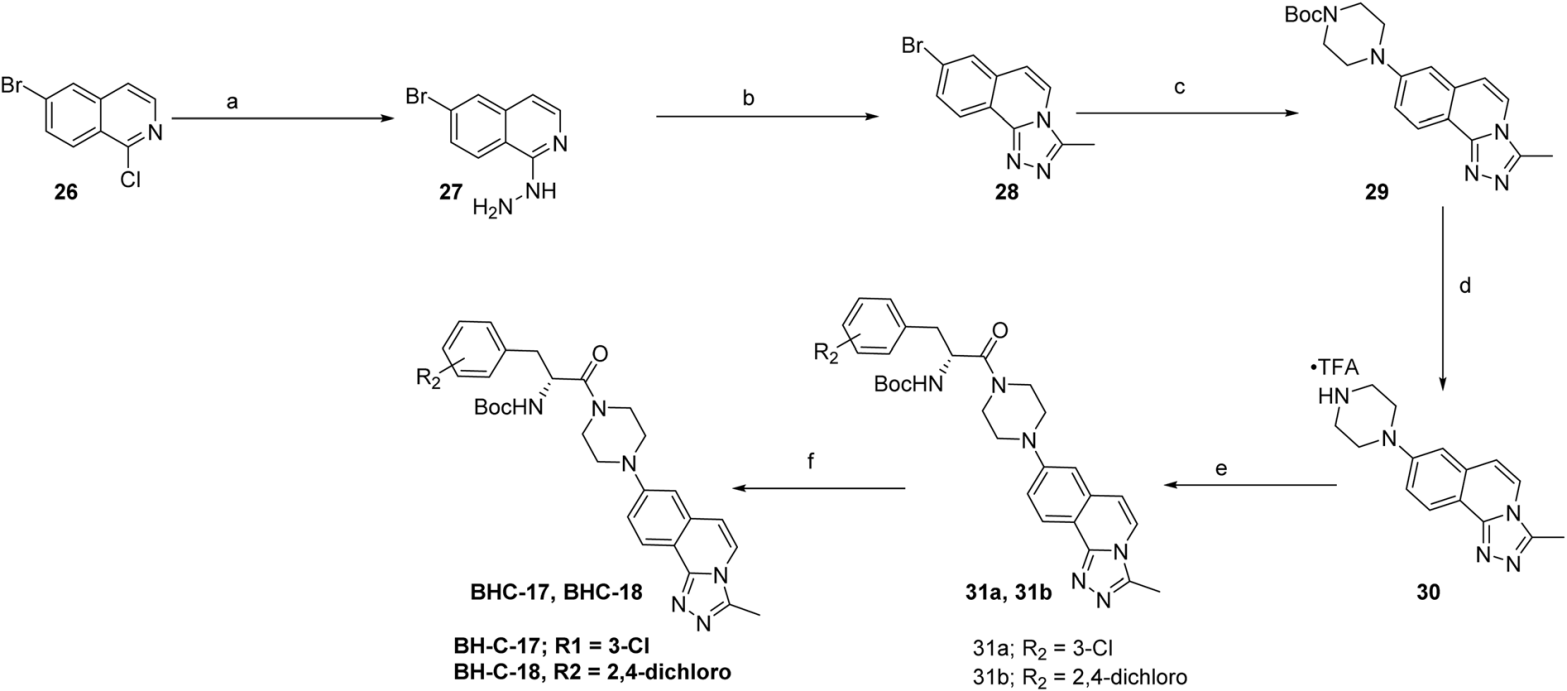
The synthetic route for compounds BHC-17 & BHC-18. Reagents and conditions: (a) NH_2_NH_2_ H_2_O, 100 °C, 16 h; (b) CH_3_COOH, 120 °C, 16 h; (c) *tert*-butyl piperazine-1-carboxylate, Pd_2_dba_3_, xantphos, Cs_2_CO_3_, 1,4-dioxane, 110 °C, 16 h; (d) TFA, CH_2_Cl_2_, RT, 4 h; (e) corresponding carboxylic acid, HATU, DIPEA, DMF, RT, 2 h; (f) 4N HCl in 1,4-dioxane, RT, 2 h.

**Scheme 6:**
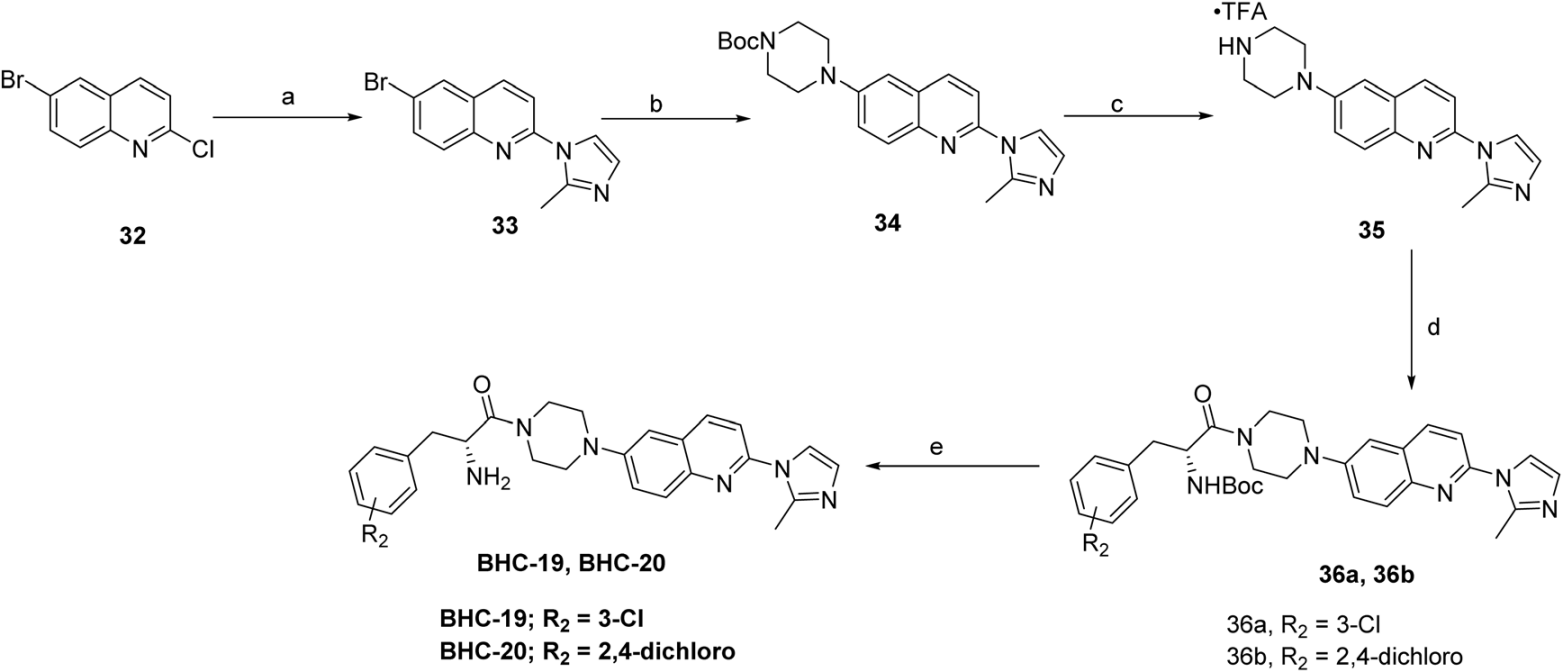
The synthetic route for compounds BHC-19 & BHC-20. Reagents and conditions: (a) 2-methyl imidazole, Cs_2_CO_3_, DMF, 90 °C, 2 h; (b) *tert*-butyl piperazine-1-carboxylate, Pd_2_dba_3_, xantphos, Cs_2_CO_3_, 1,4-dioxane, 110 °C, 16 h; (c) TFA, CH_2_Cl_2_, RT, 4 h; (d) corresponding carboxylic acid, HATU, DIPEA, DMF, RT, 2 h; (e) 4N HCl in 1,4-dioxane, RT, 2 h.

**Scheme 7:**
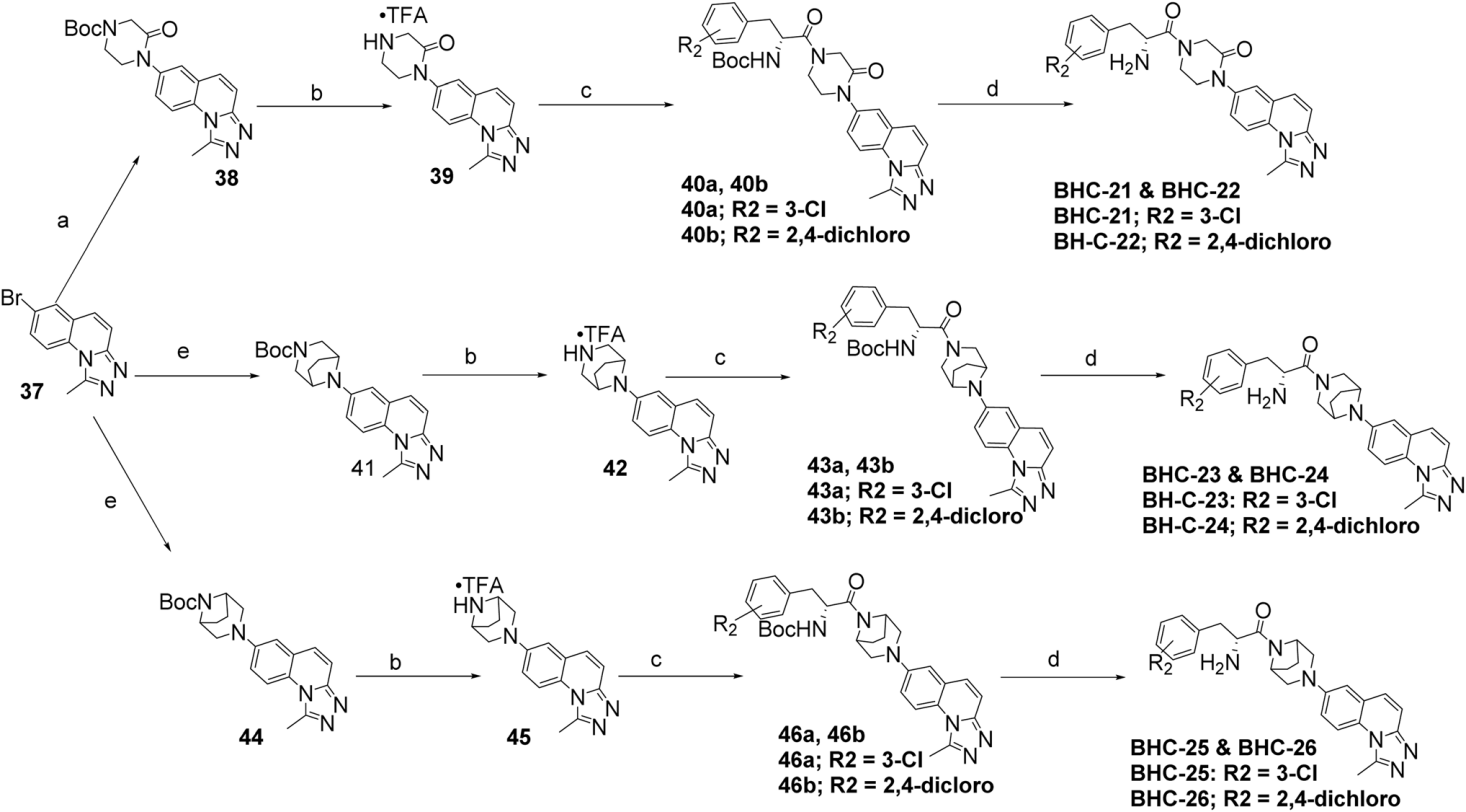
The synthetic route for compounds BHC-21 to BHC-26. Reagents and conditions: (a) *tert*-butyl 3-oxopiperazine-1-carboxylate, CuI, TEMDA, Cs_2_CO_3,_ DMF, 120 °C, 16 h (b) TFA, CH_2_Cl_2_, RT, 4 h; (c) corresponding carboxylic acid, HATU, DIPEA, DMF, RT, 2 h; (d) 4N HCl in 1,4-dioxane, RT, 2 h; (e) corresponding amine, Pd_2_dba_3_, xantphos, Cs_2_CO_3_, 1,4-dioxane, 110 °C, 16 h.

The synthesis of designed compounds BHC-1 to BHC-26 was illustrated in Schemes 1–7.

BHC-1 to BHC-4 were synthesized from 6-bromo-2-chloroquinoline. BHC-5 to BHC-9 were synthesized from 7-bromo-2-chloroquinoline. BHC-10 and BHC-11 were synthesized from 1-chloroisoquinoline. BHC-12 to BHC-14 were synthesized from 6-bromo-1-chloroisoquinoline. BHC-15 and BHC-16 were synthesized from 7-bromo-2-chloroquinoline. BHC-17 and BHC-18 were synthesized from 6-bromo-1-chloroisoquinoline. BHC-19 and BHC-20 were synthesized from 6-bromo-2-chloroquinoline. BHC-21 to BHC-26 were synthesized from 7-bromo-1-methyl-[1,2,4]triazolo[4,3-*a*]quinoline. The general procedures for synthesising these compounds are outlined in the SI.

## 4. HDAC8 inhibition and iso-enzyme selectivity

All twenty-six compounds were screened for HDAC8 inhibitory activity; the results are listed in Tables 1 and 2. Among these, eight compounds (BHC-1, BHC-2, BHC-3, BHC-5, BHC-6, BHC-10, BHC-12, BHC-13 and BHC-17) showed promising HDAC8 inhibitory activity below 500 nM. Compounds BHC-10 and BHC-13 exhibited significant HDAC8 inhibitory activity at concentrations below 100 nM. Among these twenty-six compounds, we observed that two compounds (BHC-11 and BHC-19) were precepted out in the enzymatic inhibitory experiment, and four compounds (BHC-4, BHC-7, BHC-8, BHC-9, and BHC-14) showed high autofluorescence. SAR analysis revealed that the nitrogen position in the quinoline was optimal for compounds BHC-10 and BHC-13 to capture additional interactions. Designs BHC-21 to BHC-26, which have substituted piperazine, lost activity when compared with the parent compound; this may be due to clashes with the HDAC8 protein.

**Table 1:** HDAC8 inhibitory activity of compounds BHC-1 to BHC-14

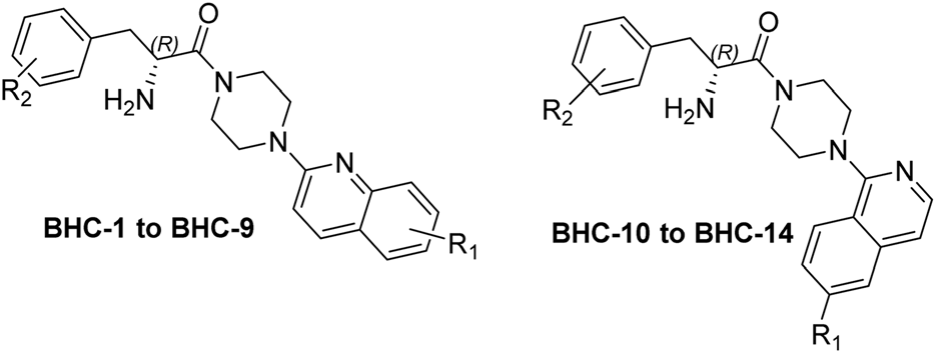				
Compound code	*R*_1_	*R*_2_	HDAC8 aIC_50_ (µM)	HDAC4 aIC_50_ (µM)
BHC-1	6-Br	3-Cl	0.51 ± 0.11	>35
BHC-2	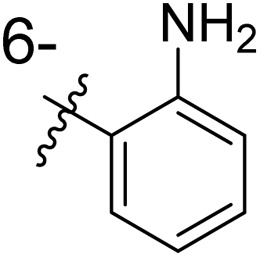	3-Cl	0.30 ± 0.08	>35
BHC-3	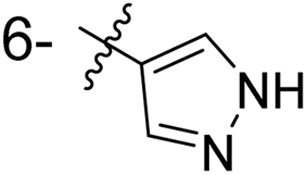	3-Cl	0.14 ± 0.05	>35
BHC-4	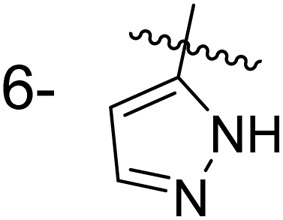	3-Cl	ND*	ND*
BHC-5	7-Br	3-Cl	0.28 ± 0.04	>35
BHC-6	7-Br	2,4-Dichloro	0.45 ± 0.13	>35
BHC-7	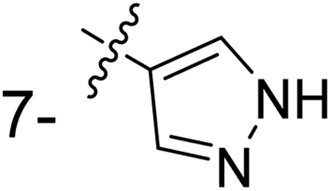	3-Cl	ND*	ND*
BHC-8	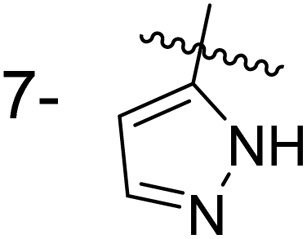	3-Cl	ND*	ND*
BHC-9	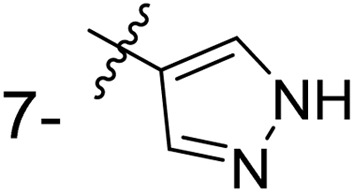	2,4-Dichloro	ND*	ND*
BHC-10	H	3-Cl	0.030 ± 0.009	>35
BHC-11	H	2,4-Dichloro	ND	ND
BHC-12	Br	3-Cl	0.12 ± 0.01	>35
BHC-13	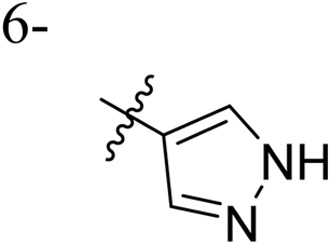	3-Cl	0.042 ± 0.013	>35
BHC-14	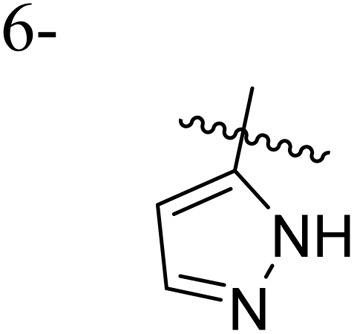	3-Cl	ND*	ND*
Compound B			0.20 ± 0.03	>35

a IC_50_ values are presented as the mean ± SD. The assay was performed in triplicate. ND = not determined; ND* = not determined due to high autofluorescence of the compound.

**Table 2:** HDAC8 inhibitory activity of compounds BHC-15 to BHC-26

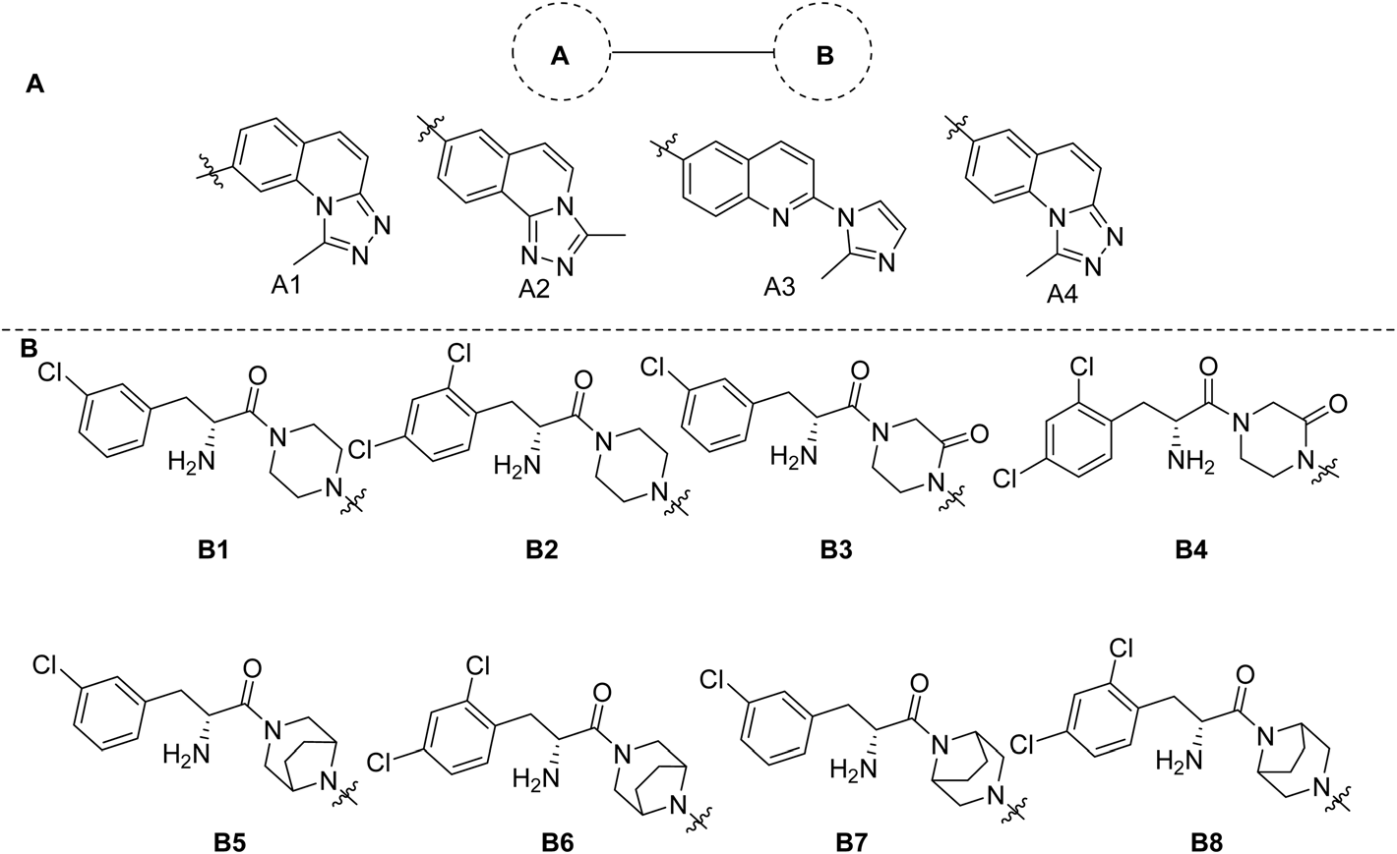				
Compound code	A	B	HDAC8 aIC_50_ (µM)	HDAC4 aIC_50_ (µM)
BHC-15	A1	B1	>35	>35
BHC-16	A1	B2	>35	>35
BHC-17	A2	B1	0.34 ± 0.02	>35
BHC-18	A2	B2	3.2 ± 0.3	>35
BHC-19	A3	B1	ND	ND
BHC-20	A3	B2	>35	>35
BHC-21	A4	B3	>35	>35
BHC-22	A4	B4	14 ± 3	>35
BHC-23	A4	B5	12 ± 0.2	>35
BHC-24	A4	B6	10 ± 0.2	>35
BHC-25	A4	B7	7 ± 1	>35
BHC-26	A4	B8	9 ± 1	>35
Compound B			0.20 ± 0.03	>35

a IC_50_ values are presented as the mean ± SD. The assay was performed in triplicate. ND = not determined.

We further evaluated the selectivity of all these compounds against HDAC4, a member of class IIa. None of the tested compounds was active against the HDAC4, demonstrating the selectivity of these compounds. We selected the most active compounds in HDAC8 inhibtion for further sectivity over other HDAC enzymes (HDAC1, HDAC6, HDAC10 and HDAC11). All the test compounds highly selective HDAC8 as shown in Table 3.

**Table 3:** Isoenzyme selectivity of most potent HDAC8 inhibitors

aIC_50_ (µM)							
Compound code	HDAC1 (class I)	HDAC3 (class I)	HDAC4 (class IIa)	HDAC6 (class IIb)	HDAC8 (class I)	HDAC10 (class IIb)	HDAC11 (class IV)
BHC-2	36 ± 2	6.9 ± 0.5	>35	>35	0.30 ± 0.08	>35	>35
BHC-3	>35	>35	>35	>35	0.14 ± 0.05	>35	>35
BHC-10	16 ± 1	1.9 ± 0.5	>35	>35	0.030 ± 0.009	>35	>35
BHC-12	11.2 ± 0.1	3.7 ± 0.8	>35	>35	0.12 ± 0.01	>35	>35
BHC-13	>35	2.5 ± 0.6	>35	>35	0.042 ± 0.013	>35	>35
Cpd B	1.7^b^	1.1 ± 0.2	ND	>30^b^	0.20 ± 0.03	ND	ND

a IC_50_ values are presented as the mean ± SD. The assay was performed in triplicate.

b Reported values^36^ for compound B. ND = not determined.

The results of HDAC enzyme analysis revealed that halogen substitution plays a crucial role in HDAC8 inhibition. Compounds with a mono-chloro (3-Cl) group at *R*_2_ and hydrogen at *R*_1_ display the strongest inhibitory activity, suggesting that these modifications are optimal for core scaffold potency. Additional halogenation (–Br) or bulky groups (pyrazole/aniline/fused 1,2,4-triazole) at *R*_1_, as well as di-chloro substitution at *R*_2_, tend to diminish activity. Most derivatives exhibited marked HDAC8 selectivity, as their activity against other HDAC isoforms was negligible. Thus, the strategic placement of halogen atoms—particularly a single 3-chloro substituent at *R*_2_ and hydrogen at *R*_1_—is key for maximizing HDAC8 inhibition and selectivity.

## 5. *In Vitro* antiproliferative effects of the representative compounds

To evaluate the cellular effects of the most active HDAC inhibitors (BHC-1, BHC-2, BHC-3, BHC-5, BHC-6, BHC-10, BHC-12, BHC-13, and BHC-17), we assessed their *in vitro* antiproliferative activities in a panel of human cancer cell lines (IMR-32, SH-SY5Y, and FaDu) along with a normal cell line (HEK293T). The evaluation was conducted using the highly sensitive WST-1 cell proliferation assay, which measures the mitochondrial dehydrogenase-mediated conversion of a tetrazolium salt into formazan.^41,42^ All tested compounds demonstrated significant antiproliferative effects across the cancer cell lines, as summarized in Table 4. Notably, compounds BHC-2 and BHC-10 showed prominent antiproliferative activity around 15 µM in IMR-32 and SH-SY5Y cell lines.

**Table 4:** Antiproliferative activity of representative compounds

Compound code	IMR-32 IC_50_ (µM)	SH-SY5Y IC_50_ (µM)	FaDu IC_50_ (µM)	Hek293-T IC_50_ (µM)
BHC-1	27 ± 2	21.9 ± 0.4	33 ± 1	27 ± 2
BHC-2	10.8 ± 0.1	13.2 ± 0.1	13.3 ± 0.2	56 ± 2
BHC-3	36.3 ± 0.1	37 ± 2	63 ± 1	31 ± 3
BHC-5	26 ± 3	27.5 ± 0.9	56.9 ± 0.8	58.6 ± 0.6
BHC-6	25 ± 1	22.6 ± 0.5	41 ± 4	29 ± 1
BHC-10	14.7 ± 0.1	10.6 ± 0.5	47.7 ± 0.6	47.7 ± 0.3
BHC-12	33 ± 2	37 ± 3	94 ± 3	35.0 ± 0.9
BHC-13	38 ± 2	40.0 ± 0.9	40 ± 4	31 ± 2
BHC-17	27 ± 1	33.4 ± 0.9	27 ± 1	24.4 ± 0.1
SAHA	1.62 ± 1.2	ND	6.81 ± 0.3	17.01 ± 5.8
Trichostatin A	8.24 ± 3.1	ND	10.2 ± 1.4	1.26 ± 0.3

Despite showing significant HDAC8 inhibitory activity, the effect of BHC-13 in the IMR-32 and SHS5Y cell lines was lower than that of BHC-10 and BHC-2. Based on their HDAC8 inhibitory activity and cellular potency, BHC-2, BHC-10, and BHC-13 were selected for further investigation.

## 6. Docking analysis

**Fig. 3:**
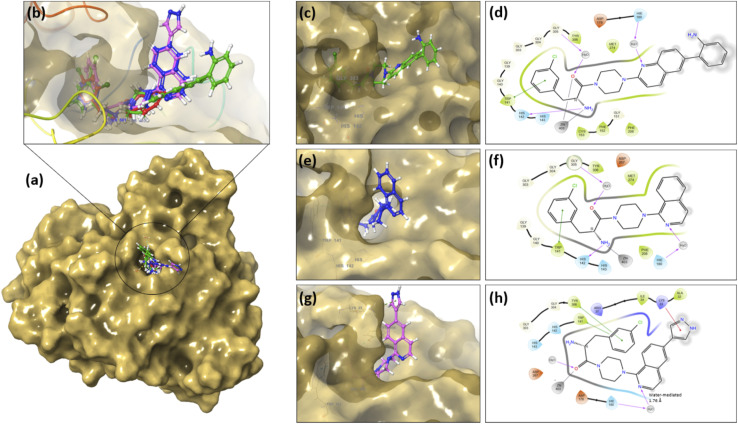
Representation of interactions between the HDAC8 protein (PDB: 3SFH) and the most active ligands; (a) represents the ligands BHC-2, BHC-10 and BHC-13 in the active site of the HDAC8; (b) represents the superimposition of active molecules on the cocrystal (red colour ligand in the figure) within the active site; (c and d) represents the binding pose and interactions of BHC-2; (e and f) represents the binding pose and interactions of BHC-10; and (g and h) represents the binding pose and interactions of BHC-13.

To understand their binding mode and interactions with HDAC8 protein, molecular docking studies were performed on these three compounds (BHC-2, BHC-10 & BHC-13). The compound BHC-2, with the docking score of −10.786 kcal mol^−1^, and MMGBSA score of −92.59 kcal mol^−1^, showed several interactions between the alpha amino amide and the active site residues His142 (H-bond, 1.97 Å), and Gly305 (water-mediated H-bond, 1.93 Å). In the acetate release channel, the 3-chlorophenyl ring exhibited π-stacking interactions with Trp141 (π-stacking, 3.71 Å). The nitrogen from the quinoline ring also participated in a water-mediated H-bond interaction with His180 (1.86 Å). Similar interactions are also seen with BHC-10 (docking score: −9.55 kcal mol^−1^; MMGBSA: −82.80 kcal mol^−1^), while BHC-13 (docking score: −7.897 kcal mol; MMGBSA: −52.66 kcal mol^−1^) has an additional interaction with the imidazole ring substituted on the 6th position of the isoquinoline ring. The results of the docking interactions are depicted in Fig. 3.

**Fig. 4:**
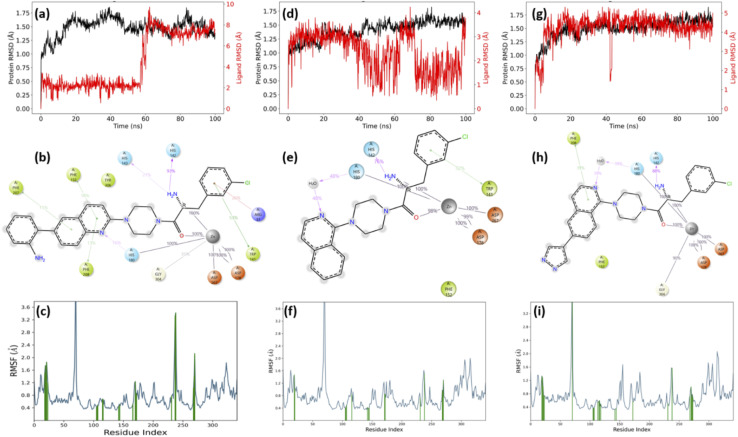
Representation of molecular dynamics results of the most active compounds; figures (a–c) represent the RMSD plot, percent residual contribution, and RMSF of the BHC-2 complex; (d–f) represent the RMSD plot, percent residual contribution, and RMSF of the BHC-10 complex; and (g–i) represent the RMSD plot, percent residual contribution, and RMSF of the BHC-13 complex.

For the compounds BHC-2, BHC-10 and BHC-13, we further carried out molecular dynamics for 100 ns to determine their stability. Upon subjecting the BHC-2 for a dynamic simulation, it is observed that the ligand RMSD is stable for the initial 55 ns with deviations between 1.5 Å and 6.0 Å, later it increased to 9.0 Å till 65 ns, and then remained stable until the end with deviations ranging between 7.0 Å and 9.0 Å, while the protein was stable for the entire simulations with RMSD ranging between 0.8 Å and 1.8 Å (Fig. 4a). The increased RMSD between 55 ns and 64 ns is due to the formation of several new interactions with Arg37, His143, Phe178, and Gly304. The residues that primarily contributed to the interactions include His142 (H-bond, 93%) and Trp141 (π-cation, 53%). The other interacting residues were less than moderate with stable residual fluctuations (Fig. 4b and c). In the case of BHC-10, the entire RMSD plot shows no major deviations with its RMSD ranging between 1.0 Å and 4.0 Å (Fig. 4d). The residues that majorly contributed in the interactions include Trp141 (π–π, 32%), His142 (H-bond, 76%), and His180 (water-mediated H-bond, 48%) with fewer active site residue fluctuations (Fig. 4d and e). The compound BHC-13 has the most stable RMSD plot with deviations ranging between 3.6 Å and 5.0 Å. The residues that majorly contributed to interactions include His148 (H-bond, 88%), His180 (water-mediated H-bond, 39%), and Phe208 (π–π, 39%) (Fig. 4g and h). The RMSF plot for the three complexes shows active site fluctuations below 2.0 Å, indicating the stability of the complexes.

Our simulation studies are in agreement with the biological activity, as the compound BHC-13 exhibits the most stable dynamics profile and is the most active in terms of biological activity, followed by BHC-10 and BHC-2.

## 7. Colony formation assay

A clonogenic assay was performed in the IMR-32 cell line to assess the long-term effect of these three compounds. The concentrations used in the colony formation assay were selected based on the respective anti-proliferative IC_50_ values of each compound (Table 4). For each compound, one concentration was chosen close to its IC_50_ value, while a second concentration was included to assess concentration-dependent effects.

**Fig. 5:**
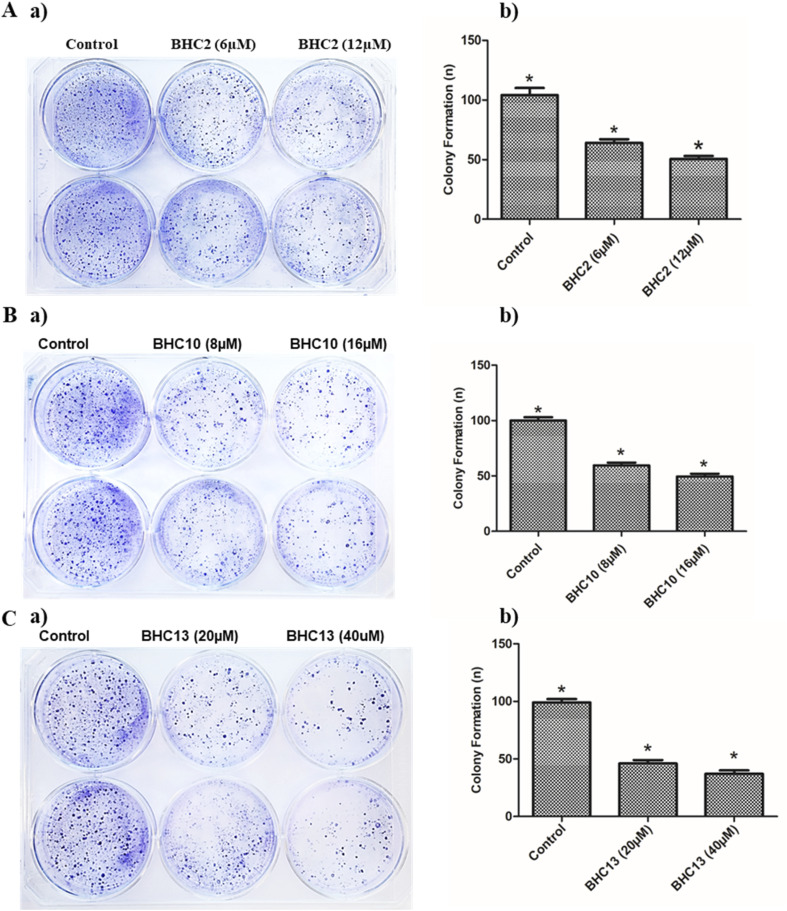
Effect of BHC-2, BHC-10, and BHC-13 on colony formation in IMR-32 cells. (A) BHC-2: representative images showing the impact of BHC-2 treatment on colony formation compared with control cells and quantification of colony numbers in control and treated cells. (B) BHC-10: representative images showing the effect of BHC-10 treatment on colony formation compared with control cells and quantification of colony numbers in control and treated cells. (C) BHC-13: representative images showing the effect of BHC-13 treatment on colony formation compared with control cells. And quantification of colony numbers in control and treated cells. Data are presented as mean ± SEM from three independent experiments. *p < 0.05 *versus* control.

All three compounds significantly reduced colony formation of IMR-32 cells in a dose-dependent manner. BHC-2 decreased colony formation by 36% and 50% at 6 µM and 12 µM, respectively, relative to DMSO-treated controls. BHC-10 reduced colony formation by ∼40% at 8 µM and ∼51% at 16 µM. BHC-13 exhibited the greatest inhibitory effect, reducing colonies by ∼53% and ∼63% at 20 µM and 40 µM, respectively, compared to the control (Fig. 5). These findings indicate a strong anticancer effect of these three compounds.

## 8. Effect of BHC-2, BHC-10 and BHC-13 to induce cell apoptosis in IMR32 cells

**Fig. 6:**
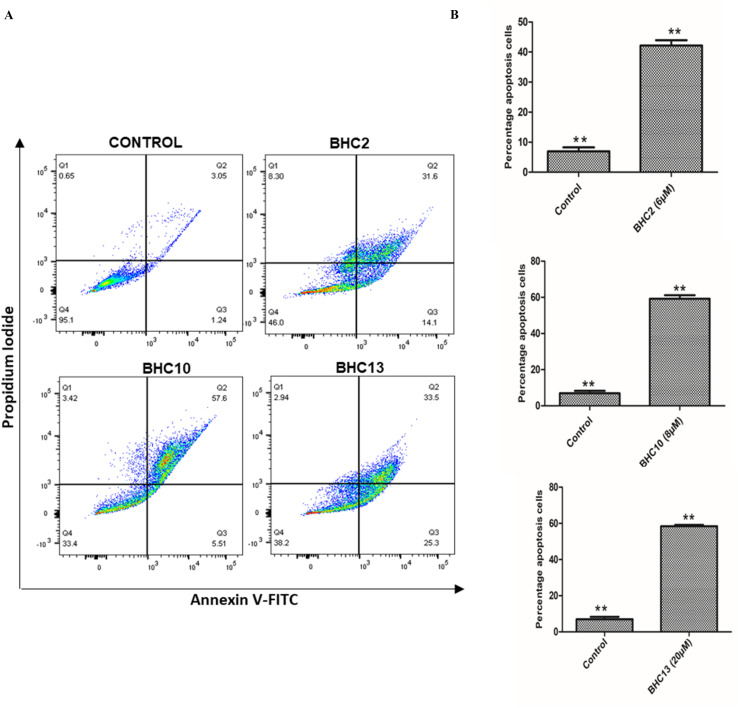
Effect of BHC-2, BHC-10, and BHC-13 on apoptosis in IMR-32 cells. (A) Representative flow cytometry plots showing apoptotic cell populations in control and treated groups. (B) Quantification of apoptosis percentage in IMR-32 cells compared with control. Data are presented as mean ± SEM from three independent experiments. ★*p* < 0.05, ★★*p* < 0.01 *versus* control.

**Fig. 7:**
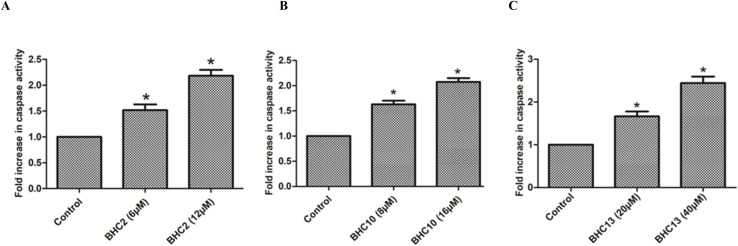
Caspase-3/7 activity in IMR-32 cells treated with increasing concentrations of (A) compound BHC-2 (B) compound BHC-10, and (C) compound BHC-13. Treatment induced apoptosis in a dose-dependent manner at 24 h post-treatment. *p < 0.05 compared with the control group.

To evaluate the pro-apoptotic potential of the most active compounds, BHC-2, BHC-10, and BHC-13, we performed Annexin V-FITC/propidium iodide (PI) staining and caspase 3/7 activity in IMR-32 cell lines. Annexin V/PI assay demonstrated ∼51.7%, 63%, and 57% increase in apoptosis by BHC-2, BHC-10, and BHC-13, respectively, compared to vehicle controls (Fig. 6). Treatment with the BHC-2 compound resulted in a significant, dose-dependent increase in caspase 3/7 activity, approximately 1.51-fold and 2.18-fold at 6 µM and 12 µM, respectively, compared to the vehicle control. Similarly, BHC-10 induced ∼1.62-fold and ∼2.04-fold increases in caspase 3/7 activity at 8 µM and 16 µM, respectively. BHC-13 also promoted apoptosis, as indicated by 1.66-fold and 2.44-fold elevations in caspase 3/7 activity at concentrations of 20 µM and 40 µM, respectively (Fig. 7). These findings suggest that all three compounds effectively activate apoptotic signaling in a dose-dependent manner.

## 9. Effect of BHC-2, BHC-10 and BHC-13 on cell cycle arrest

**Fig. 8:**
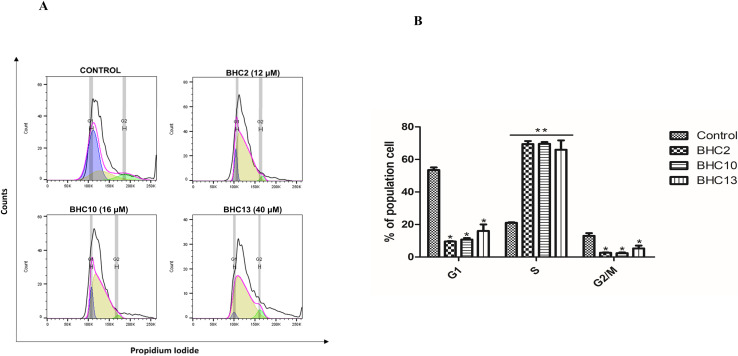
Effect of compounds on cell cycle progression in IMR-32 cells. (A) Representative flow cytometry histograms of IMR-32 cells treated with BHC-2, BHC-10, and BHC-13 for 24 h. (B) Quantitative analysis of cell cycle distribution following compound treatment. The percentage of cells in G1, S, and G2/M phases was determined using FlowJo software based on PI-stained DNA histograms. Data represent the mean ± SEM from three independent experiments, showing the differential accumulation of cells across phases upon treatment. Statistical significance was assessed (**p < 0.05).

Since BHC-2, BHC-10, and BHC-13 were identified as the most potent compounds in suppressing cancer cell proliferation and inducing apoptosis, we next examined whether they also influenced cell cycle progression. IMR-32 cells were treated with BHC-2 (12 µM), BHC-10 (16 µM), and BHC-13 (40 µM) for 24 h, and FACS analyzed cell cycle distribution following PI staining. As shown in the Fig. 8, all three compounds caused a significant accumulation of cells in the S phase, accompanied by a concomitant decrease in both G1 and G2/M populations compared to control cells. These results indicate that BHC-2, BHC-10, and BHC-13 induce S-phase arrest in IMR-32 cells, thereby contributing to their antiproliferative effects (Fig. 8).

## 10. Western blot analysis

**Fig. 9:**
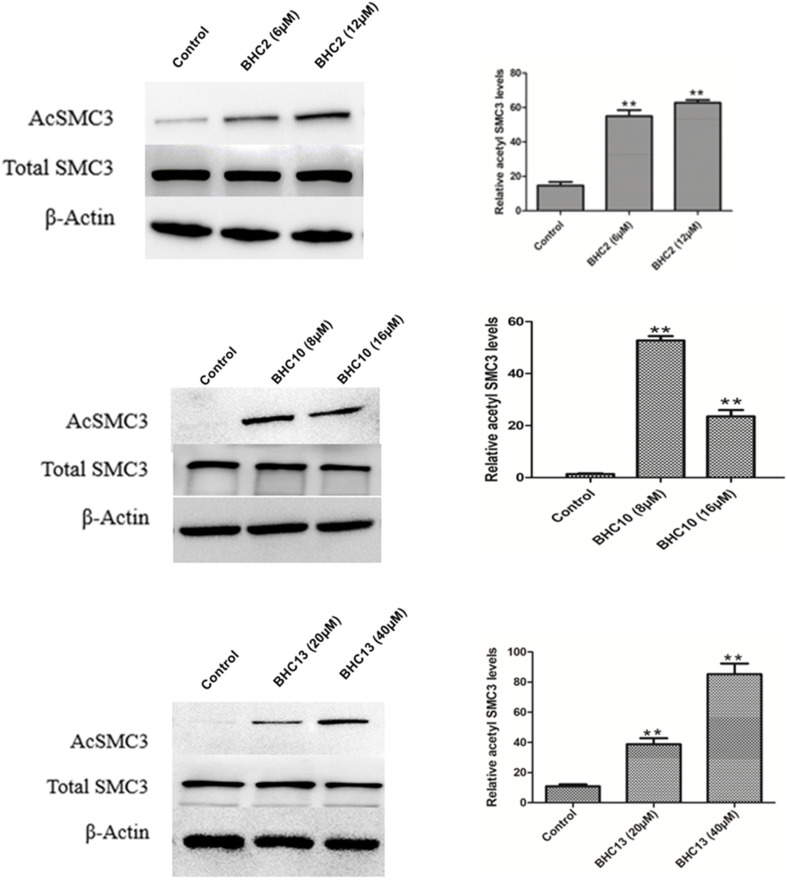
Western blot analysis showing the effect of BHC-2, BHC-10, and BHC-13 on SMC3 acetylation and total SMC3 levels. (Top panels) BHC-2 treatment at 6 µM and 12 µM: representative blot and densitometric analysis. (Middle panels) BHC-10 treatment at 8 µM and 16 µM: its representative blot and densitometric analysis. (Lower panels) BHC-13 treatment at 20 µM and 40 µM: its representative blot and densitometric analysis. Densitometric quantification was performed using ImageJ; data represent mean ± SD from three independent experiments (**p < 0.05).

Acetylation of SMC3 is regulated by ESCO1/2 and HDAC8. Specifically, ESCO1/2 catalyzes the acetylation of Lys105 and Lys106 residues during the S phase, while HDAC8 removes acetylation.^43^ Inhibition of HDAC8 activity leads to the accumulation of acetylated SMC3 (Ac-SMC3). Elevated levels of Ac-SMC3 enhance the stability of cohesin–DNA interactions, which can facilitate accurate chromosome segregation, maintain genome integrity, and support DNA damage repair.^44,45^ To evaluate the impact of compounds BHC-2, BHC-10, and BHC-13 on SMC3 acetylation, IMR-32 cells were treated with different concentrations of compound BHC-2 (6 µM & 12 µM), BHC-10 (8 µM & 16 µM), and BHC-13 (20 µM & 40 µM), for 24 hours. Western blot analysis was then performed using antibodies specific for acetylated SMC3 and total SMC3. Treatment with all three compounds (BHC-2, BHC-10, BHC-13) increased Ac-SMC3 levels, indicating enhanced acetylation of SMC3 (Fig. 9).

Importantly, total SMC3 protein levels remained unchanged across all the compounds, suggesting that these compounds specifically modulate the acetylation status without altering overall SMC3 expression. Although our findings demonstrate that BHC-2, BHC-10, and BHC-13 exhibit potent anticancer activity and induce increased acetylation of SMC3, a well-established substrate of HDAC8, this observation represents a pharmacodynamic marker and functional readout consistent with HDAC8 inhibition rather than direct evidence of intracellular target engagement. Together with the biochemical HDAC8 inhibition data, the accumulation of acetylated SMC3 supports HDAC8 as the primary molecular target underlying the observed antiproliferative and pro-apoptotic effects. Nevertheless, we cannot exclude the possibility that additional molecular targets contribute to the biological activity of these compounds. Comprehensive intracellular target-engagement studies, including the cellular thermal shift assay (CETSA), affinity-based target identification approaches, and proteome-wide selectivity profiling, would provide deeper mechanistic insights into target engagement, selectivity, and potential off-target interactions. Although these investigations are beyond the scope of the present study, they represent important future directions for validating the mechanism of action, establishing the selectivity profile of these compounds, and supporting their continued preclinical development as HDAC8-targeted anticancer agents.

## 11. Conclusion

We designed and synthesized series of quinoline derivatives as HDAC8 inhibitors, Compound BHC-10 and BHC-13 showed significant HDAC8 inhibitory activity below 100 nM. Compounds BHC-2 and BHC-10 showed significant anticancer activity in neuroblastoma cancer cell lines. Further evaluation of BHC-2, BHC-10 and BHC-13 provided the therapeutic potential of these compounds as anticancer agents in neuroblastoma cell lines. These non-hydroxamate-based inhibitors show considerable promise for treating cancers with high HDAC8 expression. Continued structural optimization, guided by docking and dynamics insights, could enhance their potency and facilitate the development of lead compounds for future cancer therapies.

## 12. Experimental section

The detailed procedures for preparing intermediates are provided in the SI.

### 12.1 Chemistry

#### 12.1.1 General

All the chemical reagents and solvents were purchased from commercial suppliers and used without further purification. All reactions were performed under an argon atmosphere in oven-dried glassware with magnetic stirring. All reactions were monitored by thin-layer chromatography (TLC) on 0.25 mm silica gel plates (60GF-254), and developed chromatograms were visualized by UV light. Proton nuclear magnet resonance (^1^H NMR) spectra were recorded at 400 or 500 MHz on Varian or Bruker instrumentation. ^13^C NMR spectra were recorded at 101 MHz (Varian). Trimethylsiane (TMS) or residual solvent signals were used as an internal reference to calibrate the chemical shift *δ* values, and *δ* values were expressed in parts per million (ppm). Coupling constant *J* values were reported in hertz (Hz). The multiplicity of ^1^H NMR signals were reported as singlet (s), doublet (d), doublet of doublet (dd), triplet (t), quartert (q), broad singlet (brs), and multiplet (m). Liquid chromatography-mass spectra (LCMS) was obtained from Agilent 1100-LC/MSDVL. The purity of the final compounds was determined by high-performance liquid chromatography (HPLC). Purity was measured by UV absorbance at 254 nM.

#### 12.1.2 General procedure for the synthesis of final compounds BHC-1 to BHC-26

4M HCl in 1,4-dioxane (4 equiv.) was added to the stirred solution of prefinal compounds (1 equiv.) in 1,4-dioxane (10 volumes) at °C and then stirred at RT for 2 h. The progress of the reaction was monitored by TLC. After completion of the reaction, as indicated by TLC, the reaction mixture was concentrated under reduced pressure. The resultant crude was neutralised with saturated NaHCO_3_ solution and extracted with EtOAc. Dried the organic layer over anhydrous Na_2_SO_4_ and concentrated under reduced pressure. The resultant crude was purified using combiflash to afford the final compounds.

##### 12.1.1.1 (*R*)-2-Amino-1-(4-(6-bromoquinolin-2-yl)piperazin-1-yl)-3-(3-chlorophenyl)propan-1-one (BHC-1)

Compound BHC-1 was obtained as a 45 mg (yield: 71%) pale yellow solid. Melting point: 172−174 °C; ^1^H NMR (400 MHz, DMSO-*d*_6_): δ ppm 8.05 (d, *J* = 9.6 Hz, 1H), 7.97 (d, *J* = 2.4 Hz, 1H), 7.63 (dd, *J* = 8.8, 2.4 Hz, 1H), 7.50 (d, *J* = 9.2 Hz, 1H), 7.33–7.27 (m, 3H), 7.25–7.21 (m, 2H), 3.99–3.97 (m, 1H), 3.71–3.69 (m, 2H), 3.63–3.55 (m, 4H), 3.47–3.41 (m, 2H), 2.82–2.80 (m, 1H), 2.71–2.68 (m, 1H), 2.02–1.98 (brs, 2H); ^13^C NMR (101 MHz, DMSO-*d*_6_): δ 173.47, 157.38, 146.40, 141.65, 137.18, 133.13, 132.72, 130.31, 129.78, 129.73, 128.76, 128.60, 126.57, 124.66, 114.52, 111.50, 51.87, 44.81, 44.62; HPLC: 95.26%; LCMS (ES, *m*/*z*) calculated for C_22_H_23_BrClN_4_O [(M + H)^+^] 473.07, observed 473.05.

##### 12.1.1.2 (*R*)-2-Amino-1-(4-(6-(2-aminophenyl)quinolin-2-yl)piperazin-1-yl)-3-(3-chlorophenyl) propan-1-one (BHC-2)

Compound BHC-2 was obtained as 52 mg (yield: 75%) of an off-white solid. Melting point: 175−177 °C; ^1^H NMR (400 MHz, DMSO-*d*_6_): δ ppm 8.10 (d, *J* = 9.2 Hz, 1H), 7.75 (d, *J* = 1.6 Hz, 1H), 7.65–7.58 (m, 2H), 7.35–7.22 (m, 5H), 7.08–7.04 (m, 2H), 6.79 (dd, *J* = 8.0, 1.4 Hz, 1H), 6.68–6.64 (m, 1H), 4.81 (s, 2H), 4.01 (t, *J* = 6.2, 1H), 3.75–3.69 (m, 2H), 3.66–3.47 (m, 5H), 3.39–3.37 (m, 1H), 2.86–2.81 (m, 1H), 2.72–2.68 (m, 1H), 1.91 (brs, 2H); ^13^C NMR (101 MHz, DMSO-*d*_6_): δ 173.47, 157.38, 146.40, 141.65, 137.18, 133.13, 132.72, 130.31, 129.78, 129.73, 128.76, 128.60, 126.57, 124.66, 114.52, 111.50, 51.87, 44.81, 44.62; HPLC: 96.20%; LCMS (ES, *m*/*z*) calculated for C_28_H_29_ClN_5_O [(M + H)^+^] 486.21, observed 486.15.

##### 12.1.1.3 (*R*)-1-(4-(6-(1*H*-Pyrazol-4-yl)quinolin-2-yl)piperazin-1-yl)-2-amino-3-(3-chlorophenyl) propan-1-one (BHC-3)

Compound BHC-3 was obtained as a pale yellow solid, 32 mg (yield: 40%). Melting point: 132−135 °C; ^1^H NMR (400 MHz, DMSO-*d*_6_): δ ppm 12.97 (s, 1H), 8.10 (s, 2H), 8.02 (d, *J* = 9.2 Hz, 1H), 7.93 (d, *J* = 2.0 Hz, 1H), 7.83 (dd, *J* = 8.8, 2.0 Hz, 1H), 7.57 (d, *J* = 8.8 Hz, 1H), 7.34–7.29 (m, 2H), 7.25–7.21 (m, 3H), 3.97 (t, *J* = 6.8 Hz, 1H), 3.72–3.48 (m, 8H), 2.85–2.80 (m, 1H), 2.72–2.66 (m, 1H), 1.74 (brs, 2H); ^13^C NMR (101 MHz, DMSO-*d*_6_): δ 173.54, 156.88, 146.25, 141.72, 137.69, 133.12, 130.30, 129.72, 128.75, 128.34, 127.49, 126.93, 126.55, 123.63, 122.86, 121.50, 110.87, 51.88, 45.07, 44.86, 41.77, 41.65; HPLC: 91.85%; LCMS (ES, *m*/*z*) calculated for C_25_H_26_ClN_6_O [(M + H)^+^] 461.19, observed 461.00.

##### 12.1.1.4 (*R*)-1-(4-(6-(1*H*-Pyrazol-5-yl)quinolin-2-yl)piperazin-1-yl)-2-amino-3-(3-chlorophenyl) propan-1-one (BHC-4)

Compound BHC-4 was obtained as a 25 mg (yield: 35%) pale yellow solid. Melting point: 143−135 °C; ^1^H NMR (400 MHz, DMSO-*d*_6_): δ ppm 12.88 (s, 1H), 8.12–8.08 (m, 3H), 7.81–7.78 (m, 1H), 7.61 (d, *J* = 8.8 Hz, 1H), 7.34–7.29 (m, 2H), 7.26–7.21 (m, 3H), 6.75 (s, 1H), 4.00 (t, *J* = 6.8 Hz, 1H), 3.74–3.64 (m, 2H), 3.62–3.56 (m, 4H), 3.51–3.38 (m, 2H), 2.84–2.80 (m, 1H), 2.71–2.68 (m, 1H), 1.95 (brs, 2H); ^13^C NMR (101 MHz, DMSO-*d*_6_): δ 173.48, 157.15, 147.15, 141.67, 138.10, 133.13, 130.31, 129.74, 128.76, 127.74, 126.78, 126.58, 123.71, 123.32, 110.94, 51.77, 45.00, 44.87, 44.78, 41.77, 41.66; HPLC: 90.36%; LCMS (ES, *m*/*z*) calculated for C_25_H_26_ClN_6_O [(M + H)^+^] 461.19, observed 461.20.

##### 12.1.1.5 (*R*)-2-Amino-1-(4-(7-bromoquinolin-2-yl)piperazin-1-yl)-3-(3-chlorophenyl)propan-1-one (BHC-5)

Compound BHC-5 was obtained as a 50 mg (yield: 67%) pale yellow solid. Melting point: 177−179 °C; ^1^H NMR (400 MHz, DMSO-*d*_6_): δ ppm 8.08 (d, *J* = 9.2 Hz, 1H), 7.74 (d, *J* = 2.0 Hz, 1H), 7.68 (d, *J* = 8.4 Hz, 1H), 7.38–7.31 (m, 2H), 7.29–7.21 (m, 4H), 4.03–3.98 (m, 1H), 3.74–3.69 (m, 2H), 3.62–3.55 (m, 4H), 3.48–3.42 (m, 2H), 2.86–2.81 (m, 1H), 2.72–2.67 (m, 1H), 2.38–2.12 (brs, 2H); ^13^C NMR (101 MHz, DMSO-*d*_6_): δ 173.30, 157.59, 148.58, 141.52, 137.95, 133.15, 130.33, 129.82, 129.74, 128.77, 128.24, 126.61, 125.47, 123.19, 122.02, 111.0, 51.82, 44.79, 44.74; HPLC: 97.75%; LCMS (ES, *m*/*z*) calculated for C_22_H_23_BrClN_4_O [(M + H)^+^] 473.07, observed 473.00.

##### 12.1.1.6 (*R*)-2-Amino-1-(4-(7-bromoquinolin-2-yl)piperazin-1-yl)-3-(2,4-dichlorophenyl)propan-1-one (BHC-6)

Compound BHC-6 was obtained as a pale yellow solid, 42 mg (yield: 62%). Melting point: 144−146 °C; ^1^H NMR (400 MHz, DMSO-*d*_6_): δ ppm 8.08 (d, *J* = 9.2 Hz, 1H), 7.74 (d, *J* = 1.6 Hz, 1H), 7.68 (d, *J* = 8.4 Hz, 1H), 7.57 (d, *J* = 2.0 Hz, 1H), 7.41–7.34 (m, 3H), 7.27 (d, *J* = 9.2 Hz, 1H), 4.03–4.01 (m, 1H), 3.74–3.66 (m, 2H), 3.64–3.59 (m, 4H), 3.49–3.42 (m, 2H), 2.95–2.89 (m, 1H), 2.82–2.77 (m, 1H), 2.03 (brs, 2H); ^13^C NMR (101 MHz, DMSO-*d*_6_): δ 173.30, 157.59, 148.58, 141.52, 137.95, 133.15, 130.33, 129.82, 129.74, 128.77, 128.24, 126.61, 125.47, 123.19, 122.02, 111.0, 51.82, 44.79, 44.74; HPLC: 97.24%; LCMS (ES, *m*/*z*) calculated for C_22_H_22_BrCl_2_N_4_O [(M + H)^+^] 507.04, observed 506.95.

##### 12.1.1.7 (*R*)-1-(4-(7-(1*H*-Pyrazol-4-yl)quinolin-2-yl)piperazin-1-yl)-2-amino-3-(3-chlorophenyl) propan-1-one (BHC-7)

Compound BHC-7 was obtained as a 35 mg (51% yield) pale yellow solid. Melting point: 121−123 °C; ^1^H NMR (400 MHz, DMSO-*d*_6_): δ ppm 13.00 (s, 1H), 8.32–8.06 (m, 2H), 8.01 (d, *J* = 8.8 Hz, 1H), 7.79 (s, 1H), 7.69 (d, *J* = 8.0 Hz, 1H), 7.55 (d, *J* = 8.4 Hz, 1H), 7.37–7.29 (m, 2H), 7.25–7.21 (m, 2H), 7.14 (d, *J* = 6.8 Hz, 1H), 4.00 (t, *J* = 6.8 Hz, 1H), 3.74–3.58 (m, 2H), 3.56–3.54 (m, 4H), 3.49–3.42 (m, 2H), 2.85–2.81 (m, 1H), 2.72–2.66 (m, 1H), 1.91 (brs, 2H); ^13^C NMR (101 MHz, DMSO-*d*_6_): δ 173.50, 157.47, 148.20, 141.67, 137.69, 134.60, 133.13, 130.31, 129.73, 128.75, 128.29, 126.57, 121.74, 121.60, 121.34, 121.08, 109.60, 51.87, 45.03, 44.88, 44.78, 41.76, 41.66; HPLC: 96.36%; LCMS (ES, *m*/*z*) calculated for C_25_H_26_ClN_6_O [(M + H)^+^] 461.19, observed 461.00.

##### 12.1.1.8 (*R*)-1-(4-(7-(1*H*-Pyrazol-5-yl)quinolin-2-yl)piperazin-1-yl)-2-amino-3-(3-chlorophenyl) propan-1-one (BHC-8)

Compound BHC-8 was obtained as a 52 mg (yield: 72%) pale yellow solid. Melting point: 181−183 °C; ^1^H NMR (400 MHz, DMSO-*d*_6_): δ ppm 13.00 (s, 1H), 8.05 (d, *J* = 8.8 Hz, 1H), 7.99 (s, 1H), 7.81–7.72 (m, 3H), 7.35–7.29 (m, 2H), 7.25–7.18 (m, 3H), 6.87 (d, *J* = 1.2 Hz, 1H), 4.00 (t, *J* = 6.8 Hz, 1H), 3.75–3.68 (m, 2H), 3.56–3.54 (m, 4H), 3.49–3.42 (m, 2H), 2.86–2.81 (m, 1H), 2.72–2.66 (m, 1H), 1.82 (brs, 2H); ^13^C NMR (101 MHz, DMSO-*d*_6_): δ 173.50, 157.53, 147.95, 141.68, 137.74, 133.14, 130.31, 129.73, 128.76, 128.18, 126.57, 122.70, 122.12, 120.49, 1.60, 51.87, 45.03, 44.88, 44.78, 41.76, 41.66; HPLC: 96.44%; LCMS (ES, *m*/*z*) calculated for C_25_H_26_ClN_6_O [(M + H)^+^] 461.19, observed 461.05.

##### 12.1.1.9 (*R*)-1-(4-(7-(1*H*-Pyrazol-4-yl)quinolin-2-yl)piperazin-1-yl)-2-amino-3-(2,4- dichlorophenyl)propan-1-one (BHC-9)

Compound BHC-9 was obtained as 62 mg (yield: 78%) of a pale yellow solid. Melting point: 132−134 °C; ^1^H NMR (400 MHz, DMSO-*d*_6_): δ ppm 13.02 (s, 1H), 8.37 (brs, 1H), 8.11 (brs, 1H), 8.01 (d, *J* = 9.2 Hz, 1H), 7.80 (s, 1H), 7.69 (d, *J* = 8.4 Hz, 1H), 7.59–7.53 (m, 2H), 7.42–7.35 (m, 2H), 7.15 (d, *J* = 8.4 Hz, 1H), 4.01 (t, *J* = 6.8 Hz, 1H), 3.73–3.57 (m, 6H), 3.49–3.42 (m, 2H), 2.95–2.90 (m, 1H), 2.82–2.79 (m, 1H), 1.84 (brs, 2H); ^13^C NMR (101 MHz, DMSO-*d*_6_): δ 173.43, 157.49, 148.20, 137.69, 135.72, 134.70, 134.60, 134.16, 132.24, 128.89, 128.29, 127.44, 121.76, 121.60, 121.36, 121.10, 109.63, 50.11, 45.07, 44.92, 44.75, 41.74; HPLC: 98.31%; LCMS (ES, *m*/*z*) calculated for C_25_H_25_Cl_2_N_6_O [(M + H)^+^] 495.15, observed 495.10.

##### 12.1.1.10 (*R*)-2-Amino-3-(3-chlorophenyl)-1-(4-(isoquinolin-1-yl)piperazin-1-yl)propan-1-one (BHC-10)

Compound BHC-10 was obtained as a 52 mg (yield: 63%) pale yellow solid. Melting point: 145−147 °C; ^1^H NMR (400 MHz, DMSO-*d*_6_): δ ppm 8.12–8.10 (m, 2H), 7.90 (d, *J* = 8.0 Hz, 1H), 7.74–7.70 (m, 1H), 7.64–7.59 (m, 1H), 7.41 (d, *J* = 7.6 Hz, 1H), 7.35–7.24 (m, 4H), 4.02–3.98 (m, 1H), 3.79–3.68 (m, 3H), 3.64–3.58 (m, 1H), 3.28–3.26 (m, 2H), 3.18–3.11 (m, 1H), 3.01–2.96 (m, 2H), 2.89–2.84 (m, 1H), 1.99 (brs, 2H); ^13^C NMR (101 MHz, DMSO-*d*_6_): δ 173.41, 160.79, 141.66, 140.87, 138.11, 133.14, 130.50, 130.28, 129.73, 128.74, 127.65, 127.12, 126.57, 125.62, 121.22, 116.43, 51.85, 51.52, 51.40, 45.21; HPLC: 99.0%;, LCMS (ES, *m*/*z*) calculated for C_22_H_24_ClN_4_O [(M + H)^+^] 395.16, observed 394.95.

##### 12.1.1.11 (*R*)-2-Amino-3-(2,4-dichlorophenyl)-1-(4-(isoquinolin-1-yl)piperazin-1-yl)propan-1-one (BHC-11)

Compound BHC-11 was obtained as a pale yellow solid, 52 mg (yield: 63%). Melting point: 145−147 °C; ^1^H NMR (400 MHz, DMSO-*d*_6_): δ ppm 8.13–8.10 (m, 2H), 7.90 (d, *J* = 7.6 Hz, 1H), 7.74–7.70 (d, *J* = 8.4 Hz, 1H), 7.64–7.58 (m, 2H), 7.42–7.36 (m, 3H), 4.16–4.12 (m, 1H), 3.75–3.68 (m, 3H), 3.59–3.54 (m, 1H), 3.28–3.25 (m, 3H), 3.21–3.17 (m, 1H), 3.07–3.02 (m, 2H), 2.87–2.82 (m, 1H), 2.69–2.61 (m, 1H); ^13^C NMR (101 MHz, DMSO-*d*_6_): δ 173.30, 157.59, 148.58, 141.52, 137.95, 133.15, 130.33, 129.82, 129.74, 128.77, 128.24, 126.61, 125.47, 123.19, 122.02, 111.0, 51.82, 44.79, 44.74; HPLC: 97.04%; LCMS (ES, *m*/*z*) calculated for C_22_H_23_Cl_2_N_4_O [(M + H)^+^] 429.12, observed 428.95.

##### 12.1.1.12 (*R*)-2-Amino-1-(4-(6-bromoisoquinolin-1-yl)piperazin-1-yl)-3-(3-chlorophenyl)propan-1-one (BHC-12)

Compound BHC-12 was obtained as a pale yellow solid, 46 mg (yield: 60%). Melting point: 132−134 °C; ^1^H NMR (400 MHz, DMSO-*d*_6_): δ ppm 8.19 (d, *J* = 2.0 Hz, 1H), 8.14 (d, *J* = 5.6 Hz, 1H), 8.03 (d, *J* = 8.8 Hz, 1H), 7.71 (dd, *J* = 8.8, 2.0 Hz, 1H), 7.39 (d, *J* = 5.6 Hz, 1H), 7.33–7.20 (m, 4H), 4.00–3.98 (m, 1H), 3.78–3.71 (m, 3H), 3.63–3.61 (m, 1H), 3.28–3.26 (m, 2H), 3.21–3.16 (m, 1H), 3.06–3.02 (m, 1H), 2.86–2.80 (m, 1H), 2.70–2.64 (m, 1H), 1.77 (brs, 2H); ^13^C NMR (101 MHz, DMSO-*d*_6_): δ 173.48, 160.87, 142.15, 141.70, 139.58, 133.13, 130.28, 130.02, 129.71, 129.58, 128.72, 128.10, 126.56, 124.25, 119.70, 115.36, 51.85, 51.44, 51.40, 45.13, 41.81, 41.66; HPLC: 96.69%; LCMS (ES, *m*/*z*) calculated for C_22_H_23_BrClN_4_O [(M + H)^+^] 473.07, observed 473.00.

##### 12.1.1.13 (*R*)-1-(4-(6-(1*H*-Pyrazol-4-yl)isoquinolin-1-yl)piperazin-1-yl)-2-amino-3-(3-chlorophenyl) propan-1-one (BHC-13)

Compound BHC-13 was obtained as a pale yellow solid, 58 mg (yield: 64%). Melting point: 136−138 °C; ^1^H NMR (400 MHz, DMSO-*d*_6_): δ ppm 13.12 (s, 1H), 8.26 (brs, 2H), 8.10–8.04 (m, 3H), 7.88 (dd, *J* = 8.4, 1.6 Hz, 1H), 7.35–7.21 (m, 5H), 3.97 (t, *J* = 6.8 Hz, 1H), 3.74–3.70 (m, 3H), 3.64–3.61 (m, 1H), 3.34–3.27 (m, 2H), 3.21–3.17 (m, 1H), 3,08–3.05 (m, 1H), 2.86–2.81 (m, 1H), 2.70–2.65 (m, 1H), 1.76 (brs, 2H); ^13^C NMR (101 MHz, DMSO-*d*_6_): δ 173.49, 160.71, 141.73, 141.27, 138.83, 134.92, 133.13, 130.28, 129.72, 128.74, 126.56, 126.19, 125.28, 121.97, 120.87, 119.50, 116.21, 51.86, 51.49, 51.40, 45.21; HPLC: 95.80%; CMS (ES, *m*/*z*) calculated for C_25_H_26_ClN_6_O [(M + H)^+^] 461.19, observed 461.20.

##### 12.1.1.14 (*R*)-1-(4-(6-(1*H*-Pyrazol-5-yl)isoquinolin-1-yl)piperazin-1-yl)-2-amino-3-(3-chlorophenyl) propan-1-one (BHC-14)

Compound BHC-14 was obtained as a pale yellow solid, 33 mg (yield: 40%). Melting point: 125−127 °C; ^1^H NMR (400 MHz, DMSO-*d*_6_): δ ppm 13.08 (s, 1H), 8.29 (s, 1H), 8.14–8.10 (m, 3H), 7.85 (brs, 1H), 7.43 (d, *J* = 7.0 Hz, 1H), 7.35–7.22 (m, 4H), 6.92 (d, *J* = 2.4 Hz, 1H), 4.02 (t, *J* = 6.8 Hz, 1H), 3.80–3.71 (m, 3H), 3.65–3.58 (m, 1H), 3.28–3.17 (m, 3H), 3.09–3.04 (m, 1H), 2.87–2.82 (m, 1H), 2.72–2.65 (m, 1H), 2.21 (brs, 2H); ^13^C NMR (101 MHz, DMSO-*d*_6_): δ 173.32, 160.74, 141.60, 141.35, 138.59, 133.15, 130.31, 129.74, 128.76, 126.61, 126.15, 124.72, 122.93, 120.37, 116.56, 51.81, 51.50, 51.38, 45.22; HPLC: 90.09%; LCMS (ES, *m*/*z*) calculated for C_25_H_26_ClN_6_O [(M + H)^+^] 461.19, observed 461.30.

##### 12.1.1.15 (*R*)-2-Amino-3-(3-chlorophenyl)-1-(4-(1-methyl-[1,2,4]triazolo[4,3-*a*]quinolin-8-yl) piperazin-1-yl)propan-1-one (BHC-15)

Compound BHC-15 was obtained as a pale yellow solid, 44 mg (yield: 46%). Melting point: 132−135 °C; ^1^H NMR (400 MHz, DMSO-*d*_6_): δ ppm 7.80 (d, *J* = 8.8 Hz, 1H), 7.58–7.55 (m, 2H), 7.34–7.20 (m, 6H), 4.00 (t, *J* = 6.8 Hz, 1H), 3.70–3.52 (m, 4H), 3.43–3.38 (m, 2H), 3.26–3.22 (m, 1H), 3.12 (s, 3H), 3.00–2.98 (m, 1H), 2.85–2.81 (m, 1H), 2.72–2.61 (m, 1H), 1.78 (brs, 2H); ^13^C NMR (101 MHz, DMSO-*d*_6_): δ 173.46, 151.54, 151.08, 146.20, 141.65, 133.83, 133.16, 130.34, 130.30, 129.71, 129.52, 128.75, 126.58, 116.28, 114.78, 110.95, 101.05, 51.80, 47.87, 47.82, 44.64, 41.88, 41.48; HPLC: 97.64%; LCMS (ES, *m*/*z*) calculated for C_24_H_26_ClN_6_O [(M + H)^+^] 449.19, observed 449.03.

##### 12.1.1.16 (*R*)-2-Amino-3-(2,4-dichlorophenyl)-1-(4-(1-methyl-[1,2,4]triazolo[4,3-*a*]quinolin-8-yl) piperazin-1-yl)propan-1-one (BHC-16)

Compound BHC-16 was obtained as a pale yellow solid, 21 mg (yield: 28%). Melting point: 132−135 °C; ^1^H NMR (400 MHz, DMSO-*d*_6_): δ ppm 7.79 (d, *J* = 8.8 Hz, 1H), 7.57–7.55 (m, 3H), 7.40–7.31 (m, 3H), 7.26–7.22 (m, 1H), 4.03 (t, *J* = 7.2 Hz, 1H), 3.71–3.66 (m, 3H), 3.52–3.48 (m, 1H), 3.28–3.22 (m, 3H), 3.11 (s, 3H), 3.09–3.06 (m, 1H), 2.94–2.89 (m, 1H), 2.84–2.79 (m, 1H), 2.02 (brs, 2H); ^13^C NMR (101 MHz, DMSO-*d*_6_): δ 173.26, 151.49, 150.07, 146.20, 135.61, 134.73, 134.08, 133.81, 132.28, 130.30, 129.51, 128.89, 127.48, 116.30, 114.73, 110.95, 101.08, 50.01, 47.92, 47.76, 44.69, 41.55; HPLC: 97.83%; LCMS (ES, *m*/*z*) calculated for C_24_H_25_Cl_2_N_6_O [(M + H)^+^] 483.15, observed 482.95.

##### 12.1.1.17 (*R*)-2-Amino-3-(3-chlorophenyl)-1-(4-(3-methyl-[1,2,4]triazolo[3,4-*a*]isoquinolin-8-yl) piperazin-1-yl)propan-1-one (BHC-17)

Compound BHC-17 was obtained as 37 mg (yield: 28%) of a pale yellow solid. Melting point: 152−154 °C; ^1^H NMR (400 MHz, DMSO-*d*_6_): δ ppm 8.29 (d, *J* = 8.8 Hz, 1H), 8.06 (d, *J* = 7.2 Hz, 1H), 7.40–7.37 (m, 1H), 7.32–7.28 (m, 3H), 7.24–7.21 (m, 2H), 7.14 (d, *J* = 7.6 Hz, 1H), 4.01 (t, *J* = 6.8 Hz, 1H), 3.68–3.61 (m, 3H), 3.53–3.49 (m, 1H), 3.32–3.27 (m, 1H), 3.25–3.21 (m, 2H), 3.02–2.98 (m, 1H), 2.85–2.80 (m, 1H), 2.72–2.68 (m, 1H), 2.66 (s, 3H); ^13^C NMR (101 MHz, DMSO-*d*_6_): δ 173.34, 151.77, 148.05, 145.14, 141.61, 133.15, 131.61, 130.33, 129.72, 128.75, 126.59, 124.31, 121.33, 118.14, 114.35, 113.03, 111.18, 51.79, 48.07, 47.78, 44.74, 41.71, 41.49; HPLC: 98.36%; LCMS (ES, *m*/*z*) calculated for C_24_H_26_ClN_6_O [(M + H)^+^] 449.19, observed 449.24.

##### 12.1.1.18 (*R*)-2-Amino-3-(2,4-dichlorophenyl)-1-(4-(3-methyl-[1,2,4]triazolo[3,4-*a*]isoquinolin-8-yl)piperazin-1-yl)propan-1-one (BHC-18)

Compound BHC-18 was obtained as 39 mg (yield: 34%) of a pale yellow solid. Melting point: 146−148 °C; ^1^H NMR (400 MHz, DMSO-*d*_6_): δ ppm 8.29 (d, *J* = 9.2 Hz, 1H), 8.06 (d, *J* = 7.6 Hz, 1H), 7.58 (d, *J* = 1.6 Hz, 1H), 7.41–7.33 (m, 3H), 7.30–7.29 (m, 1H), 7.14 (d, *J* = 7.6 Hz, 1H), 4.05–4.03 (m, 1H), 3.69–3.60 (m, 3H), 3.52–3.48 (m, 1H), 3.40–3.38 (m, 2H), 3.00–2.89 (m, 3H), 2.84–2.79 (m, 1H), 2.66 (s, 3H); ^13^C NMR (101 MHz, DMSO-*d*_6_): δ 173.14, 151.77, 148.05, 145.14, 135.55, 134.71, 134.14, 132.34, 132.27, 131.61, 128.94, 128.86, 127.49, 124.29, 121.32, 118.22, 114.35, 13.08, 111.25, 49.93, 48.06, 47.85, 44.80, 41.59; HPLC: 91.44%; LCMS (ES, *m*/*z*) calculated for C_24_H_25_Cl_2_N_6_O [(M + H)^+^] 483.15, observed 483.14.

##### 12.1.1.19 (*R*)-2-Amino-3-(3-chlorophenyl)-1-(4-(2-(2-methyl-1H-imidazole-1-yl)quinolin-6-yl) piperazin-1-yl)propan-1-one (BHC-19)

Compound BHC-19 was obtained as a 49 mg (yield: 45%) pale yellow solid. Melting point: 136−138 °C; ^1^H NMR (400 MHz, DMSO-*d*_6_): δ ppm 8.36 (d, *J* = 8.8 Hz, 1H), 7.85 (d, *J* = 9.2 Hz, 1H), 7.69–7.64 (m, 3H), 7.33–7.21 (m, 5H), 6.95 (s, 1H), 4.02 (t, *J* = 6.8 Hz, 1H), 3.68–3.52 (m, 5H), 3.18–3.13 (m, 2H), 2.95–2.88 (m, 1H), 2.85–2.81 (m, 1H), 2.72–2.68 (m, 1H), 2.58 (s, 3H); ^13^C NMR (101 MHz, DMSO-*d*_6_): δ 173.27, 149.37, 147.32, 144.63, 141.59, 141.57, 138.53, 133.17, 130.35, 129.72, 129.36, 128.75, 128.20, 127.78, 126.61, 123.70, 119.88, 116.77, 109.26, 51.75, 48.85, 48.55, 44.85, 41.73, 41.53, 15.93; HPLC: 90.40%; LCMS (ES, *m*/*z*) calculated for C_26_H_28_ClN_6_O [(M + H)^+^] 475.20, observed 475.05.

##### 12.1.1.20 (R)-2-Amino-3-(2,4-dichlorophenyl)-1-(4-(2-(2-methyl-1H-imidazole-1-yl)quinolin-6-yl) piperazin-1-yl)propan-1-one (BHC-20)

Compound BHC-20 was obtained as a pale yellow solid, 56 mg (yield: 55%). Melting point: 142−144 °C; ^1^H NMR (400 MHz, DMSO-*d*_6_): δ ppm 8.36 (d, *J* = 8.4 Hz, 1H), 7.85 (d, *J* = 9.2 Hz, 1H), 7.69–7.60 (m, 4H), 7.38–7.36 (m, 2H), 7.27 (d, *J* = 2.4 Hz, 1H), 6.95 (s, 1H), 4.16 (t, *J* = 6.8 Hz, 1H), 3.72–3.59 (m, 5H), 3.49–3.45 (m, 2H), 3.14–3.12 (m, 1H), 2.98–2.85 (m, 2H), 2.58 (s, 3H); ^13^C NMR (101 MHz, DMSO-*d*_6_): δ 172.18, 149.32, 147.34, 144.64, 141.64, 138.54, 134.97, 134.80, 134.16, 132.59, 129.36, 129.05, 128.19, 127.77, 127.63, 123.78, 119.88, 116.78, 109.36, 49.68, 48.74, 48.61, 44.94, 41.69, 15.92; HPLC: 96.60%; LCMS (ES, *m*/*z*) calculated for C_26_H_27_Cl_2_N_6_O [(M + H)^+^] 509.16, observed 509.05.

##### 12.1.1.21 (*R*)-4-(2-Amino-3-(3-chlorophenyl)propanoyl)-1-(1-methyl-[1,2,4]triazolo[4,3-*a*]quinolin-7-yl)piperazin-2-one (BHC-21)

Compound BHC-21 was obtained as 51 mg (yield: 47%) of a pale yellow solid. Melting point: 186−188 °C; ^1^H NMR (400 MHz, DMSO-*d*_6_): δ ppm 8.39 (d, *J* = 9.2 Hz, 1H), 7.98 (s, 1H), 7.75–7.65 (m, 3H), 7.38–7.25 (m, 4H), 4.45–4.41 (m, 1H), 4.07–3.97 (m, 2H), 3.89–3.78 (m, 4H), 3.08 (s, 3H), 2.91–2.84 (m, 1H), 2.81–2.71 (m, 1H); ^13^C NMR (101 MHz, DMSO-*d*_6_): δ 165.78, 149.50, 146.96, 141.35, 139.53, 133.20, 130.40, 129.76, 129.40, 128.80, 127.49, 126.80, 125.79, 125.58, 125.01, 117.53, 115.64, 52.13, 49.41, 46.82, 42.69, 15.67; HPLC: 90.69%; LCMS (ES, *m*/*z*) calculated for C_24_H_24_ClN_6_O_2_ [(M + H)^+^] 463.16, observed 463.23.

##### 12.1.1.22 (R)-4-(2-Amino-3-(2,4-dichlorophenyl)propanoyl)-1-(1-methyl-[1,2,4]triazolo[4,3-*a*] quinolin-7-yl)piperazin-2-one (BHC-22)

Compound BHC-22 was obtained as a pale yellow solid, 19 mg (yield: 22%). Melting point: 136−138 °C; ^1^H NMR (400 MHz, DMSO-*d*_6_): δ ppm 8.38 (d, *J* = 9.2 Hz, 1H), 7.97 (s, 1H), 7.75–7.66 (m, 3H), 7.59 (s, 1H), 7.44–7.34 (m, 2H), 4.30–4.18 (m, 1H), 4.11–3.97 (m, 2H), 3.92–3.82 (m, 1H), 3.81–3.72 (m, 2H), 3.65–3.62 (m, 1H), 3.08 (s, 3H), 3.00–2.92 (m, 1H), 2.91–2.84 (m, 1H); ^13^C NMR (101 MHz, DMSO-*d*_6_): δ 165.72, 149.51, 146.96, 129.42, 128.95, 127.54, 125.77, 125.04, 117.53, 115.63, 50.50, 42.73, 15.66; HPLC: 91.97%; LCMS (ES, *m*/*z*) calculated for C_24_H_23_Cl_2_N_6_O_2_ [(M + H)^+^] 497.13, observed 497.24.

##### 12.1.1.23 (2*R*)-2-Amino-3-(3-chlorophenyl)-1-(8-(1-methyl-[1,2,4]triazolo[4,3-*a*]quinolin-7-yl)-3,8-diazabicyclo[3.2.1]octan-3-yl)propan-1-one (BHC-23)

Compound BHC-23 was obtained as a pale yellow solid, 24 mg (yield: 22%). Melting point: 139−141 °C; ^1^H NMR (400 MHz, DMSO-*d*_6_): δ ppm 8.16 (d, *J* = 9.6 Hz, 1H), 7.60–7.52 (m, 2H), 7.36–7.33 (m, 3H), 7.32–7.28 (m, 2H), 7.12–6.96 (m, 1H), 4.72–4.52 (m, 1H), 4.48–4.28 (m, 1H), 4.01–3.88 (m, 1H), 3.64–3.58 (m, 1H), 3.46–3.42 (m, 1H), 3.03–2.98 (m, 4H), 2.81–2.61 (m, 3H), 1.99–1.92 (m, 1H), 1.82–1.52 (m, 3H): ^13^C NMR (101 MHz, DMSO-*d*_6_): δ 149.20, 148.86, 148.75, 146.12, 141.18, 140.95, 133.23, 130.45, 130.37, 129.82, 129.60, 128.80, 128.70, 126.75, 125.60, 125.49, 124.94, 117.38, 117.24, 117.04, 116.97, 115.01, 112.61, 54.59, 54.11, 54.03, 53.82, 53.58, 53.20, 52.91, 51.12, 50.96, 41.55, 28.79, 27.96, 26.84, 26.49, 15.49; HPLC: 89.28%; LCMS (ES, *m*/*z*) calculated for C_26_H_28_ClN_6_O [(M + H)^+^] 475.20, observed 475.22.

##### 12.1.1.24 (2*R*)-*A*-Amino-3-(2,4-dichlorophenyl)-1-(8-(1-methyl-[1,2,4]triazolo[4,3-*a*]quinolin-7-yl)-3,8-diazabicyclo[3.2.1]octan-3-yl)propan-1-one (BHC-24)

Compound BHC-24 was obtained as a pale yellow solid, 32 mg (yield: 28%). Melting point: 158−160 °C; ^1^H NMR (400 MHz, DMSO-*d*_6_): δ ppm 8.17–8.14 (m, 1H), 7.62–7.48 (m, 3H), 7.41–7.16 (m, 4H), 4.72–4.38 (m, 2H), 3.92–3.82 (m, 1H), 3.68–3.48 (m, 2H), 3.02–2.94 (m, 5H), 2.86–2.82 (m, 1H), 2.62–2.58 (m, 1H), 2.02–1.92 (m, 1H), 1.82–1.56 (m, 5H): ^13^C NMR (101 MHz, DMSO-*d*_6_): δ 170.81, 149.22, 148.86, 146.10, 135.64, 134.73, 134.65, 134.18, 134.02, 132.31, 129.97, 129.82, 128.91, 127.51, 125.63, 117.31, 117.16, 116.98, 115.02, 114.89, 112.62, 54.56, 54.31, 54.20, 53.96, 53.68, 53.19, 51.54, 51.37, 51.15, 50.98, 28.83, 26.82, 26.43, 15.49; HPLC: 90.50%; LCMS (ES, *m*/*z*) calculated for C_26_H_27_Cl_2_N_6_O [(M + H)^+^] 509.16, observed 509.17.

##### 12.1.1.25 (2*R*)-2-Amino-3-(3-chlorophenyl)-1-(3-(1-methyl-[1,2,4]triazolo[4,3-*a*]quinolin-7-yl)-3,8-diazabicyclo[3.2.1]octan-8-yl)propan-1-one (BHC-25)

Compound BHC-25 was obtained as a pale yellow solid, 22 mg (yield: 25%). Melting point: 127−129 °C; ^1^H NMR (400 MHz, DMSO-*d*_6_): δ ppm 8.17 (dd, *J* = 9.6, 2.8 Hz, 1H), 7.57–7.54 (m, 2H), 7.42–7.38 (m, 1H), 7.35–7.31 (m, 2H), 7.23–7.15 (m, 2H), 7.11–7.03 (m, 1H), 4.44–4.35 (m, 2H), 3.51–3.42 (m, 1H), 3.06–2.92 (m, 4H), 2.89–2.84 (m, 1H), 2.74–2.68 (m, 1H), 2.57–2.53 (m, 1H), 1.98–1.92 (m, 4H), 1.82–1.62 (m, 1H), 1.42–1.12 (m, 1H); ^13^C NMR (101 MHz, DMSO-*d*_*6*_): δ 175.57, 175.19, 149.18, 146.08, 144.43, 141.80, 141.51, 133.23, 132.97, 130.37, 130.06, 129.67, 129.51, 128.64, 128.59, 126.60, 126.38, 126.19, 124.38, 118.37, 117.91, 115.10, 115.03, 114.14, 54.41, 54.36, 54.06, 54.00, 52.09, 51.93, 47.14, 46.82, 44.38, 44.01, 41.47, 27.08, 26.72, 15.51; HPLC: 99.40%; LCMS (ES, *m*/*z*) calculated for C_26_H_28_ClN_6_O [(M + H)^+^] 475.20, observed 475.21.

##### 12.1.1.26 (2*R*)-2-Amino-3-(2,4-dichlorophenyl)-1-(3-(1-methyl-[1,2,4]triazolo[4,3-*a*]quinolin-7-yl)-3,8-diazabicyclo[3.2.1]octan-8-yl)propan-1-one (BHC-26)

Compound BHC-26 was obtained as a 56 mg (yield: 39%) pale yellow solid. Melting point: 166−168 °C; ^1^H NMR (400 MHz, DMSO-d6): δ ppm 8.17 (d, *J* = 9.2 Hz, 1H), 7.61–7.53 (m, 3H), 7.44–7.36 (m, 2H), 7.33–7.28 (m, 1H), 7.26–7.18 (m, 1H), 4.48–4.42 (m, 2H), 3,98–3.82 (m, 1H), 3.71–3.68 (m, 2H), 3.48–3.46 (m, 1H), 3.06–2.92 (m, 5H), 2.82–2.68 (m, 2H), 1.98–1.64 (m, 5H): ^13^C NMR (101 MHz, DMSO-d6): δ 149.19, 146.10, 144.40, 135.83, 135.53, 134.59, 134.07, 133.73, 129.66, 129.56, 128.90, 128.68, 127.52, 127.19, 126.18, 124.42, 118.38, 118.31, 117.89, 115.10, 115.04, 114.15, 114.10, 54.38, 54.11, 53.90, 50.19, 50.05, 46.84, 44.47, 44.05, 27.08, 26.71, 15.49; HPLC: 95.04%; LCMS (ES, *m*/*z*) calculated for C_26_H_27_Cl_2_N_6_O [(M + H)^+^] 509.16, observed 509.14.

### 12.2 Biochemical enzyme activity assays

HDAC4 and 8 were produced in E.coli using previously published procedures.^46,47^ HDAC10 and HDAC11 were produced similar to HDAC8 with the following changes: the genes were synthesized, codon-optimized, and subsequently cloned into a pET14b vector, in-frame with either a His_6_-MBP tag or a His_6_-SUMO tag. Protein expression was performed in LB medium and induced with 0.2 mM IPTG at an OD_600_ of 0.6–0.8. Cultures were then incubated overnight at 16 °C. Both proteins were purified *via* immobilized metal affinity chromatography (IMAC), using a Ni-IDA column for HDAC11 and a complete™ His-Tag Purification Column for hHDAC10. Following purification, the proteins were buffer-exchanged into SEC buffer, and the volume was concentrated by ultrafiltration. HDAC1 and 6 were purchased from BPS Bioscience (USA). For HDAC4 and HDAC8, a serial dilution of inhibitor in assay buffer (25 mM Tris–HCl, pH 8.0, 75 mM KCl, 0.00001% Pluronic F-127) was incubated with 10 nM HDAC8 or 1 nM in a black 96-well microplate (Greiner, Germany) for 60 min at 30 °C. The reaction was initiated by the addition of substrate (20 µM Boc-Lys(trifluoroacetyl)-7-amino-4-methylcoumarin) (Bachem, Switzerland). After incubation for 60 min at 30 °C, the reaction was stopped by the addition of 1.7 µM 9,9,9-trifluoro-8-oxo-*N*-phenylnonanamide and the deacetylated substrate was converted into a fluorescent product by the addition of 0.4 mg mL^−1^ trypsin (Roth, Germany). The release of 7-amino-4-methylcoumarin was monitored in a microplate reader at 450 nm (λEx = 350 nm) and correlated with enzyme activity. Dose–response curves were generated using GraphPad Prism and fitted to a four-parameter logistic function to obtain IC_50_ values:
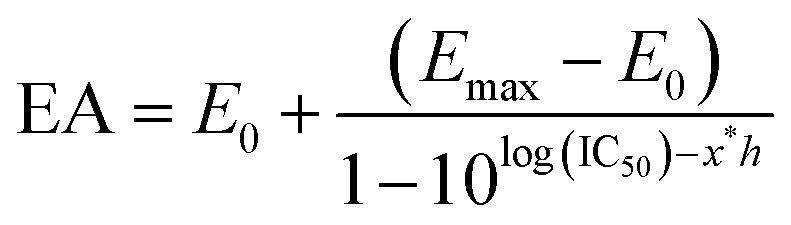
where EA is the enzyme activity at a given inhibitor concentration *x*. *E*_max_ and *E*_0_ are the enzyme activities in the absence of ligand and under complete inhibition, respectively. IC_50_ is the ligand concentration at which half of the enzyme activity is inhibited. The parameter *h* is a measure of the steepness of the dose-response curve.

The assays for the other isoenzymes deviated slightly from this instruction: the assays for HDAC1 and HDAC6 reacted with 50 µM Boc-Lys(acetyl)-7-amino-4-methylcoumarin as substrate and were stopped with 4.3 µM SAHA. For HDAC10, 100 nM enzyme was used and the enzyme reaction was stopped with 3.8 µM quisinostat. For HDAC11, 100 nM enzyme and 80 µM Boc-Lys(trifluoroacetyl)-7-amino-4-methylcoumarin substrate were used and the reaction was stopped with 3.8 µM FT895. In addition, MAL buffer (137 mmol L^−1^ NaCl, 50 mmol L^−1^ Tris–HCl, 2.7 mmol L^−1^ KCl, 1 mmol L^−1^ MgCl_2_, 0.5 mg mL^−1^ bovine serum albumin (BSA), pH 8.0) was used instead of the assay buffer mentioned above.

### 12.3 Cell culture and treatment

Human cancer cell line IMR-32 and HEK293T were cultured in Dulbecco's DMEM (Invitrogen), FaDu cells were maintained in EMEM (Invitrogen), and SH-SY5Y cells were cultured in DMEM/F12 medium (Invitrogen). All media were supplemented with 10% FBS, 2 mM glutamine, and 1× penicillin–streptomycin (Gibco). The cells were maintained at 37 °C in a humidified atmosphere containing 5% CO_2_. Cells were treated with the respective compounds at the concentrations and durations indicated in the figures and corresponding legends.

### 12.4 Cell proliferation assay – IC_50_ experiments

Cell proliferation was assessed using the WST-1 reagent (Cat# 05 015 944 001, Roche) following the manufacturer's instructions at the indicated time points.^41^ Approximately 6000–12000 cells were seeded per well in 96-well plates and treated with test compounds at various concentrations (5, 10, 20, 40, 80, 160, and 320 µM). After 24 hours of incubation at 37 °C, WST-1 reagent was added to each well, and absorbance was measured at 450 nm using a microplate reader. The half-maximal inhibitory concentration (IC_50_) values were calculated using non-linear regression analysis in GraphPad Prism version 5.^41^

### 12.5 Colony formation assay

Colony formation assay was performed as described previously.^41^ Around 12 000 IMR-32 cells were seeded per well in a 96-well plate for colony formation assay and treated with vehicle control, BHC-2, BHC-10, and BHC-13 compounds at different concentrations. After 24 hours of treatment, cells were trypsinized and re-seeded at a number of 250 cells per well in a 6-well plate and incubated at 37 °C for another 14 days. The media was replaced every three days. Colonies formed after 14 days were fixed with 4% paraformaldehyde, stained with crystal violet solution, and counted as described previously.^41^

### 12.6 Annexin V-FITC-based flow cytometry

For flow cytometric analysis, IMR-32 cells were treated with DMSO (vehicle control), BHC-2, BHC-10, or BHC-13 for 24 hours. After treatment, cells were collected, washed, and stained with Annexin V-FITC and propidium iodide using the Annexin V-FITC Apoptosis Detection Kit (Invitrogen, Cat# V13242), following the manufacturer's protocol. Samples were analyzed using a BD FACS Aria III flow cytometer, and data were processed with FlowJo V10 software to determine the percentages of early and late apoptotic cells. All experiments were conducted in triplicate and repeated independently to ensure reproducibility.

### 12.7 Caspase activity assay

Caspase-3/7 activity was measured using the Caspase-Glo® 3/7 Assay Kit (Promega, Cat# G8090). IMR-32 cells were treated with BHC-2, BHC-10, or BHC-13 compounds for 24 hours. Following treatment, Caspase-Glo® reagent containing the DEVD-luciferin substrate and lysis buffer was added to each well as per the manufacturer's instructions.^41^ Luminescence was recorded using a microplate reader, and results were normalized to vehicle-treated controls to determine relative caspase activity.

### 12.8 Flow cytometric analysis of DNA content

To assess the effects of anticancer compounds BHC-2, BHC-10, and BHC-13 on cell cycle progression, DNA content analysis was performed in IMR-32 cells using flow cytometry, as previously described.^32,42^ Briefly, control and treated cells were harvested, fixed in 70% ethanol, and stored at −20 °C. Fixed cells were subsequently washed with PBS and incubated with propidium iodide (PI) staining solution (Invitrogen) for 20 minutes at room temperature in the dark.

Approximately 1 × 10^6^ cells per sample were analyzed using a BD FACS Aria III flow cytometer. The distribution of cells across G_1_, S, and G_2_/M phases was determined using FlowJo V10 software. All experiments were performed in biological triplicate.

### 12.9 Western blotting

Western blotting was performed as described previously.^32^ Briefly, IMR-32 neuroblastoma cells treated for 24 hours with or without the BHC-2, BHC-10, and BHC-13 compounds were washed with ice-cold 1× PBS and lysed in lysis buffer containing 1% Triton X-100, 10 mM Tris base, 150 mM NaCl, 1 mM EDTA, 0.2 mM EGTA, and 0.5% IGEPAL (pH 8.0). Protein concentrations were determined using the BCA protein assay, and equal amounts of protein were separated by SDS-PAGE and transferred onto PVDF membranes. The membranes were blocked with 5% skimmed milk for 2 hours and incubated overnight at 4 °C with either anti-acetyl SMC3 antibody (1 : 1000; Sigma, Cat# MABE1073) or total SMC3 antibody (1 : 2000; Abclonal, Cat# A18402). After washing, the membranes were incubated with HRP-conjugated anti-mouse IgG secondary antibody (1 : 20 000; Invitrogen, Cat# A16072) for 2 hours at room temperature. Blots were stripped and re-probed with β-actin antibody (1 : 100 000; Sigma, Cat# A1978) as a loading control. Densitometric quantification of acetylated SMC3 was performed using ImageJ software (NIH, Bethesda, MD), and values were normalized to total SMC3 levels.

### 12.10 Molecular docking, MMGBSA and molecular dynamics simulations

#### 12.10.1 Protein preparation and grid generation

For molecular docking studies, the protein (PDB: 3SFH)^36^ was downloaded from protein data bank (https://www.rcsb.org/) and imported into the maestro interface of the Schrödinger software (Schrödinger, LLC., NY, v2022).^48^ To prepare the protein, protein preparation wizard was executed during which missing residues were added using prime module, and water molecules in the radius of 5.0 Å around the ligand were removed. Further, to prepare the grid for carrying out molecular docking studies, receptor-grid generation wizard was used, and the co-ordinates of the co-crystallized ligand (*X* = 9.26; *Y* = 13.73 and *Z* = −23.86) were selected to generate a grid box of 10 Å.^49^

#### 12.10.2 Ligand preparation

The molecules BHC-2, BHC-10, and BHC-13 were sketched in the 2D sketcher of the Maestro interface and prepared using the Ligprep module of the Schrödinger software (Schrödinger, LLC, NY, v2022).^49^ The forcefield used was optimized potential for liquid simulations 2005 (OPLS_2005), and the ionization sate was set to neutralize. The chiralities were determined from the 3D structure. These prepared ligands were used to carry out molecular docking study.

#### 12.10.3 Molecular docking

Molecular docking helps determine the binding pattern of molecules within the active site of a protein. The ligand docking wizard, which utilises the Glide module of the Schrödinger software, was used to carry out this analysis.^50^ The mode of precision was set to extra precision (XP) and the ligand sampling was flexible to generate several poses and produce the best among them as output. All other parameters were set to default.

#### 12.10.4 MMGBSA studies

Molecular Mechanics- Generalized Born surface Area (MM-GBSA) solvation helps to determine the free energy (Δ*G*) of the ligand by including van der Waals, bonded and electrostatic interactions.^51^ The complexes from molecular docking were considered for MM-GBSA studies using prime module of the Schrödinger software. The solvation model used was Vacuum Solvent Generalized Born (VSGB) continuum and the forcefield was OPLS_2005.

#### 12.10.5 Molecular dynamics analysis

The complexes from molecular docking were imported into the Desmond module of the Schrödinger software, the system was setup using transferable intermolecular potential with 3 points (TIP3P) solvent model^52^ and the boundary conditions were set to cubic box with dimension of 10 Å. Salt concentration of 0.15 M NaCl is added to mimic the biological fluid. The complete solvated system is then used to carry out molecular dynamics study for 100 ns with recording interval of 100 pc at temperature of 310 K and 1.012 bar pressure using NTP ensembles. To maintain the constant temperature and pressure Nose–Hoover chain thermostat and Martyna-Tobias-Klein barostat were used respectively.^53,54^ For comparative analysis, the protein in complex with Compound A was also subjected to MD analysis. The root mean square deviations (RMSD) and fluctuations (RMSF) were used to interpret the stability of the complexes. The trajectories from the molecular dynamics were used to carry out principal component analysis (PCA) that captures the variance of changes in the conformations of the protein-ligand complex during the MD simulation. To perform this, trajectories from MD simulations were used to generate a *.dcd file using visual molecular dynamics (VMD) software, and the same was subjected to Galaxy Europe web-server to perform principal component analysis using a Bio3D package.^55,56^ This analysis helps in understanding the behaviour of the complexes during the simulations.

## Author contributions

NVM, ACS, and KVGCS conceptualized the chemistry, data curation, methodology, writing – original draft, and formal analysis. KSC contributed to the data curation, writing and editing of the manuscript. MS and FJMA conceptualized the HDAC enzymatic inhibition assays and contributed to the writing and editing of the manuscript. MRM contributed to the docking analysics and editing of the manuscript. VJ and VS conceptualized the cell biology assays and contributed to the writing and editing of the manuscript.

## Conflicts of interest

There are no conflicts of interest to declare.

## Supplementary Material

RA-OLF-D6RA06186A-s001

## Data Availability

The data supporting this article have been included as part of the supplementary information (SI). Supplementary information: experimental details for the preparation of intermediates, analytical data of final compounds BHC-1 to BHC-26, dose-response curves against HDAC-isoenzymes. See DOI: https://doi.org/10.1039/d6ra06186a.
