# Supplementary material for: Exploration of non-hydroxamate based quinoline analogues as HDAC8 inhibitors
Source: RSC Adv. 2026 Aug 18;16(41):48628–49. doi: 10.1039/d6ra06186a (PMC13482965; doi:10.1039/d6ra06186a)
Supplement: Supplementary file 1 [file RA-016-D6RA06186A-s001.pdf]

Supplementary Information (SI) for RSC Advances.
This journal is © The Royal Society of Chemistry 2026

Exploration of Non-Hydroxamate Based Quinoline Analogues as HDAC8 Inhibitors

N.V.M. Rao Bandaru[a,c], Vandana Joshi[b], Markus Schweipert[d,e], Kosana Sai Chaitanya[a], Dharani Sen[b], Muzaffar-Ur-Rehman Mohammed,[f] Vivek Sharma[b], Chandrasekhar Abbineni[c], Franz-Josef Meyer-Almes*[d,e], Kondapalli Venkata Gowri Chandra Sekhar*[a]

[a]Department of Chemistry, Birla Institute of Technology and Science, Pilani, Hyderabad Campus, Jawahar Nagar, Hyderabad 500 078, Telangana, India

[b]Department of Biological Sciences, Birla Institute of Technology and Science, Pilani, Hyderabad Campus, Jawahar Nagar, Hyderabad 500 078, Telangana, India

[c]Aurigene Oncology Limited, 39-40 KIADB Industrial Area Electronic City Phase II, Hosur Road, Bangalore 560 100, India

[d]Department of Chemical Engineering and Biotechnology, University of Applied Sciences Darmstadt, Haardtring 100, 64295 Darmstadt, Germany.

[e]European University of Technology, European Union

[f]Department of Pharmacy, Birla Institute of Technology and Science, Pilani Campus, Pilani 333031, Rajasthan, India

*Email: franz-josef.meyer-almes@h-da.de, kvgc@hyderabad.bits-pilani.ac.in

List of contents:

1.	Prepration of intermediates	S2-S15
2.	Analytical data of final compounds **BHC-1** to **BHC-26**	S16-S77
3	Dose-response curves against HDAC-isoenzymes.	S78
4	Uncropped images of Western Blot analysis	S79-S80

Synthesis of intermediates:

General procedure A for SNAr coupling:

To the stirred solution of corresponding halo compound (1 eq) in DMF (5 vol) were added amine compound (1.1 eq) and Cs_2CO_3.(3 eq). Then stirred the reaction mixture at 90 ^{0}C for 16h. Reaction was monitored by TLC. After completion of the reaction, the reaction mixture was diluted with water (3 x 5 vol) and extracted with EtOAc (6 x 5 vol). Dried the organic layer over Na_2SO_4. Concentrated the organic layer under reduced pressure. The resultant crude was purified by using combi flash to obtain the product (Yield: 80 to 95%).

General procedure B for Boc deprotection:

To the stirred solution of corresponding Boc compound (1 eq) in DCM (10 vol) was added TFA (10 eq) at 0 ^{0}C. Then stirred the reaction mixture at RT for 4h. Reaction was monitored by TLC. After completion of the reaction, the reaction mixture was concentrated under reduced pressure. The resultant crude washed with diethyl ether (2x10 vol), then dried the solid under reduced pressure to obtain the crude product as TFA salt. (Yield: 90 to 98%).

General procedure C for amide coupling:

To the stirred solution of corresponding acid compound (1 eq) in DMF (5 vol) were added DIPEA (4 eq) and HATU (1.3 eq) at ^{0}C and stirred at same temperature for 10 min. Then corresponding amine (1.1 eq) was added and stirred the reaction mixture at RT for 2h. Reaction was monitored by TLC. After completion of the reaction, the reaction mixture was diluted with water (3 x 5 vol) and extracted with EtOAc (6 x 5 vol). Dried the organic layer over Na_2SO_4. Concentrated the organic layer under reduced pressure. The resultant crude was purified by using combi flash to obtain the product (Yield: 75 to 85%).

General procedure D for Suzuki coupling:

A solution of the halo compound (1 eq.), corresponding boronic ester or corresponding acid (1.7 eq) and Cs_2CO_3 (3 eq) in 1,4-dioxane (8 vol) and water (2 vol) was degassed with argon for 10 min, after that Pd(dppf)Cl_2.DCM was added and again degassed for 10 min. Then stirred the reaction mixture at 90 ^{0}C for 16h. Reaction was monitored by TLC. After completion of the reaction, the reaction mixture was diluted with water (3 x 5 vol) and extracted with EtOAc

(6 x 5 vol). Dried the organic layer over Na_2SO_4. Concentrated the organic layer under reduced pressure. The resultant crude was purified by using combi flash to obtain the product (Yield: 65 to 85%).

General procedure E for SNAr with hydrazine hydrate:

The corresponding halo compound was treated with hydrazine hydrate and heated to 100 ^{0}C for 16h. Reaction was monitored by TLC. After completion of the reaction, the reaction mixture was diluted with water (3 x 5 vol) and extracted with EtOAc (6 x 5 vol). Dried the organic layer over Na_2SO_4. Concentrated the organic layer under reduced pressure. The resultant crude was washed with diethyl ether (10 vol) and dried under high vacuum to afford the compound. (Yield: 85 to 95%).

General procedure F for cyclisation with acetic acid:

The corresponding hydrazine compound was treated with acetic acid and heated to 120 ^{0}C for 16h under seal tube condition. Reaction was monitored by TLC. After completion of the reaction, the reaction mixture was concentrated under reduced pressure. The resultant crude was diluted with water (3 x 10 vol) and extracted with EtOAc (6 x 10 vol). Dried the organic layer over Na_2SO_4. Concentrated the organic layer under reduced pressure. Concentrated the organic layer under reduced pressure. The resultant crude was purified by using combi flash to obtain the product (Yield: 68 to 90%).

General procedure G for Buchwald coupling:

A solution of the halo compound (1 eq.), corresponding amine (2 eq) and Cs_2CO_3 (3 eq) in 1,4-dioxane (10 vol) was degassed with argon for 10 min, after that Pd_2dba_3 (0.1 eq) and Xanthphos (0.2 eq) were added and again degassed for 10 min. Then stirred the reaction mixture at 110 ^{0}C for 16h. Reaction was monitored by TLC. After completion of the reaction, the reaction mixture was diluted with water (3 x 5 vol) and extracted with EtOAc (6 x 5 vol). Dried the organic layer over Na_2SO_4. Concentrated the organic layer under reduced pressure. The resultant crude was purified by using combi flash to obtain the product (Yield: 60 to 75%).

***tert*-Butyl 4-(6-bromoquinolin-2-yl)piperazine-1-carboxylate (Int-2):**

Int-2 prepared according to General Procedure A.^{1}H NMR (400 MHz, DMSO-*d*$_6$): δ ppm 8.05 (d, *J* = 9.2 Hz, 1H), 7.97 (s, 1H), 7.62 (dd, *J* = 8.8, 2.4 Hz, 1H), 7.50 (d, *J* = 9.2 Hz, 1H), 7.29 (d, *J* = 9.6 Hz, 1H), 3.71–3.68 (m, 4H), 3.47–3.44 (m, 4H), 1.44 (s, 9H); LCMS (ES, m/z) calculated for $C_{18}H_{23}BrN_3O_2$ [(M + H)$^+$] 392.10, observed 392.05.

4-(6-Bromoquinolin-2-yl)piperazin-1-ium 2,2,2-trifluoroacetate (Int-3):

Int-3 prepared according to General Procedure B.^{1}H NMR (400 MHz, DMSO-*d*$_6$): δ ppm 9.41 (brs, 2H), 8.23 (d, *J* = 8.8 Hz, 1H), 8.10 (s, 1H), 7.78–7.76 (m, 2H), 7.47 (d, *J* = 8.8 Hz, 1H), 4.08–4.06 (m, 4H), 3.26–3.22 (m, 4H); LCMS (ES, m/z) calculated for $C_{13}H_{15}BrN_3$ [(M + H)$^+$] 292.04, observed 292.16.

***tert*-Butyl (*R*)-(1-(4-(6-bromoquinolin-2-yl)piperazin-1-yl)-3-(3-chlorophenyl)-1-oxopropan-2-yl)carbamate (Int-4):**

Prepared according to General Procedure C.^{1}H NMR (400 MHz, DMSO-*d*$_6$): δ ppm 8.05 (d, *J* = 9.6 Hz, 1H), 7.97 (s, 1H), 7.64 (dd, *J* = 8.8, 2.4 Hz, 1H), 7.51 (d, *J* = 9.2 Hz, 1H), 7.36 (s, 1H), 7.32–7.21 (m, 5H), 4.65–4.62 (m, 1H), 3.72–3.56 (m, 6H), 3.49–3.47 (m, 1H), 2.95–2.92 (m, 1H), 2.82–2.78 (m, 1H), 2.43–2.41 (m, 1H), 1.32 (s, 9H); LCMS (ES, m/z) calculated for $C_{27}H_{31}BrClN_4O_3$ [(M + H)$^+$] 573.13, observed 573.04.

***tert*-Butyl (*R*)-(1-(4-(6-(2-aminophenyl)quinolin-2-yl)piperazin-1-yl)-3-(3-chlorophenyl)-1-oxopropan-2-yl)carbamate (Int-5a):**

Prepared according to General Procedure D. ^{1}H NMR (400 MHz, DMSO-*d*$_6$): δ ppm 8.13 (d, *J* = 9.8 Hz, 1H), 7.76 (d, *J* = 1.6 Hz, 1H), 7.66–7.58 (m, 2H), 7.37 (s, 1H), 7.33–7.22 (m, 5H), 7.08–7.04 (m, 2H), 6.79 (dd, *J* = 8.0, 1.4 Hz, 1H), 6.68–6.64 (m, 1H), 4.81 (s, 2H), 4.68–4.64 (m, 1H), 3.66–3.52 (m, 8H), 2.96–2.93 (m, 1H), 2.84–2.82 (m, 1H), 1.32 (s, 9H); LCMS (ES, m/z) calculated for $C_{33}H_{37}ClN_5O_3$ [(M + H)$^+$] 586.26, observed 586.23.

***tert*-Butyl (*R*)-(1-(4-(6-(1*H*-pyrazol-4-yl)quinolin-2-yl)piperazin-1-yl)-3-(3-chlorophenyl)-1-oxopropan-2-yl)carbamate (Int-5b):**

Prepared according to General Procedure D. ^{1}H NMR (400 MHz, DMSO-*d*$_6$): δ ppm 12.92 (s, 1H), 8.25 (s, 1H), 8.02 (d, *J* = 9.2 Hz, 1H), 7.99 (s, 1H), 7.95 (d, *J* = 2.0 Hz, 1H), 7.83 (dd, *J* = 8.8, 2.0 Hz, 1H), 7.58 (d, *J* = 8.8 Hz, 1H), 7.34 (s, 1H), 7.31–7.23 (m, 5H), 4.64–4.62 (m, 1H),

3.72–3.51 (m, 8H), 2.94–2.92 (m, 1H), 2.84–2.82 (m, 1H), 1.32 (s, 9H); LCMS (ES, m/z) calculated for $C_{30}H_{34}ClN_6O_3$ [(M + H)$^+$] 561.24, observed 561.35.

***tert*-Butyl ((2*R*)-3-(3-chlorophenyl)-1-oxo-1-(4-(6-(1-(tetrahydro-2*H*-pyran-2-yl)-1*H*-pyrazol-5-yl)quinolin-2-yl)piperazin-1-yl)propan-2-yl)carbamate (Int-5c):**

Prepared according to General Procedure D. ^{1}H NMR (400 MHz, DMSO-d_6): δ ppm 8.13 (d, J = 8.2 Hz 1H), 7.86 (s, 1H), 7.68–7.66 (m, 2H), 7.58 (s, 1H), 7.34–7.18 (m, 6H), 6.51 (d, *J* = 1.6 Hz, 1H), 5.24–5.22 (m, 1H), 4.72–4.68 (m, 1H), 3.74–3.52 (m, 10H), 2.94–2.92 (m, 1H), 2.84–2.82 (m, 1H), 198–1.92 (m, 1H), 1.71–1.69 (m, 1H), 1.58–1.52 (m, 4H), 1.32 (s, 9H); LCMS (ES, m/z) calculated for $C_{35}H_{42}ClN_6O_4$ [(M + H)$^+$] 645.31, observed 645.34.

***tert*-Butyl 4-(7-bromoquinolin-2-yl)piperazine-1-carboxylate (Int-7):**

Prepared according to General Procedure A.^{1}H NMR (400 MHz, DMSO-d_6): δ ppm 8.08 (d, *J* = 8.8 Hz, 1H), 7.73 (s, 1H), 7.68 (d, *J* = 8.4 Hz, 1H), 7.36 (dd, *J* = 8.4, 2.0 Hz, 1H), 7.28 (d, *J* = 9.2 Hz, 1H), 3.73–3.70 (m, 4H), 3.47–3.44 (m, 4H), 1.44 (s, 9H); LCMS (ES, m/z) calculated for $C_{18}H_{23}BrN_3O_2$ [(M + H)$^+$] 392.10, observed 392.13.

4-(7-Bromoquinolin-2-yl)piperazin-1-ium 2,2,2-trifluoroacetate (Int-8):

Prepared according to General Procedure B. ^{1}H NMR (400 MHz, DMSO-d_6): δ ppm 9.56 (brs, 2H), 8.34–8.32 (m, 2H), 7.82 (s, 1H), 7.57–7.48 (m, 2H), 4.15–4.13 (m, 4H), 3.29–3.27 (m, 4H); LCMS (ES, m/z) calculated for $C_{13}H_{15}BrN_3$ [(M + H)$^+$] 292.04, observed 291.85

***tert*-Butyl (*R*)-(1-(4-(7-bromoquinolin-2-yl)piperazin-1-yl)-3-(3-chlorophenyl)-1-oxopropan-2-yl)carbamate (Int-9a):**

Prepared according to General Procedure C. ^{1}H NMR (400 MHz, DMSO-d_6): δ ppm 8.08 (d, *J* = 9.2 Hz, 1H), 7.72 (s, 1H), 7.68 (d, *J* = 8.4 Hz, 1H), 7.34–7.32 (m, 2H), 7.28–7.18 (m, 5H), 4.67–4.65 (m, 1H), 3.72–3.52 (m, 8H), 2.94–2.92 (m, 1H), 2.85–2.82 (m, 1H), 1.32 (s, 9H); LCMS (ES, m/z) calculated for $C_{27}H_{31}BrClN_4O$ [(M + H)$^+$] 573.13, observed 573.64.

***tert*-Butyl (*R*)-(1-(4-(7-bromoquinolin-2-yl)piperazin-1-yl)-3-(2,4-dichlorophenyl)-1-oxopropan-2-yl)carbamate (Int-9b):**

Prepared according to General Procedure C. ^1H NMR (400 MHz, DMSO-d_6): δ ppm 8.09 (d, *J* = 9.2 Hz, 1H), 7.75 (d, *J* = 1.6 Hz, 1H), 7.68 (d, *J* = 8.4 Hz, 1H), 7.58 (d, *J* = 2.0 Hz, 1H), 7.42–7.35 (m, 3H), 7.30 (d, *J* = 9.2 Hz, 1H), 7.22 (d, *J* = 8.8 Hz, 1H), 4.78–4.76 (m, 1H), 3.72–3.52 (m, 8H), 3.05–3.02 (m, 1H), 2.91–2.89 (m, 1H), 1.28 (s, 9H); LCMS (ES, m/z) calculated for $C_{27}H_{30}BrCl_2N_4O_3$ [(M + H)$^+$] 607.09, observed 607.14.

***tert*-Butyl(*R*)-(1-(4-(7-(1*H*-pyrazol-4-yl)quinolin-2-yl)piperazin-1-yl)-3-(3-chlorophenyl)-1-oxopropan-2-yl)carbamate (Int-10a):**

Prepared according to General Procedure D. ^1H NMR (400 MHz, DMSO-d_6): δ ppm 12.98 (s, 1H), 8.32 (s, 1H), 8.01-7.98 (m, 2H), 7.79 (s, 1H), 7.69 (d, *J* = 8.0 Hz, 1H), 7.55 (d, *J* = 8.4 Hz, 1H), 7.37 (s, 1H), 7.28–7.18 (m, 4H), 7.14 (d, *J* = 6.8 Hz, 1H), 4.64–4.62 (m, 1H), 3.72–3.52 (m, 8H), 2.84–2.82 (m, 1H), 2.74–2.72 (m, 1H), 1.32 (s, 9H); LCMS (ES, m/z) calculated for $C_{30}H_{34}ClN_6O_3$ [(M + H)$^+$] 561.24, observed 561.27.

***tert*-Butyl((2*R*)-3-(3-chlorophenyl)-1-oxo-1-(4-(7-(1-(tetrahydro-2*H*-pyran-2-yl)-1*H*-pyrazol-5-yl)quinolin-2-yl)piperazin-1-yl)propan-2-yl)carbamate (Int-10b):**

Prepared according to General Procedure D. ^1H NMR (400 MHz, DMSO-d_6): δ ppm 8.16 (d, *J* = 8.8 Hz, 1H), 8.00 (s, 1H), 7.81–7.72 (m, 3H), 7.35–7.18 (m, 6H), 6.86 (d, *J* = 1.2 Hz, 1H), 5.26–5.24 (m, 1H), 4.71–4.69 (m, 1H), 3.72–3.52 (m, 10H), 2.96–2.92 (m, 1H), 2.84–2.82 (m, 1H), 197–1.94 (m, 1H), 1.72–1.68 (m, 1H), 1.56–1.52 (m, 4H), 1.32 (s, 9H); LCMS (ES, m/z); LCMS (ES, m/z) calculated for $C_{35}H_{42}ClN_6O_4$ [(M + H)$^+$] 645.30, observed 645.32.

***tert*-Butyl (*R*)-(1-(4-(7-(1*H*-pyrazol-4-yl)quinolin-2-yl)piperazin-1-yl)-3-(2,4-dichlorophenyl)-1-oxopropan-2-yl)carbamate (Int-10c):**

Prepared according to General Procedure D.^1H NMR (400 MHz, DMSO-d_6): δ ppm13.00 (s, 1H), 8.36 (brs, 1H), 8.06 (brs, 1H), 8.02 (d, *J* = 9.2 Hz, 1H), 7.81 (s, 1H), 7.69 (d, *J* = 8.4 Hz, 1H), 7.59–7.53 (m, 2H), 7.43–7.35 (m, 2H), 7.23–7.16 (m, 2H), 4.75–4.73 (m, 1H), 3.67–3.57 (m, 8H), 3.09–3.04 (m, 1H), 2.93–2.87 (m, 1H), 1.29 (s, 9H); LCMS (ES, m/z) calculated for $C_{30}H_{33}Cl_2N_6O_3$ [(M + H)$^+$] 595.2, observed 595.25.

***tert*-Butyl 4-(isoquinolin-1-yl)piperazine-1-carboxylate (Int-12):**

Prepared according to General Procedure A. ^{1}H NMR (400 MHz, DMSO-d_6): δ ppm 8.13–8.11 (m, 2H), 7.90 (d, J = 8.0 Hz, 1H), 7.74–7.70 (m, 1H), 7.64–7.59 (m, 1H), 7.42 (d, J = 5.6 Hz, 1H), 3.61–3.59 (m, 4H), 3.28–3.25 (m, 4H), 1.45 (s, 9H): LCMS (ES, m/z) calculated for $C_{18}H_{24}N_3O_2$ [(M + H)$^+$] 314.19, observed 314.46.

4-(Isoquinolin-1-yl)piperazin-1-ium 2,2,2-trifluoroacetate (Int-13):

Prepared according to General Procedure B. ^{1}H NMR (400 MHz, DMSO-d_6): δ ppm 9.83 (brs, 2H), 8.30 (d, J = 8.8 Hz, 1H), 8.08 (d, J = 8.0 Hz, 1H), 8.0–7.94 (m, 2H), 7.79–7.75 (m, 1H), 7.65 (d, J = 6.4 Hz, 1H), 3.96 (s, 4H), 3.39 (s, 4H): LCMS (ES, m/z) calculated for $C_{13}H_{16}N_3$ [(M + H)$^+$] 214.13, observed 214.15

***tert*-Butyl (*R*)-(3-(3-chlorophenyl)-1-(4-(isoquinolin-1-yl)piperazin-1-yl)-1-oxopropan-2-yl)carbamate (Int-14a):**

Prepared according to General Procedure C. ^{1}H NMR (400 MHz, DMSO-d_6): δ ppm 8.14–8.11 (m, 2H), 7.90 (d, J = 8.0 Hz, 1H), 7.74–7.70 (m, 1H), 7.64–7.60 (m, 1H), 7.42 (d, J = 7.6 Hz, 1H), 7.37 (s, 1H), 7.33–7.20 (m, 4H), 4.70–4.64 (m, 1H), 3.81–3.66 (m, 4H), 3.25–3.18 (m, 4H), 2.97–2.91 (m, 1H), 2.85–2.81 (m, 1H), 1.31 (s, 9H): LCMS (ES, m/z) calculated for $C_{27}H_{32}ClN_4O_3$ [(M + H)$^+$] 495.22, observed 495.15

***tert*-Butyl(*R*)-(3-(2,4-dichlorophenyl)-1-(4-(isoquinolin-1-yl)piperazin-1-yl)-1-oxopropan-2-yl)carbamate (Int-14b):**

Prepared according to General Procedure C. ^{1}H NMR (400 MHz, DMSO-d_6): δ ppm 8.20–8.11 (m, 2H), 7.90 (d, J = 8.4 Hz, 1H), 7.75–7.71 (m, 1H), 7.64–7.58 (m, 2H), 7.42–7.36 (m, 3H), 7.23–7.21 (m, 1H), 4.76–4.75 (m, 1H), 3.75–3.68 (m, 4H), 3.28–3.25 (m, 4H), 3.18–3.12 (m, 1H), 2.96–2.92 (m, 1H), 1.28 (s, 9H): LCMS (ES, m/z) calculated for $C_{27}H_{31}Cl_2N_4O_3$ [(M + H)$^+$] 529.18, observed 529.05.

***tert*-Butyl 4-(6-bromoisoquinolin-1-yl)piperazine-1-carboxylate (Int-16):**

Prepared according to General Procedure A. ^{1}H NMR (400 MHz, DMSO-d_6): δ ppm 8.20–8.14 (m, 2H), 8.04 (d, J = 9.2 Hz, 1H), 7.71 (dd, J = 8.8, 2.0 Hz, 1H), 7.40 (d, J = 5.6 Hz, 1H), 3.59–3.57(m, 4H), 3.28–3.25 (m, 4H), 1.44 (s, 9H): LCMS (ES, m/z) calculated for $C_{18}H_{23}BrN_3O_2$ [(M + H)$^+$] 392.09, observed 391.94.

4-(6-Bromoisoquinolin-1-yl)piperazin-1-ium 2,2,2-trifluoroacetate (Int-17):

Prepared according to General Procedure B. ^{1}H NMR (400 MHz, DMSO-d_6): δ ppm 9.13 (brs, 1H), 8.26 (d, J = 2.0 Hz, 1H), 8.18 (d, J = 7.0 Hz, 1H), 8.11 (d, J = 9.2 Hz, 1H), 7.76–7.73 (m, 1H), 7.48 (d, J = 5.6 Hz, 1H), 3.57–3.55 (m, 4H), 3.33–3.18 (m, 4H): LCMS (ES, m/z) calculated for $C_{13}H_{15}BrN_3$ [(M + H)$^+$] 292.04, observed 292.06.

***tert*-Butyl (*R*)-(1-(4-(6-bromoisoquinolin-1-yl)piperazin-1-yl)-3-(3-chlorophenyl)-1-oxopropan-2-yl)carbamate (Int-18):**

Prepared according to General Procedure C. ^{1}H NMR (400 MHz, DMSO-d_6): δ ppm 8.20 (d, J = 2.0 Hz, 1H), 8.16 (d, J = 5.6 Hz, 1H), 8.06 (d, J = 8.8 Hz, 1H), 7.74–7.70 (m, 1H), 7.40 (d, J = 7.0 Hz, 1H), 7.36 (s, 1H), 7.33–7.19 (m, 4H), 4.67–4.65 (m, 1H), 3.82–3.62 (m, 4H), 3.33–3.18 (m, 4H), 2.92–2.82 (m, 2H), 1.34 (s, 9H): LCMS (ES, m/z) calculated for $C_{27}H_{31}BrClN_4O_3$ [(M + H)$^+$] 573.13, observed 573.05.

***tert*-Butyl(*R*)-(1-(4-(6-(1*H*-pyrazol-4-yl)isoquinolin-1-yl)piperazin-1-yl)-3-(3-chlorophenyl)-1-oxopropan-2-yl)carbamate (Int-19a):**

Prepared according to General Procedure D. ^{1}H NMR (400 MHz, DMSO-d_6): δ ppm 13.11 (s, 1H), 8.41 (s, 1H), 8.12–8.06 (m, 5H), 7.88 (dd, J = 8.4, 1.6 Hz, 1H), 7.35–7.21 (m, 5H), 4.67–4.64 (m, 1H), 3.81–3.78 (m, 2H), 3.72–3.68 (m, 2H), 3.26–3.18 (m, 4H), 2.93–2.91 (m, 1H), 2.86–2.84 (m, 1H), 1.32 (s, 9H); LCMS (ES, m/z) calculated for $C_{30}H_{34}ClN_6O_3$ [(M + H)$^+$] 561.24, observed 561.25.

***tert*-Butyl ((2*R*)-3-(3-chlorophenyl)-1-oxo-1-(4-(6-(1-(tetrahydro-2*H*-pyran-2-yl)-1*H*-pyrazol-5-yl)isoquinolin-1-yl)piperazin-1-yl)propan-2-yl)carbamate (Int-19b):**

Prepared according to General Procedure D. ^{1}H NMR (400 MHz, DMSO-d_6): δ ppm 8.26–8.18 (m, 2H), 8.06 (s, 1H), 7.75 (dd, J = 8.4 Hz, 1.2 Hz, 1H), 7.65 (d, J = 1.4 Hz, 1H), 7.47 (d, J = 7.0 Hz, 1H), 7.38–7.22 (m, 5H), 6.64 (d, J = 1.6 Hz, 1H), 5.34–5.31 (m, 1H), 4.69–4.67 (m, 1H), 3.86–3.71 (m, 4H), 3.64–3.58 (m, 2H), 3.28–3.17 (m, 4H), 2.94–2.92 (m, 1H), 2.84–2.82 (m, 1H), 196–1.94 (m, 1H), 1.82–1.78 (m, 1H), 1.58–1.52 (m, 4H), 1.32 (s, 9H); LCMS (ES, m/z) calculated for $C_{35}H_{42}ClN_6O_4$ [(M + H)$^+$] 645.31, observed 645.15.

7-Bromo-2-hydrazinylquinoline (Int-21):

Prepared according to General Procedure E. ^{1}H NMR (400 MHz, DMSO-d_6): δ ppm 8.30 (brs, 1H), 7.90 (d, J = 8.8 Hz, 1H), 7.67 (s, 1H), 7.60 (d, J = 8.4 Hz, 1H), 7.29 (dd, J = 8.4, 2.0 Hz, 1H), 6.88 (d, J = 8.4 Hz, 1H), 4.37 (brs, 2H); LCMS (ES, m/z) calculated for $C_9H_9BrN_3$ [(M + H)$^+$] 238.05, observed 238.07.

8-Bromo-1-methyl-[1,2,4]triazolo[4,3-*a*]quinoline (Int-22):

Prepared according to General Procedure F. ^{1}H NMR (400 MHz, DMSO-d_6): δ ppm 8.41 (d, J = 2.0 Hz, 1H), 7.98 (d, J = 8.4 Hz, 1H), 7.82 (dd, J = 8.4, 2.0 Hz, 1H), 7.75 (d, J = 9.6 Hz, 1H), 7.68 (d, J = 9.6 Hz, 1H), 3.09 (s, 3H); LCMS (ES, m/z) calculated for $C_{11}H_9BrN_3$ [(M + H)$^+$] 262.04, observed 262.06.

***tert*-Butyl 4-(1-methyl-[1,2,4]triazolo[4,3-*a*]quinolin-8-yl)piperazine-1-carboxylate (Int-23):**

Prepared according to General Procedure G. ^{1}H NMR (400 MHz, DMSO-d_6): δ ppm 7.82 (d, J = 8.4 Hz, 1H), 7.65 (s, 1H), 7.58 (d, J = 9.2 Hz, 1H), 7.33 (d, J = 9.6 Hz, 1H), 7.29 (d, J = 8.8 Hz, 1H), 3.55–3.52 (m, 4H), 3.41–3.37 (m, 4H), 3.13 (s, 3H), 1.44 (s, 9H); LCMS (ES, m/z) calculated for $C_{20}H_{26}N_5O_2$ [(M + H)$^+$] 368.21, observed 368.23.

4-(1-Methyl-[1,2,4]triazolo[4,3-*a*]quinolin-8-yl)piperazin-1-ium 2,2,2-trifluoroacetate (Int-24):

Prepared according to General Procedure B. ^{1}H NMR (400 MHz, DMSO-d_6): δ ppm 9.52 (brs, 2H), 7.87 (d, J = 8.4 Hz, 1H), 7.68 (s, 1H), 7.62 (d, J = 8.8 Hz, 1H), 7.54 (d, J = 9.6 Hz, 1H), 7.34 (d, J = 8.8 Hz, 1H), 3.98–3.96 (m, 4H), 3.36–3.34 (m, 4H), 3.12 (s, 3H); LCMS (ES, m/z) calculated for $C_{15}H_{18}N_5$ [(M + H)$^+$] 268.16, observed 268.08.

***tert*-Butyl (*R*)-(3-(3-chlorophenyl)-1-(4-(1-methyl-[1,2,4]triazolo[4,3-*a*]quinolin-8-yl)piperazin-1-yl)-1-oxopropan-2-yl)carbamate (Int-25a):**

Prepared according to General Procedure C. ^{1}H NMR (400 MHz, DMSO-d_6): δ ppm 7.82 (d, J = 8.8 Hz, 1H), 7.61–7.57 (m, 2H), 7.36–7.32 (m, 2H), 7.31–7.23 (m, 5H), 4.68–4.66 (m, 1H), 3.73–3.62 (m, 4H), 3.46–3.38 (m, 4H), 3.12 (s, 3H), 3.00–2.98 (m, 1H), 2.84–2.82 (m, 1H), 1.32 (s, 9H); LCMS (ES, m/z) calculated for $C_{29}H_{34}ClN_6O_3$ [(M + H)$^+$] 549.24, observed 549.25.

tert-Butyl (R)-(3-(2,4-dichlorophenyl)-1-(4-(1-methyl-[1,2,4]triazolo[4,3-_a_]quinolin-8-yl)piperazin-1-yl)-1-oxopropan-2-yl)carbamate (Int-25b):

Prepared according to General Procedure C. ^{1}H NMR (400 MHz, DMSO-d_6): δ ppm 7.82 (d, J = 8.8 Hz, 1H), 7.62–7.57 (m, 3H), 7.41–7.32 (m, 3H), 7.30–7.25 (m, 2H), 4.78–4.74 (m, 1H), 3.71–3.62 (m, 4H), 3.28–3.22 (m, 4H), 3.12 (s, 3H), 3.09–3.06 (m, 1H), 2.94–2.89 (m, 1H), 1.29 (s, 9H); LCMS (ES, m/z) calculated for $C_{29}H_{33}Cl_2N_6O_3$ [(M + H)$^+$] 583.24, observed 583.05.

6-Bromo-1-hydrazinylisoquinoline (Int-27):

Prepared according to General Procedure E. ^{1}H NMR (400 MHz, DMSO-d_6): δ ppm 8.79 (s, 1H), 8.13 (d, J = 8.8 Hz, 1H), 8.00–7.94 (m, 2H), 7.62–7.59 (m, 1H), 6.91 (d, J = 5.6 Hz, 1H), 4.41 (brs, 2H): LCMS (ES, m/z) calculated for $C_9H_9BrN_3$ [(M + H)$^+$] 238.04, observed 238.06.

8-Bromo-3-methyl-[1,2,4]triazolo[3,4-a]isoquinoline (Int-28):

Prepared according to General Procedure F. ^{1}H NMR (400 MHz, DMSO-d_6): δ ppm 8.41 (d, J = 8.8 Hz, 1H), 8.26–8.23 (m, 2H), 7.91–7.87 (m, 1H), 7.30 (d, J = 7.2 Hz, 1H), 2.71 (s, 3H): LCMS (ES, m/z) calculated for $C_{11}H_9BrN_3$ [(M + H)$^+$] 262.03, observed 262.05.

tert-Butyl 4-(3-methyl-[1,2,4]triazolo[3,4-a]isoquinolin-8-yl)piperazine-1-carboxylate (Int-29):

Prepared according to General Procedure G. ^{1}H NMR (400 MHz, DMSO-d_6): δ ppm 8.29 (d, J = 8.8 Hz, 1H), 8.06 (d, J = 7.2 Hz, 1H), 7.44–7.41 (m, 1H), 7.34–7.33 (m, 1H), 7.14 (d, J = 7.2 Hz, 1H), 3.53–3.50 (m, 4H), 3.35–3.33 (m, 2H), 3.23–3.21 (m, 2H), 2.68 (s, 3H), 1.44 (s, 9H): LCMS (ES, m/z) calculated for $C_{20}H_{26}N_5O_2$ [(M + H)$^+$] 368.21, observed 368.24.

4-(3-Methyl-[1,2,4]triazolo[3,4-a]isoquinolin-8-yl)piperazin-1-ium 2,2,2-trifluoroacetate (Int-30):

Prepared according to General Procedure B. ^{1}H NMR (400 MHz, DMSO-d_6): δ ppm 9.30 (brs, 1H), 8.01–7.93 (m,, 2H), 7.71 (d, J = 2.4 Hz, 1H), 7.52 (d, J = 9.6 Hz, 1H), 7.47–7.44 (m, 1H), 3.72–3.68 (m, 4H), 3.31–3.29 (m, 4H), 3.21 (s, 3H): LCMS (ES, m/z) calculated for $C_{15}H_{18}N_5$ [(M + H)$^+$] 268.16, observed 268.05.

***tert*-Butyl (*R*)-(3-(3-chlorophenyl)-1-(4-(3-methyl-[1,2,4]triazolo[3,4-a]isoquinolin-8-yl)piperazin-1-yl)-1-oxopropan-2-yl)carbamate (Int-31a):**

Prepared according to General Procedure C. ^{1}H NMR (400 MHz, DMSO-d_6): δ ppm 8.29 (d, J = 9.2 Hz, 1H), 8.06 (d, J = 7.2 Hz, 1H), 7.43–7.39 (m, 1H), 7.36–7.23 (m, 6H), 7.15 (d, J = 7.6 Hz, 1H), 4.67–4.66 (m, 1H), 3.70–3.61 (m, 4H), 3.21–3.18 (m, 4H), 2.92–2.82 (m, 2H), 2.68 (s, 3H), 1.32 (s, 9H): LCMS (ES, m/z) calculated for $C_{29}H_{34}ClN_6O_3$ [(M + H)$^+$] 549.24, observed 549.10.

***tert*-Butyl (*R*)-(3-(2,4-dichlorophenyl)-1-(4-(3-methyl-[1,2,4]triazolo[3,4-a]isoquinolin-8-yl)piperazin-1-yl)-1-oxopropan-2-yl)carbamate (Int-31b):**

Prepared according to General Procedure C. ^{1}H NMR (400 MHz, DMSO-d_6): δ ppm 8.29 (d, J = 9.2 Hz, 1H), 8.06 (d, J = 7.6 Hz, 1H), 7.59 (d, J = 1.6 Hz, 1H), 7.43–7.31 (m, 4H), 7.24 (d, J = 9.2 Hz, 1H), 7.15 (d, J = 7.6 Hz, 1H), 4.75–4.73 (m, 1H), 3.71–3.62 (m, 4H), 3.28–3.23 (m, 4H), 3.06–3.04 (m, 1H), 2.92–2.88 (m, 1H), 2.67 (s, 3H), 1.28 (s, 9H): LCMS (ES, m/z) calculated for $C_{29}H_{33}Cl_2N_6O$ [(M + H)$^+$] 583.23, observed 583.25.

6-Bromo-2-(2-methyl-1H-imidazol-1-yl)quinoline (Int-33):

Prepared according to General Procedure A. ^{1}H NMR (400 MHz, DMSO-d_6): δ ppm 8.58 (d, J = 8.8 Hz, 1H), 8.38 (s, 1H), 7.96–7.95 (m, 2H), 7.88 (d, J = 8.8 Hz, 1H), 7.76 (d, J = 1.6 Hz, 1H), 6.99 (d, J = 1.6 Hz, 1H), 2.65 (s, 3H); LCMS (ES, m/z) calculated for $C_{13}H_{11}BrN_3$ [(M + H)$^+$] 288.01, observed 288.06

***tert*-Butyl 4-(2-(2-methyl-1H-imidazol-1-yl)quinolin-6-yl)piperazine-1-carboxylate (Int-34):**

Prepared according to General Procedure G. ^{1}H NMR (400 MHz, DMSO-d_6): δ ppm 8.36 (d, J = 8.8 Hz, 1H), 7.86 (d, J = 9.2 Hz, 1H), 7.71–7.64 (m, 3H), 7.32 (s, 1H), 6.95 (s, 1H), 3.62–3.60 (m, 4H), 3.28–3.24 (m, 4H), 2.58 (s, 3H), 1.44 (s, 9H); LCMS (ES, m/z) calculated for $C_{22}H_{28}N_5O_2$ [(M + H)$^+$] 394.22, observed 394.24.

4-(2-(2-Methyl-1H-imidazol-1-yl)quinolin-6-yl)piperazin-1-ium 2,2,2-trifluoroacetate (Int-35):

Prepared according to General Procedure B. ^{1}H NMR (400 MHz, DMSO-d_6): δ ppm 9.38 (brs, 2H), 8.55 (d, *J* = 8.4 Hz, 1H), 8.21 (s, 1H), 7.98 (d, *J* = 9.6 Hz, 1H), 7.87–7.81 (m, 3H), 7.48 (s, 1H), 3.54–3.51 (m, 4H), 3.28–3.26 (m, 4H), 2.86 (s, 3H); LCMS (ES, m/z) calculated for $C_{17}H_{20}N_5$ [(M + H)$^+$] 294.17, observed 294.15.

***tert*-Butyl (*R*)-(3-(3-chlorophenyl)-1-(4-(2-(2-methyl-1H-imidazol-1-yl)quinolin-6-yl)piperazin-1-yl)-1-oxopropan-2-yl)carbamate (Int-36a):**

Prepared according to General Procedure C. ^{1}H NMR (400 MHz, DMSO-d_6): δ ppm 8.37 (d, *J* = 8.8 Hz, 1H), 7.86 (d, *J* = 9.6 Hz, 1H), 7.71–7.64 (m, 3H), 7.36 (s, 1H), 7.32–7.24 (m, 5H), 6.95 (d, *J* = 1.6 Hz, 1H), 4.69–4.67 (m, 1H), 3.72–3.62 (m, 4H), 3.28–3.14 (m, 4H), 2.94–2.92 (m, 1H), 2.86–2.84 (m, 1H), 2.58 (s, 3H), 1.32 (s, 9H): LCMS (ES, m/z) calculated for $C_{31}H_{36}ClN_6O_3$ [(M + H)$^+$] 575.25, observed 575.24.

***tert*-Butyl (*R*)-(3-(2,4-dichlorophenyl)-1-(4-(2-(2-methyl-1H-imidazol-1-yl)quinolin-6-yl)piperazin-1-yl)-1-oxopropan-2-yl)carbamate (Int-36b):**

Prepared according to General Procedure C. ^{1}H NMR (400 MHz, DMSO-d_6): δ ppm 8.33 (d, *J* = 8.4 Hz, 1H), 7.86 (d, *J* = 9.2 Hz, 1H), 7.72–7.58 (m, 4H), 7.41–7.23 (m, 4H), 6.95 (d, *J* = 1.6 Hz, 1H), 4.78–4.72 (m, 1H), 3.62–3.58 (m, 4H), 3.26–3.18 (m, 4H), 3.04–3.02 (m, 1H), 2.94–2.92 (m, 1H), 2.58 (s, 3H), 1.29 (s, 9H); LCMS (ES, m/z) calculated for $C_{31}H_{35}Cl_2N_6O_3$ [(M + H)$^+$] 609.21, observed 609.15.

***tert*-Butyl 4-(1-methyl-[1,2,4]triazolo[4,3-*a*]quinolin-7-yl)-3-oxopiperazine-1-carboxylate (Int-38):**

To the stirred solution of 7-bromo-1-methyl-[1,2,4]triazolo[4,3-*a*]quinoline (comp **37**) (1g, 3.83 mmol, 1eq) and tert-butyl 3-oxopiperazine-1-carboxylate (1.06 g, 5.74 mmol, 1.5 eq) in DMF (25 ml) were added CuI (0.073 g, 0.38 mmol, 0.1 eq), TEMDA (0.088 g, 0.76 mmol, 0.2 eq) and Cs_2CO_3 (3.73 g, 11.49 mmol, 3 eq). Then degassed the reaction mixture with argon for 10 min and heated to 120 ^{0}C for 16h. Reaction was monitored by TLC. After completion of the reaction, the reaction mixture was diluted with water (100 ml) and extracted with EtOAc (3 x 2 100 ml). Dried the organic layer over Na_2SO_4. Concentrated the organic layer under reduced pressure. The resultant crude was purified by using combi flash to obtain the product (Yield:

57%). ^{1}H NMR (400 MHz, DMSO-d_6): δ ppm 8.38 (d, J = 8.8 Hz, 1H), 8.02 (s, 1H), 7.78–7.65 (m, 3H), 4.15–4.14 (m, 2H), 3.94–3.92 (m, 2H), 3.62–3.61 (m, 2H), 3.14 (s, 3H), 1.46 (s, 9H); LCMS (ES, m/z) calculated for $C_{20}H_{24}N_5O_3$ [(M + H)$^+$] 382.19, observed 382.23.

4-(1-Methyl-[1,2,4]triazolo[4,3-*a*]quinolin-7-yl)-3-oxopiperazin-1-ium 2,2,2-trifluoroacetate (Int-39):

Prepared according to General Procedure B. ^{1}H NMR (400 MHz, DMSO-d_6): δ ppm 9.98 (brs, 2H), 8.52 (d, J = 9.2 Hz, 1H), 8.09 (s, 1H), 8.00 (d, J = 9.6 Hz, 1H), 7.86–7.79 (m, 2H), 4.06–4.04 (m, 2H), 3.87–3.85 (m, 2H), 3.76–3.74 (m, 2H), 3.17 (s, 3H); LCMS (ES, m/z) calculated for $C_{15}H_{16}N_5O$ [(M + H)$^+$] 282.14, observed 282.05.

***tert*-Butyl (*R*)-(3-(3-chlorophenyl)-1-(4-(1-methyl-[1,2,4]triazolo[4,3-*a*]quinolin-7-yl)-3-oxopiperazin-1-yl)-1-oxopropan-2-yl)carbamate (Int-40a):**

Prepared according to General Procedure C. ^{1}H NMR (400 MHz, DMSO-d_6): δ ppm 8.39 (d, J = 9.2 Hz, 1H), 7.98 (s, 1H), 7.75–7.65 (m, 3H), 7.38–7.25 (m, 4H), 4.67–4.64 (m, 1H), 4.34–4.32 (m, 1H), 4.07–3.97 (m, 1H), 3.92–3.72 (m, 4H), 3.64–3.62 (m, 1H), 3.08 (s, 3H), 2.92–2.82 (m, 2H), 1.34 (s, 9H); LCMS (ES, m/z) calculated for $C_{29}H_{32}ClN_6O_4$ [(M + H)$^+$] 563.22, observed 563.26.

***tert*-Butyl (*R*)-(3-(2,4-dichlorophenyl)-1-(4-(1-methyl-[1,2,4]triazolo[4,3-*a*]quinolin-7-yl)-3-oxopiperazin-1-yl)-1-oxopropan-2-yl)carbamate (Int-40b)**:

Prepared according to General Procedure C. ^{1}H NMR (400 MHz, DMSO-d_6): δ ppm 8.39 (d, J = 9.2 Hz, 1H), 7.99–7.98 (m, 1H), 7.75–7.66 (m, 3H), 7.58 (s, 1H), 7.44–7.31 (m, 3H), 4.75–4.71 (m, 1H), 4.32–4.28 (m, 1H), 3.97–3.86 (m, 1H), 3.92–3.72 (m, 4H), 3.18–3.16 (m, 1H), 3.08 (s, 3H), 2.94–2.92 (m, 1H), 1.29 (s, 9H); LCMS (ES, m/z) calculated for $C_{29}H_{31}Cl_2N_6O_4$ [(M + H)$^+$] 597.18, observed 597.27.

***tert*-Butyl 8-(1-methyl-[1,2,4]triazolo[4,3-*a*]quinolin-7-yl)-3,8-diazabicyclo[3.2.1]octane-3-carboxylate (Int-41):**

Prepared according to General Procedure G. ^{1}H NMR (400 MHz, DMSO-d_6): δ ppm 8.15 (d, J = 9.6 Hz, 1H), 7.60–7.52 (m, 2H), 7.37–7.30 (m, 2H), 4.29–4.26 (m, 2H), 3.64–3.62 (m, 2H),

3.01 (s, 3H), 2.91–2.88 (m, 2H), 1.88–1.84 (m, 4H), 1.43 (s, 9H); LCMS (ES, m/z) calculated for $C_{22}H_{28}N_5O_2$ [(M + H)$^+$] 394.22, observed 394.15.

8-(1-Methyl-[1,2,4]triazolo[4,3-*a*]quinolin-7-yl)-3-azabicyclo[3.2.1]octan-3-ium 2,2,2-trifluoroacetate (Int-42):

Prepared according to General Procedure B. ^{1}H NMR (400 MHz, DMSO-*d*$_6$): δ ppm 9.88 (brs, 2H), 8.35 (d, *J* = 9.2 Hz, 1H), 8.09 (d, *J* = 9.6 Hz, 1H), 7.80 (d, *J* = 9.6 Hz, 1H), 7.61–7.52 (m, 2H), 4.23–4.21 (m, 2H), 3.68–3.52 (m, 2H), 3.34–3.32 (m, 2H), 3.18 (s, 3H), 2.04–1.98 (m, 4H); LCMS (ES, m/z) calculated for $C_{17}H_{20}N_5$ [(M + H)$^+$] 294.17, observed 294.21.

***tert*-Butyl ((2*R*)-3-(3-chlorophenyl)-1-(8-(1-methyl-[1,2,4]triazolo[4,3-*a*]quinolin-7-yl)-3,8-diazabicyclo[3.2.1]octan-3-yl)-1-oxopropan-2-yl)carbamate (Int-43a):**

Prepared according to General Procedure C. ^{1}H NMR (400 MHz, DMSO-*d*$_6$): δ ppm 8.17 (d, *J* = 9.6 Hz, 1H), 7.61–7.52 (m, 2H), 7.36–7.22 (m, 7H), 4.72–4.42 (m, 3H), 3.68–3.52 (m, 4H), 3.03–2.98 (m, 5H), 1.92–1.62 (m, 4H), 1.32 (s, 9H); LCMS (ES, m/z) calculated for $C_{31}H_{36}ClN_6O_3$ [(M + H)$^+$] 575.20, observed 575.42.

***tert*-Butyl ((2R)-3-(2,4-dichlorophenyl)-1-(8-(1-methyl-[1,2,4]triazolo[4,3-*a*]quinolin-7-yl)-3,8-diazabicyclo[3.2.1]octan-3-yl)-1-oxopropan-2-yl)carbamate (Int-43b):**

Prepared according to General Procedure C. ^{1}H NMR (400 MHz, DMSO-*d*$_6$): δ ppm 8.17 (d, *J* = 8.4 Hz, 1H), 7.68–7.42 (m, 4H), 7.38–7.12 (m, 4H), 4.72–4.42 (m, 3H), 3.68–3.52 (m, 4H), 3.03–2.98 (m, 5H), 1.98–1.88 (m, 4H), 1.29 (s, 9H); LCMS (ES, m/z) calculated for $C_{31}H_{35}Cl_2N_6O_3$ [(M + H)$^+$] 609.21, observed 609.24.

***tert*-Butyl 3-(1-methyl-[1,2,4]triazolo[4,3-*a*]quinolin-7-yl)-3,8-diazabicyclo[3.2.1]octane-8-carboxylate (Int-44):**

Prepared according to General Procedure G. ^{1}H NMR (400 MHz, DMSO-*d*$_6$): δ ppm 8.17 (d, *J* = 9.2 Hz, 1H), 7.55–7.52 (m, 2H), 7.43–7.42 (m, 1H), 7.33–7.30 (m, 1H), 4.44–4.42 (m, 2H), 3.66–3.56 (m, 2H), 3.24–3.22 (m, 2H), 3.02 (s, 3H), 1.99–1.97 (m, 2H), 1.74–1.72 (m, 2H), 1.43 (s, 9H); LCMS (ES, m/z) calculated for $C_{22}H_{28}N_5O_2$ [(M + H)$^+$] 394.22, observed 394.23.

3-(1-Methyl-[1,2,4]triazolo[4,3-*a*]quinolin-7-yl)-3,8-diazabicyclo[3.2.1]octan-8-ium 2,2,2-trifluoroacetate (Int-45):

Prepared according to General Procedure B. ^{1}H NMR (400 MHz, DMSO-d_6): δ ppm 9.48 (brs, 2H), 8.38 (d, *J* = 9.6 Hz, 1H), 8.06 (d, *J* = 9.6 Hz, 1H), 7.80 (d, *J* = 9.6 Hz, 1H), 7.67 (d, *J* = 2.4 Hz, 1H), 7.53 (dd, *J* = 9.6, 2.8 Hz, 1H), 4.98–4.96 (m, 2H), 3.48–3.46 (m, 2H), 3.18 (s, 3H), 3.06–3.04 (m, 2H), 2.24–2.18 (m, 4H); LCMS (ES, m/z) calculated for $C_{17}H_{20}N_5$ [(M + H)$^+$] 294.17, observed 294.26.

***tert*-Butyl ((2*R*)-3-(3-chlorophenyl)-1-(3-(1-methyl-[1,2,4]triazolo[4,3-*a*]quinolin-7-yl)-3,8-diazabicyclo[3.2.1]octan-8-yl)-1-oxopropan-2-yl)carbamate (Int-46a):**

Prepared according to General Procedure C. ^{1}H NMR (400 MHz, DMSO-d_6): δ ppm 8.17 (d, *J* = 9.6 Hz, 1H), 7.58–7.52 (m, 2H), 7.38–7.24 (m, 7H), 4.72–4.38 (m, 3H), 3.68–3.52 (m, 4H), 3.03–2.98 (m, 5H), 1.92–1.62 (m, 4H), 1.32 (s, 9H); LCMS (ES, m/z) calculated for $C_{31}H_{36}ClN_6O_3$ [(M + H)$^+$] 575.20, observed 575.43.

***tert*-Butyl ((2*R*)-3-(2,4-dichlorophenyl)-1-(3-(1-methyl-[1,2,4]triazolo[4,3-*a*]quinolin-7-yl)-3,8-diazabicyclo[3.2.1]octan-8-yl)-1-oxopropan-2-yl)carbamate (Int-46b):**

Prepared according to General Procedure C. ^{1}H NMR (400 MHz, DMSO-d_6): δ ppm 8.19 (d, *J* = 8.4 Hz, 1H), 7.68–7.56 (m, 4H), 7.38–7.16 (m, 4H), 4.68–4.38 (m, 3H), 3.68–3.42 (m, 4H), 3.03–2.87 (m, 5H), 1.97–1.76 (m, 4H), 1.29 (s, 9H): LCMS (ES, m/z) calculated for $C_{31}H_{35}Cl_2N_6O_3$ [(M + H)$^+$] 609.21, observed 609.27.

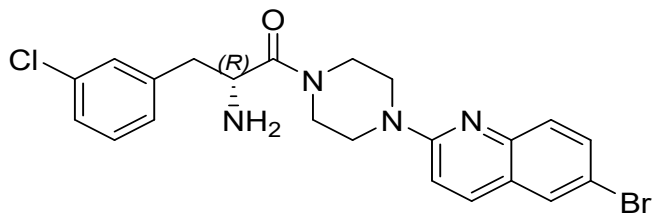

Chemical Formula: $C_{22}H_{22}BrClN_4O$
Exact Mass: 472.07

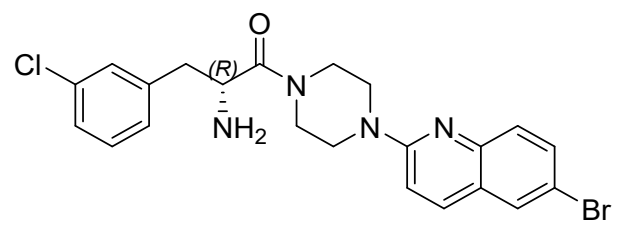

Chemical Formula: $C_{22}H_{22}BrClN_4O$
Exact Mass: 472.07

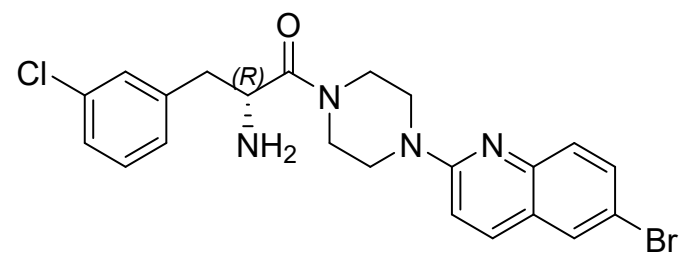

Chemical Formula: $C_{22}H_{22}BrClN_4O$
Exact Mass: 472.07

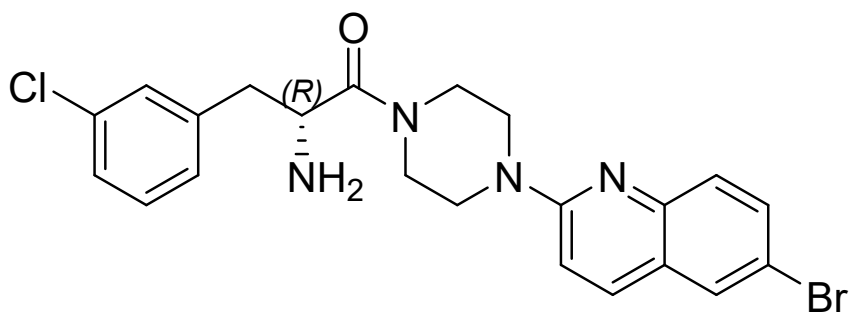

Chemical Formula: $C_{22}H_{22}BrClN_4O$
Exact Mass: 472.07

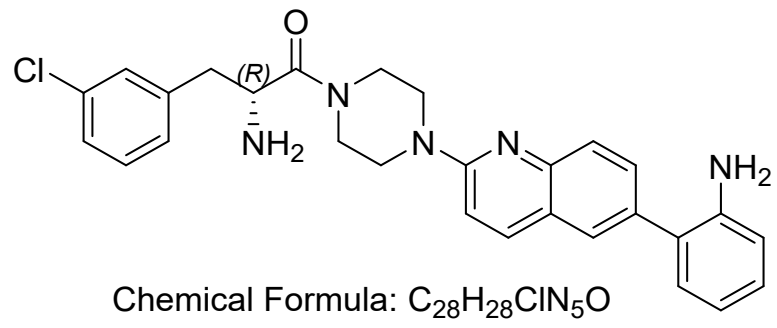

Chemical Formula: $C_{28}H_{28}ClN_5O$
Exact Mass: 485.20

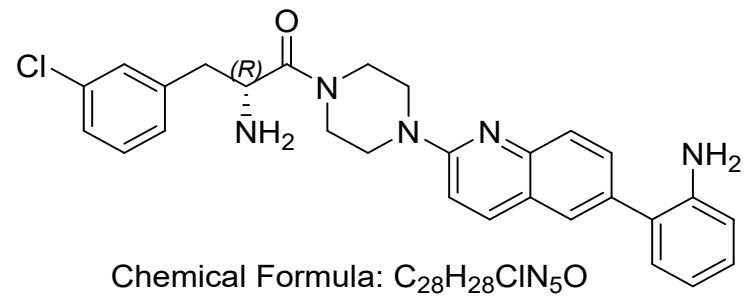

Chemical Formula: $C_{28}H_{28}ClN_5O$
Exact Mass: 485.20

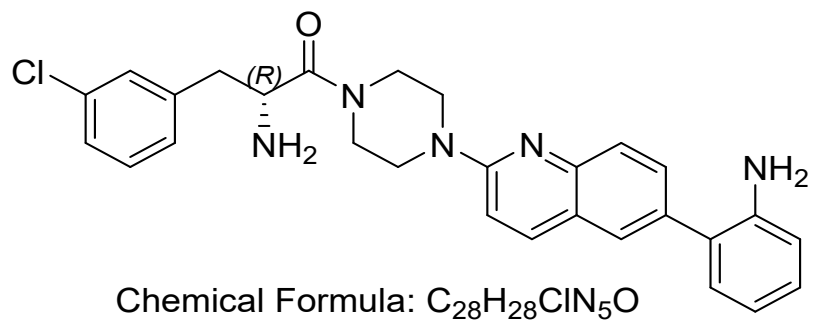

Chemical Formula: $C_{28}H_{28}ClN_5O$
Exact Mass: 485.20

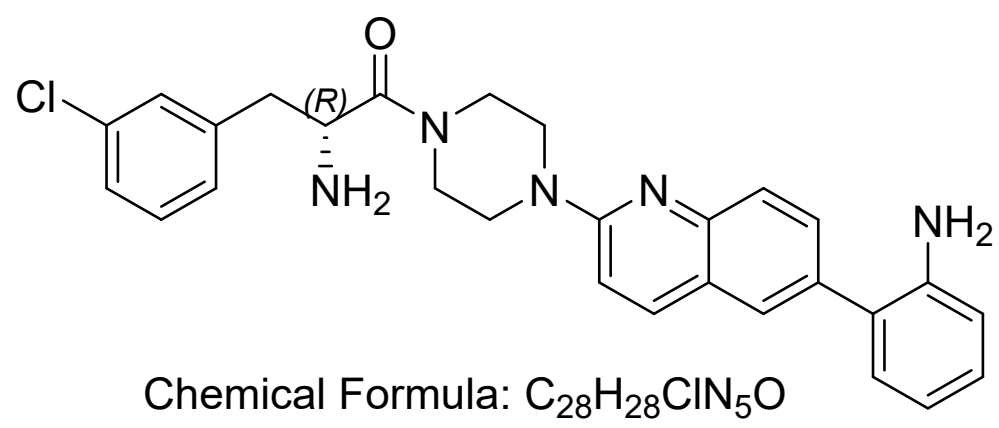

Chemical Formula: $C_{28}H_{28}ClN_5O$
Exact Mass: 485.20

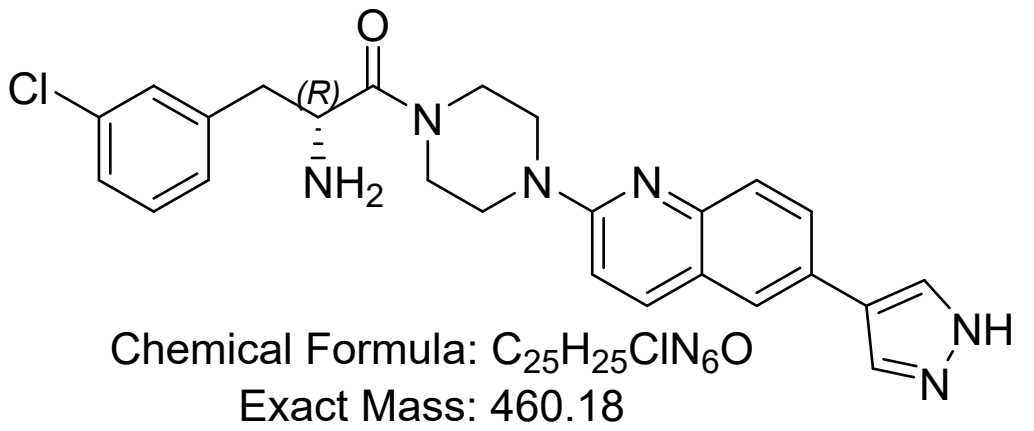

Chemical Formula: $C_{25}H_{25}ClN_6O$
Exact Mass: 460.18

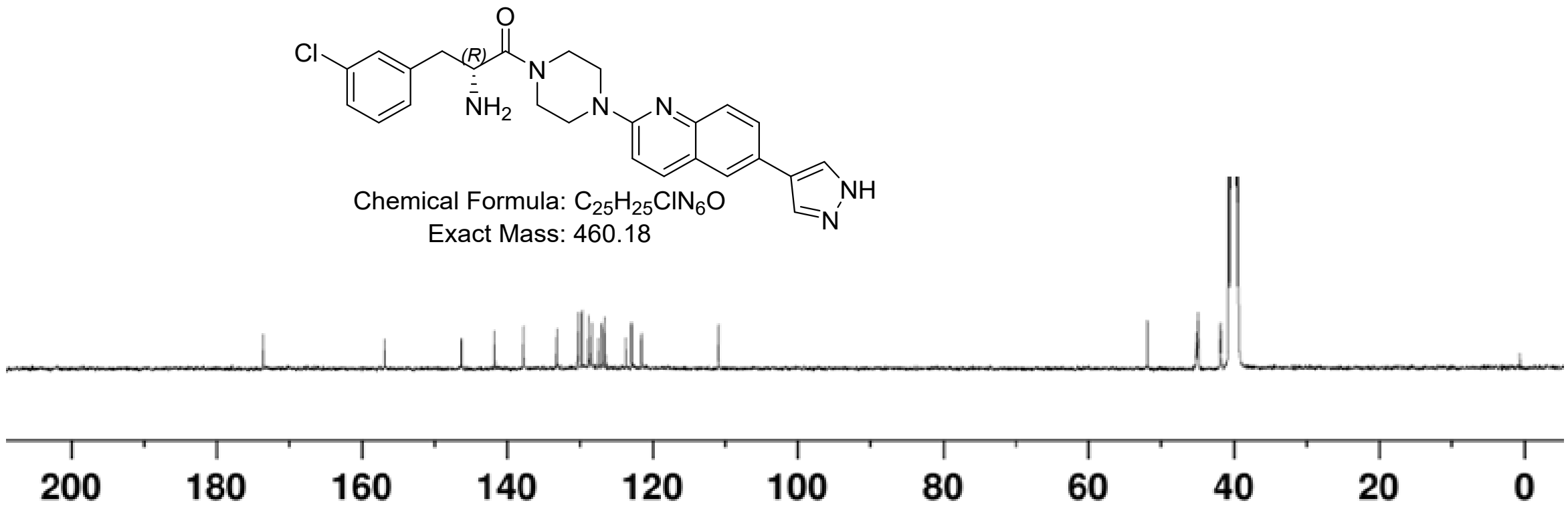

Figure S10: ^{13}C NMR of BHC-3

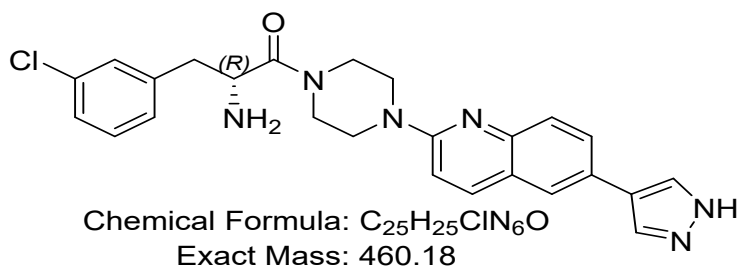

Chemical Formula: $C_{25}H_{25}ClN_6O$
Exact Mass: 460.18

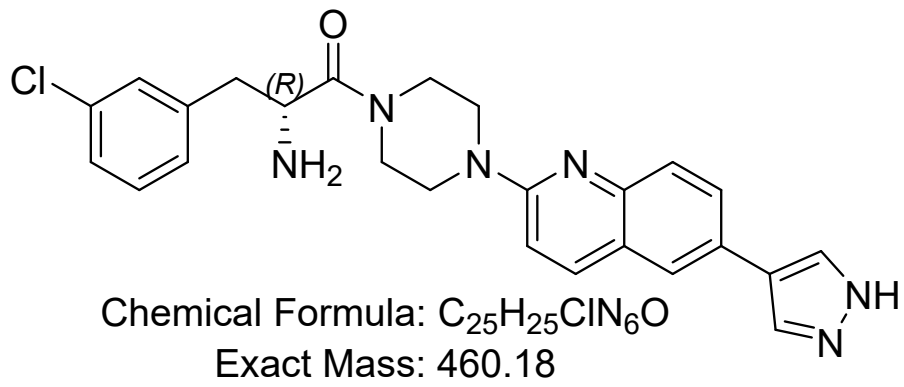

Chemical Formula: $C_{25}H_{25}ClN_6O$
Exact Mass: 460.18

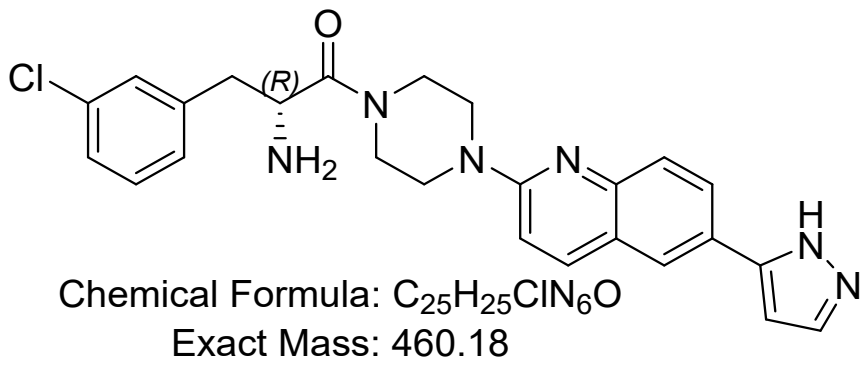

Chemical Formula: $C_{25}H_{25}ClN_6O$
Exact Mass: 460.18

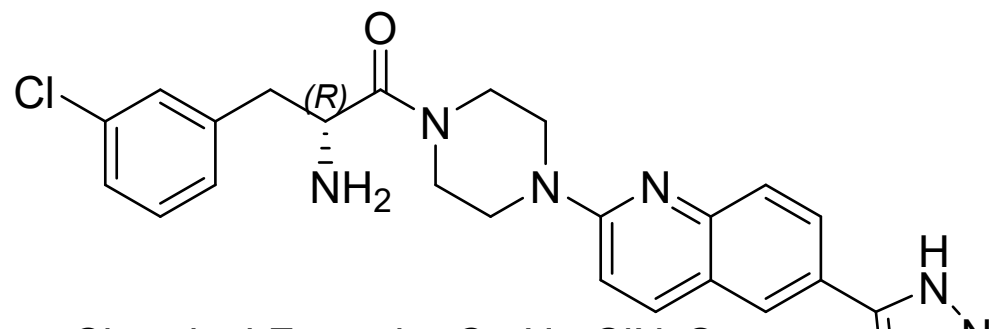

Chemical Formula: $C_{25}H_{25}ClN_6O$
Exact Mass: 460.18

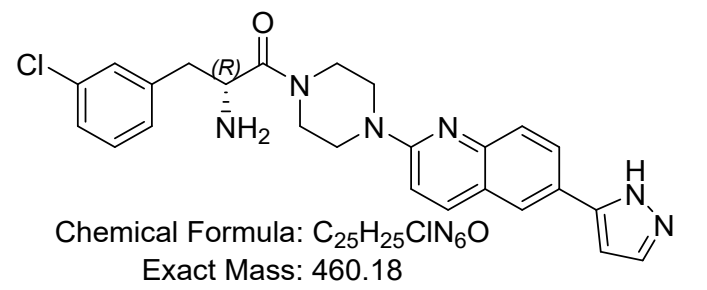

Chemical Formula: $C_{25}H_{25}ClN_6O$
Exact Mass: 460.18

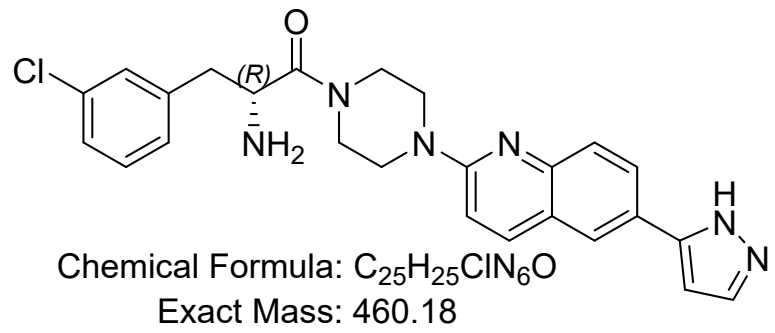

Chemical Formula: $C_{25}H_{25}ClN_6O$
Exact Mass: 460.18

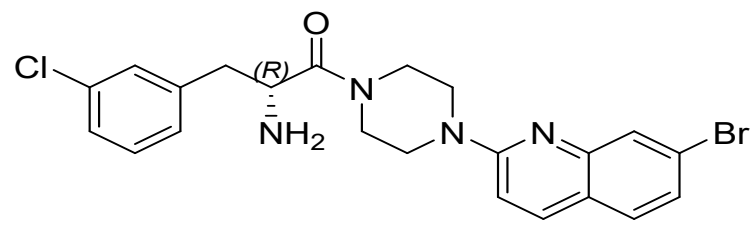

Chemical Formula: $C_{22}H_{22}BrClN_4O$
Exact Mass: 472.07

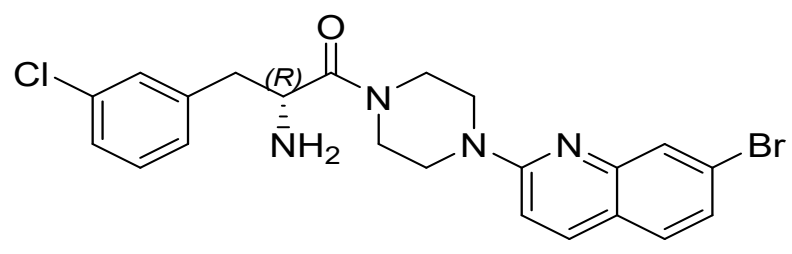

Chemical Formula: $C_{22}H_{22}BrClN_4O$
Exact Mass: 472.07

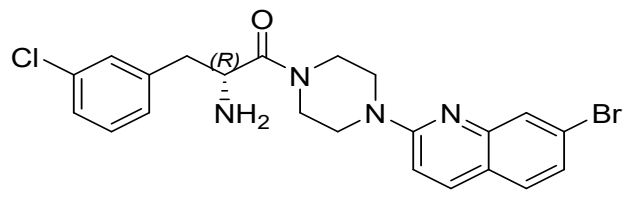

Chemical Formula: $C_{22}H_{22}BrClN_4O$
Exact Mass: 472.07

Figure S20: LC-MS of BHC-5

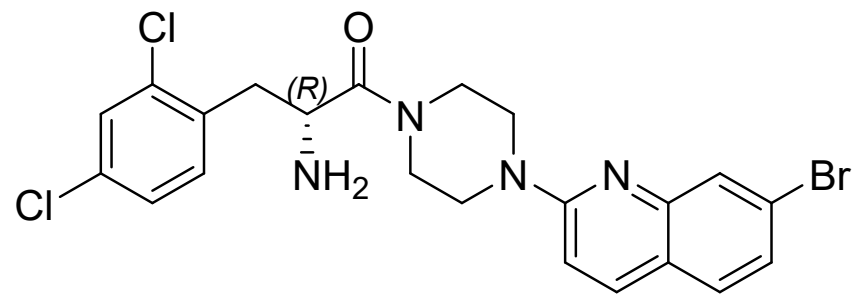

Chemical Formula: $C_{22}H_{21}BrCl_2N_4O$
Exact Mass: 506.03

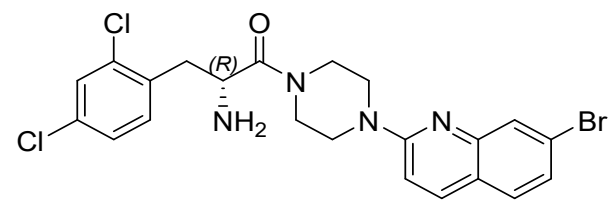

Chemical Formula: $C_{22}H_{21}BrCl_2N_4O$
Exact Mass: 506.03

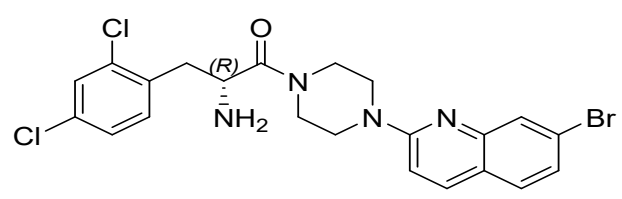

Chemical Formula: $C_{22}H_{21}BrCl_2N_4O$
Exact Mass: 506.03

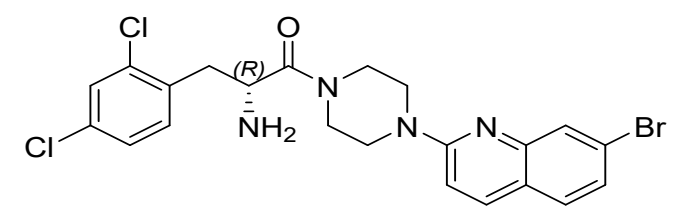

Chemical Formula: $C_{22}H_{21}BrCl_2N_4O$
Exact Mass: 506.03

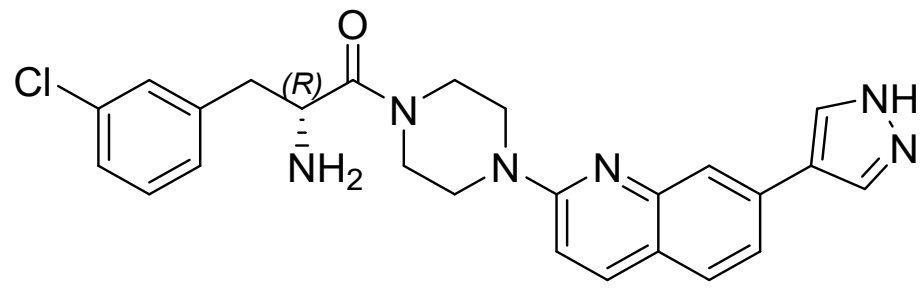

Chemical Formula: $C_{25}H_{25}ClN_6O$
Exact Mass: 460.18

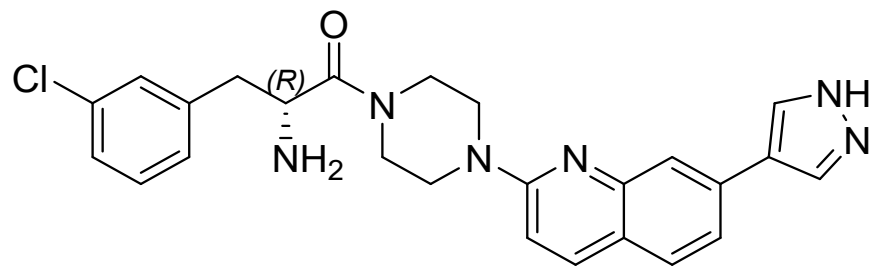

Chemical Formula: $C_{25}H_{25}ClN_6O$
Exact Mass: 460.18

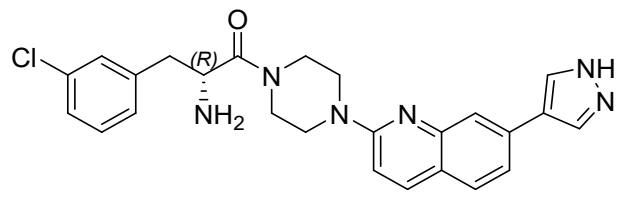

Chemical Formula: $C_{25}H_{25}ClN_6O$
Exact Mass: 460.18

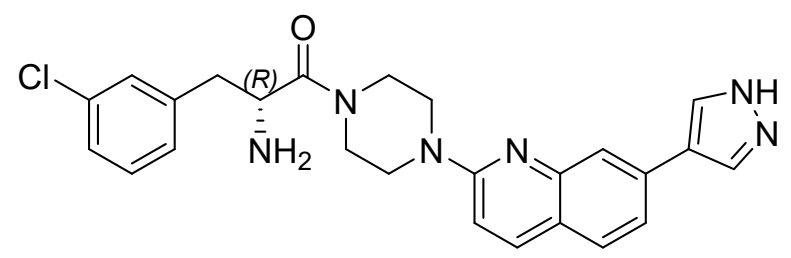

Chemical Formula: $C_{25}H_{25}ClN_6O$
Exact Mass: 460.18

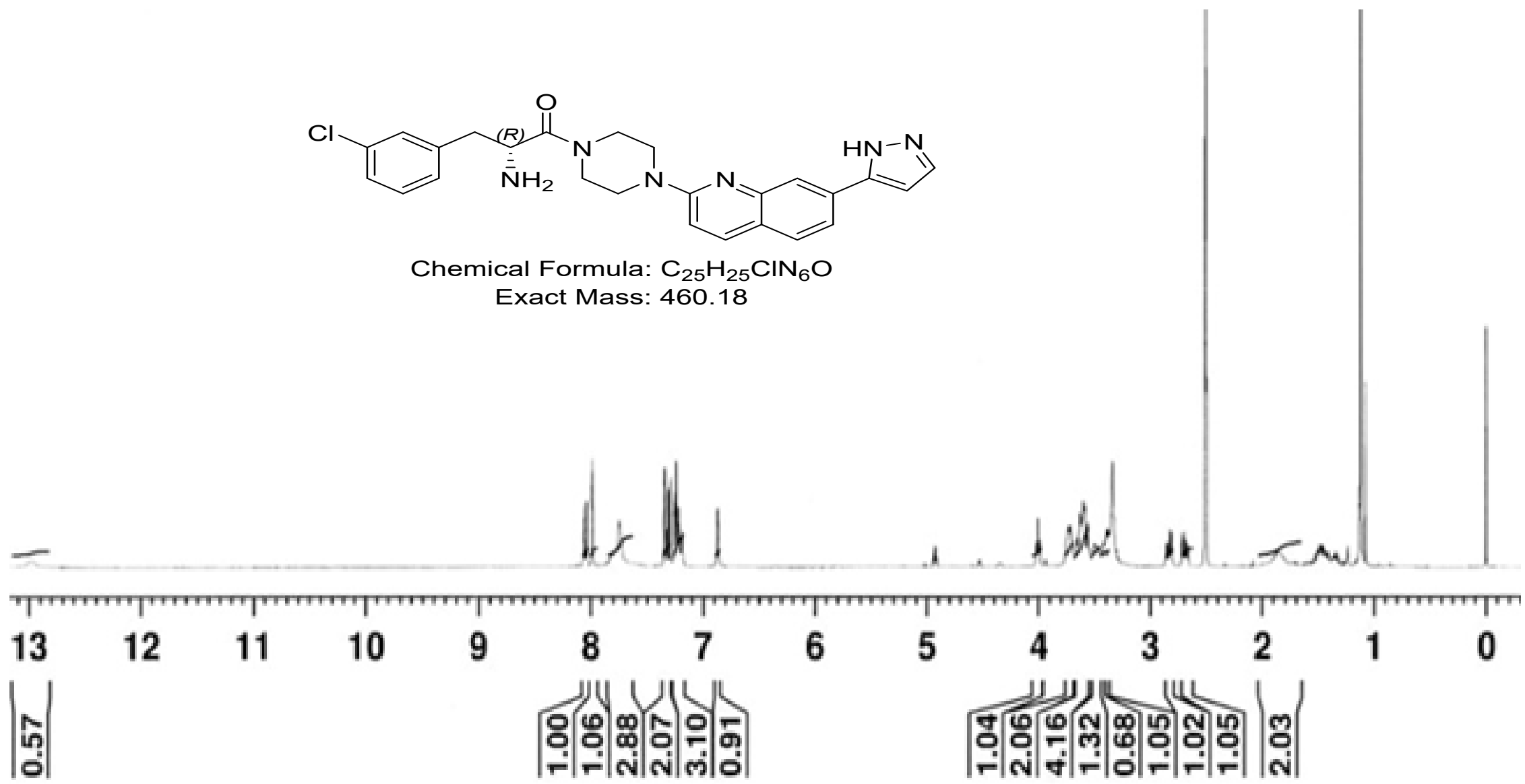

Figure S29: ^{1}H NMR of BHC-8

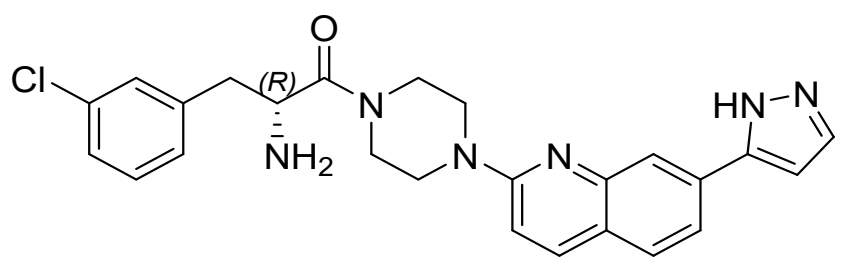

Chemical Formula: $C_{25}H_{25}ClN_6O$
Exact Mass: 460.18

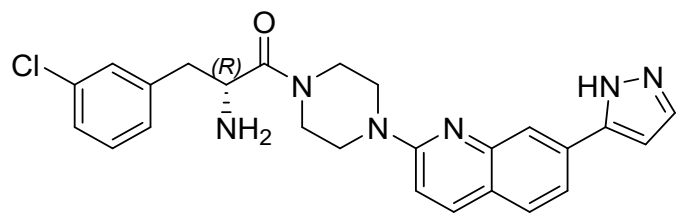

Chemical Formula: $C_{25}H_{25}ClN_6O$
Exact Mass: 460.18

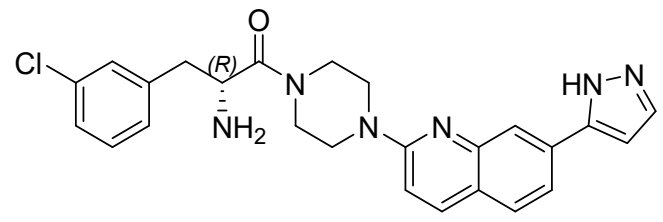

Chemical Formula: $C_{25}H_{25}ClN_6O$
Exact Mass: 460.18

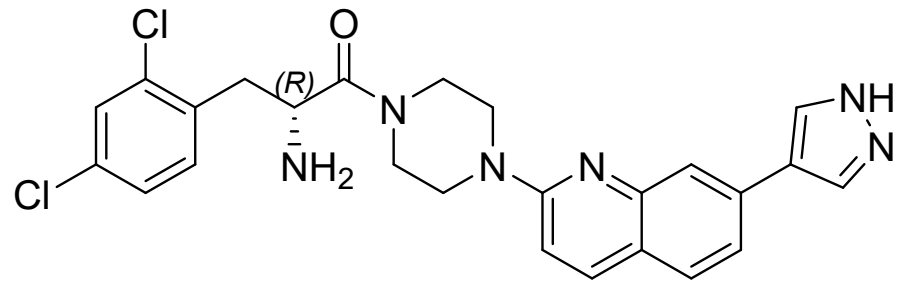

Chemical Formula: $C_{25}H_{24}Cl_2N_6O$
Exact Mass: 494.14

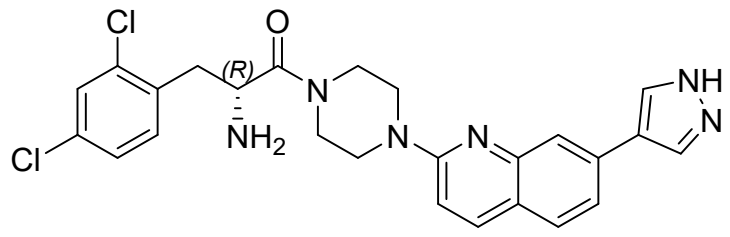

Chemical Formula: $C_{25}H_{24}Cl_2N_6O$
Exact Mass: 494.14

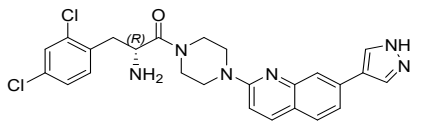

Chemical Formula: $C_{25}H_{24}Cl_2N_6O$
Exact Mass: 494.14

**Column**	:	**POROSHELL C18 (150mmx4.6mm,4μ)**
**Mobile phase**	:	**A: 0.01%TFA IN WATER B : ACETONITRILE**
**Flow rate**	:	**1.0 ml\min.**
**TEMP**	:	**40°C**
**GRADIENT**	:	**0/5,1/5,6/100,8/100,10/5,12/5**
**Diluent**	:	**ACN:WATER**

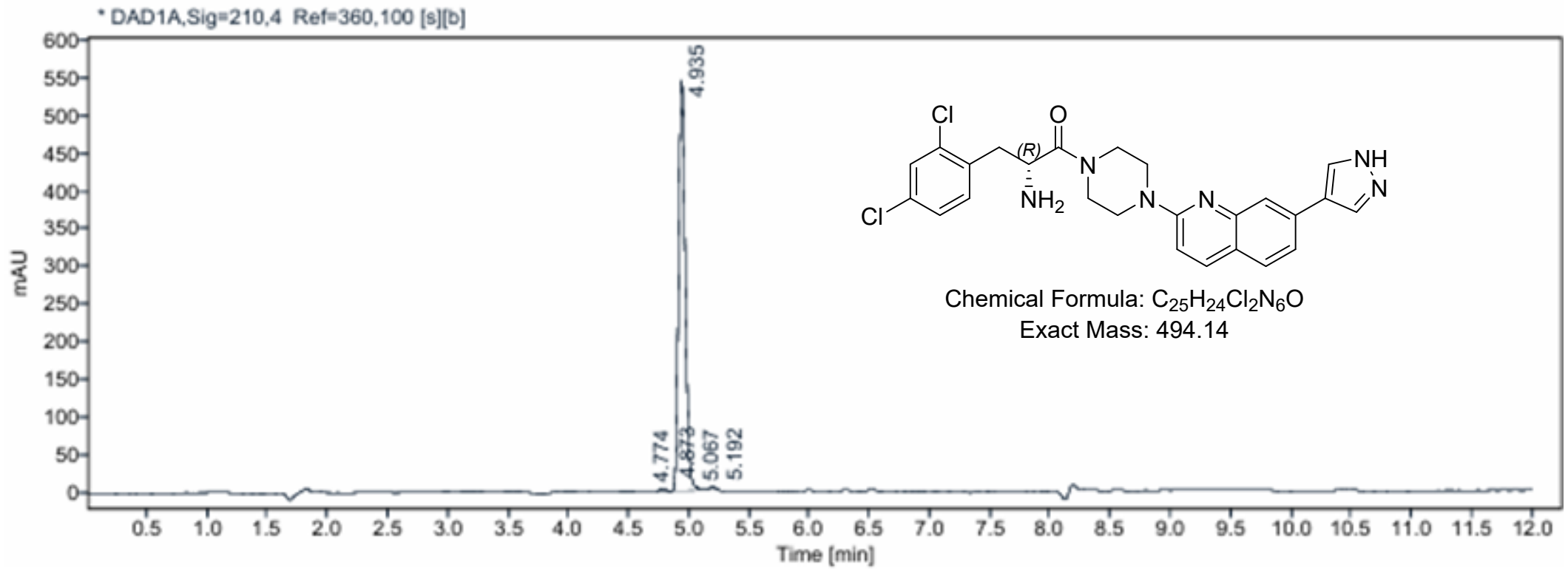

Signal: * DAD1A,Sig=210,4 Ref=360,100 [s][b]

RT [min]	Width [min]	Area	Area%
4.774	0.10	10.39	0.49
4.873	0.02	3.27	0.16
4.935	0.19	2072.52	98.31
5.067	0.08	8.37	0.40
5.192	0.10	13.64	0.65
	Sum	2108.19	

Figure S36: HPLC of BHC-9

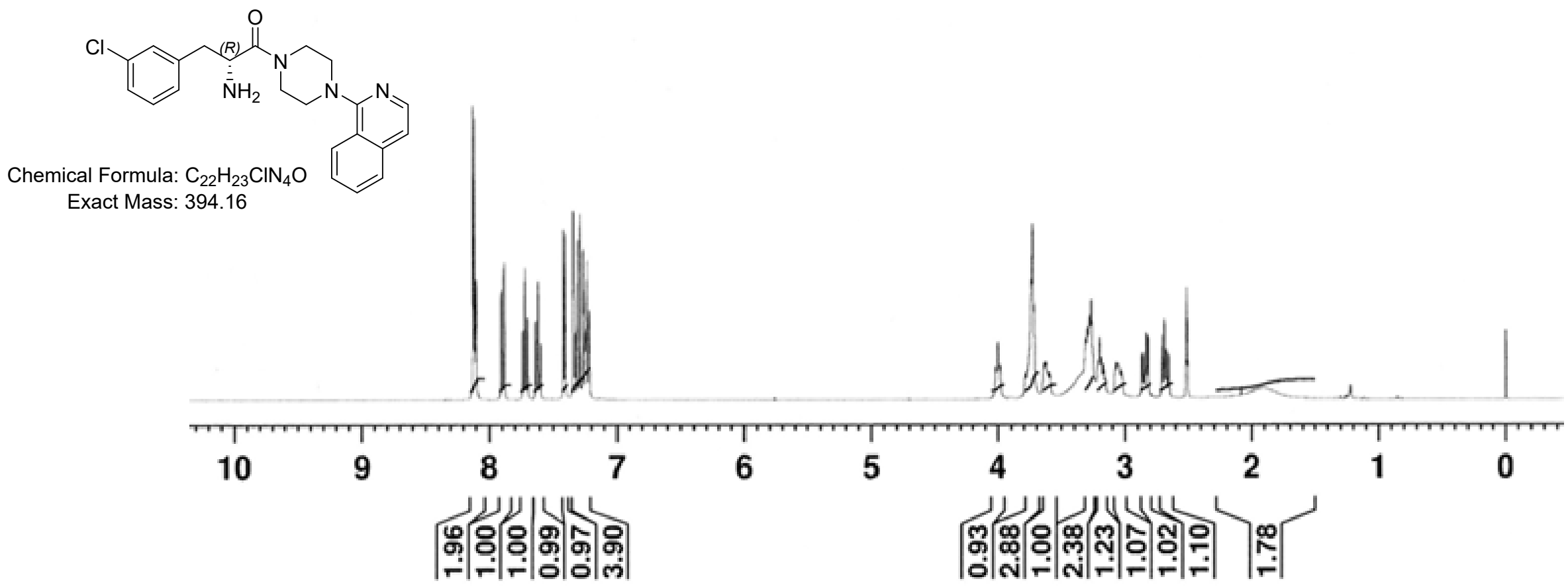

Figure S37: ^{1}H NMR of BHC-10

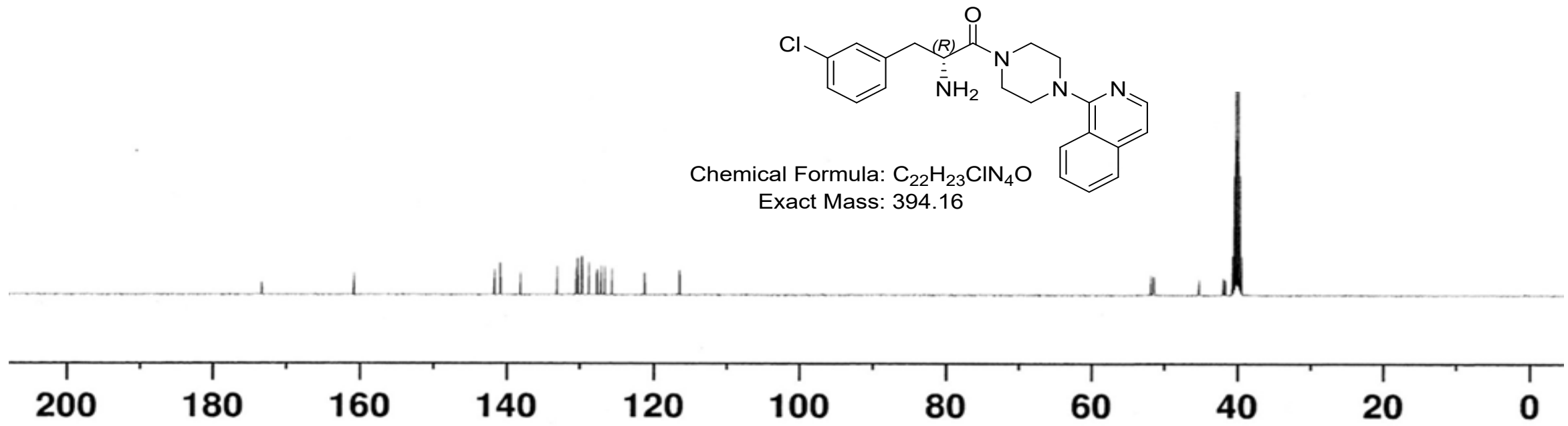

Figure S38: ^{13}C NMR of BHC-10

MOBILE PHASE : 0.1% Formic acid in Water(A)/ ACN(B)
GRADIENT/ (TIME/%B) : 0.01/20, 1.0/95, 2.0/95, 3.0/20 4.0/20
Column : ATLANTIS T3-C18,5 μM,4.6*50mm
Flow : 1.8 mL/min, Temp.: 40°C

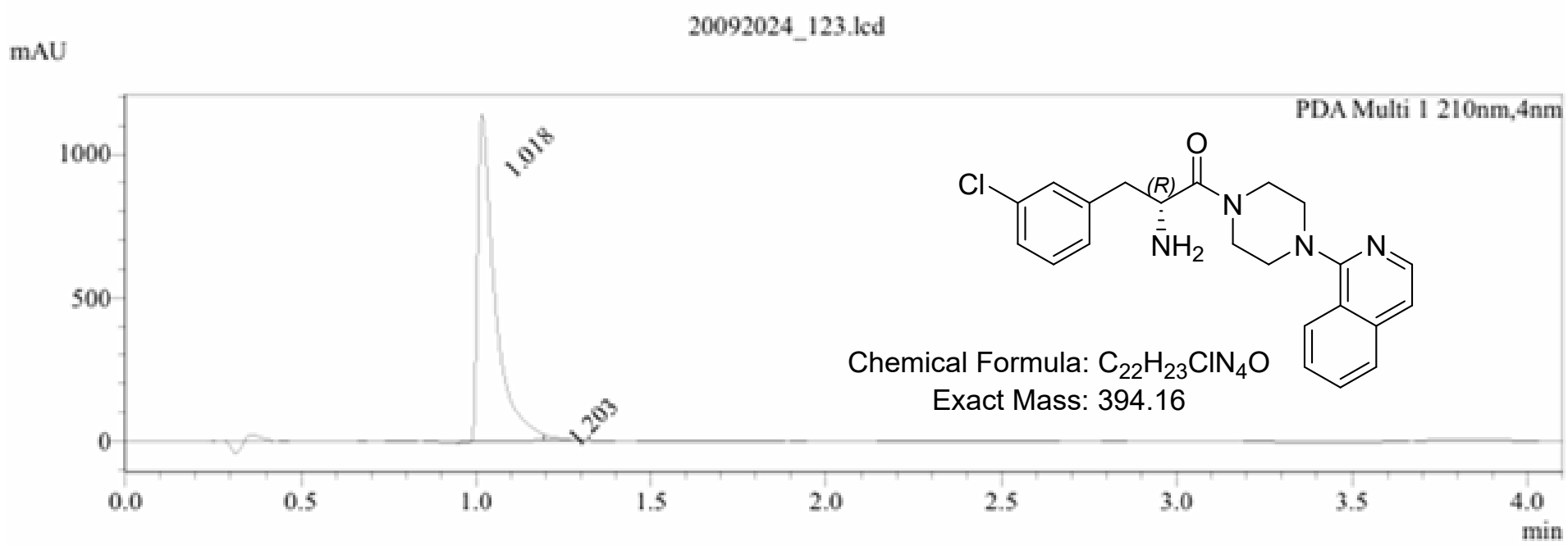

PDA Ch1 210nm

Peak#	Ret. Time	Area	Area%
1	1.018	4013802	99.331
2	1.203	27017	0.669
Total		4040819	100.000

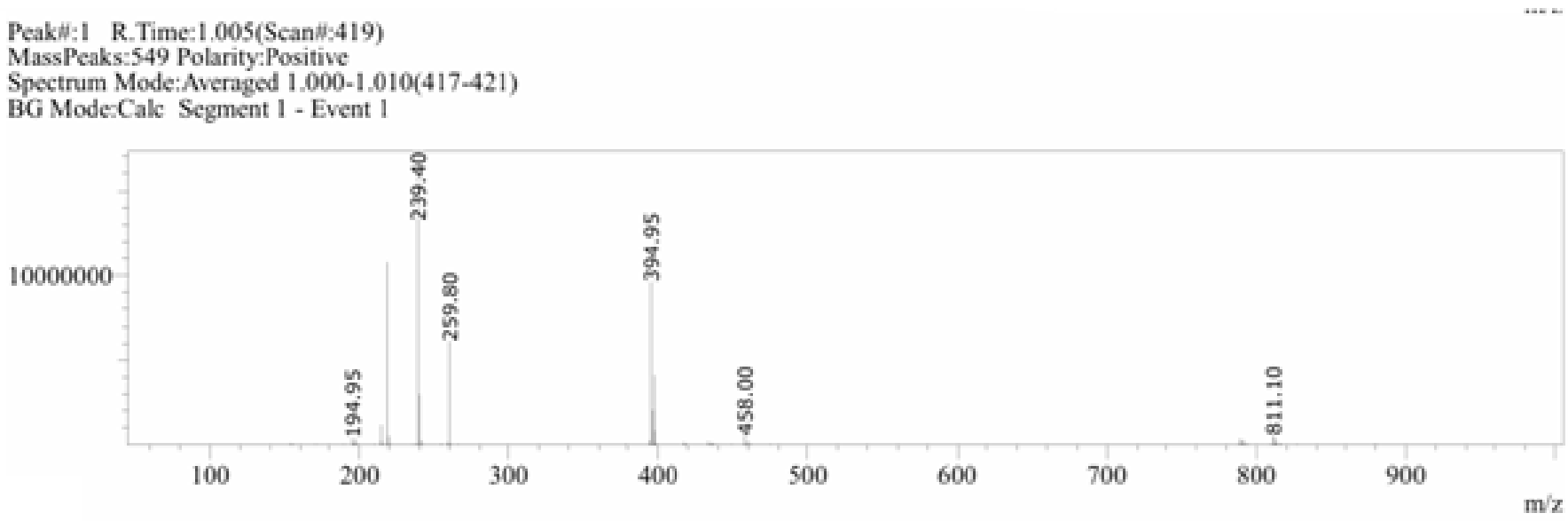

Figure S39: LC-MS of BHC-10

Column : **POROSHELL C18 (150mmx4.6mm,4μ)**
Mobile phase : **A: 0.01%TFA IN WATER B : ACETONITRILE**
Flow rate : **1.0 ml\min.**
TEMP : **40°C**
GRADIENT : **0/5,1/5,6/100,8/100,10/5,12/5**
Diluent : **ACN:WATER**

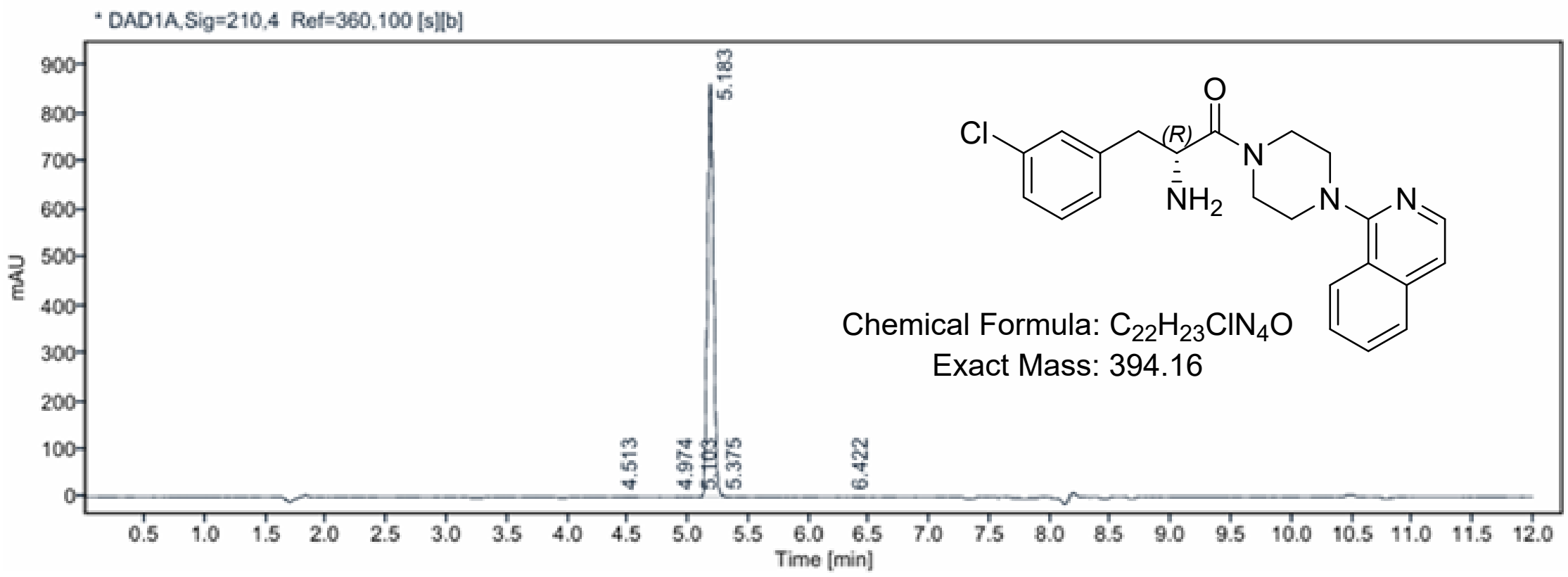

Signal: * DAD1A,Sig=210,4 Ref=360,100 [s][b]

RT [min]	Width [min]	Area	Area%
4.513	0.14	7.13	0.24
4.974	0.15	7.37	0.25
5.103	0.07	0.43	0.01
5.183	0.21	2964.77	99.00
5.375	0.20	9.68	0.32
6.422	0.12	5.41	0.18
	Sum	2994.78	

Figure S40: HPLC of BHC-10

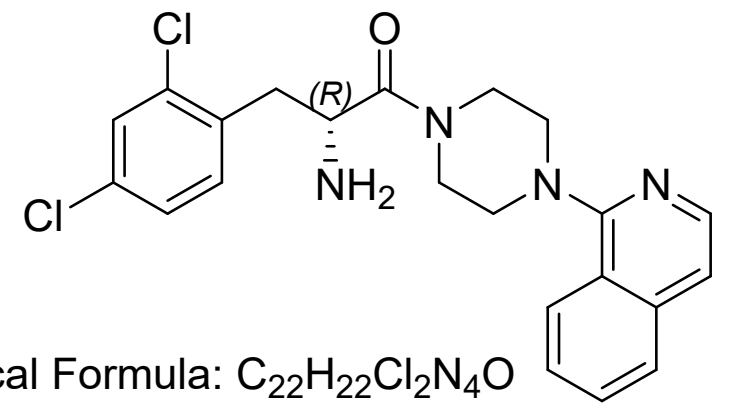

Chemical Formula: $C_{22}H_{22}Cl_2N_4O$
Exact Mass: 428.12

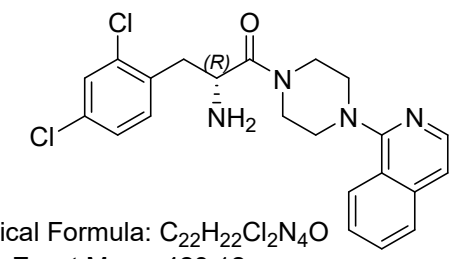

Chemical Formula: $C_{22}H_{22}Cl_2N_4O$
Exact Mass: 428.12

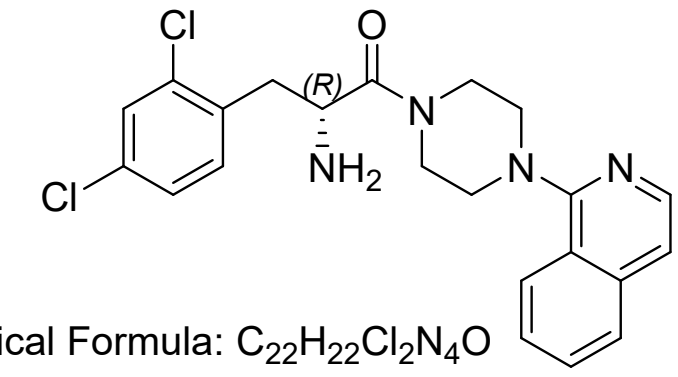

Chemical Formula: $C_{22}H_{22}Cl_2N_4O$
Exact Mass: 428.12

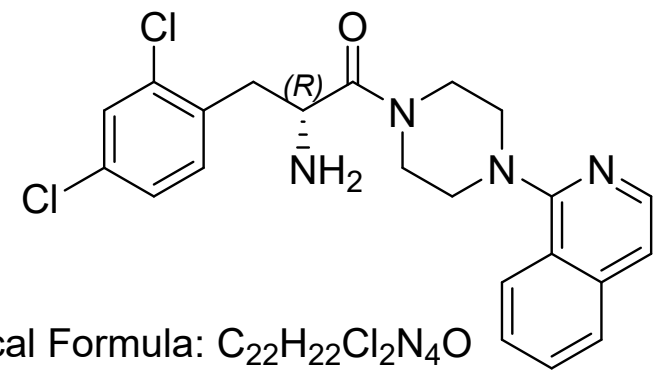

Chemical Formula: $C_{22}H_{22}Cl_2N_4O$
Exact Mass: 428.12

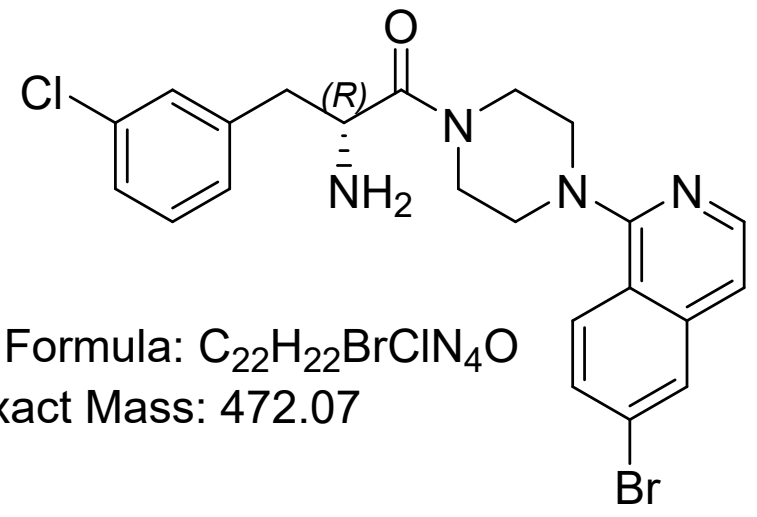

Chemical Formula: $C_{22}H_{22}BrClN_4O$
Exact Mass: 472.07

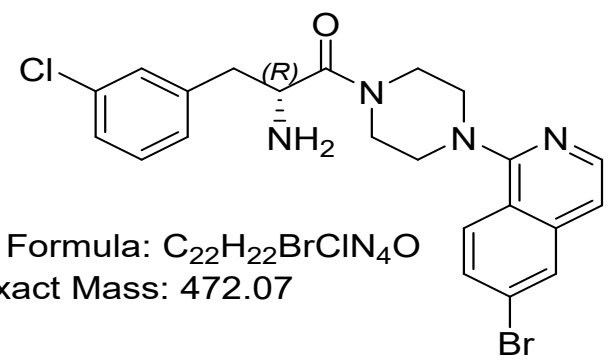

Chemical Formula: $C_{22}H_{22}BrClN_4O$
Exact Mass: 472.07

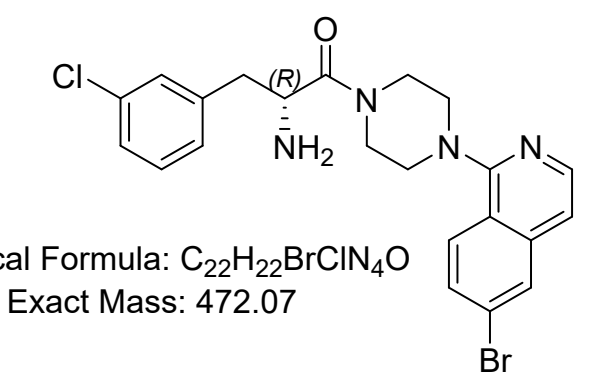

Chemical Formula: $C_{22}H_{22}BrClN_4O$
Exact Mass: 472.07

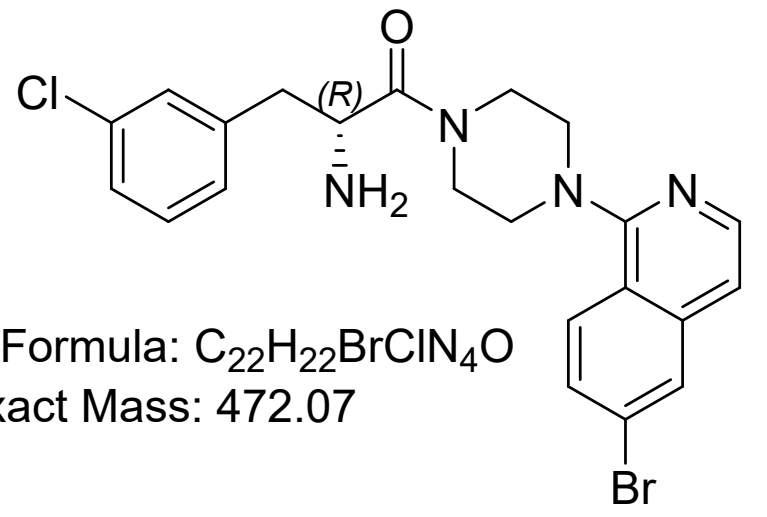

Chemical Formula: $C_{22}H_{22}BrClN_4O$
Exact Mass: 472.07

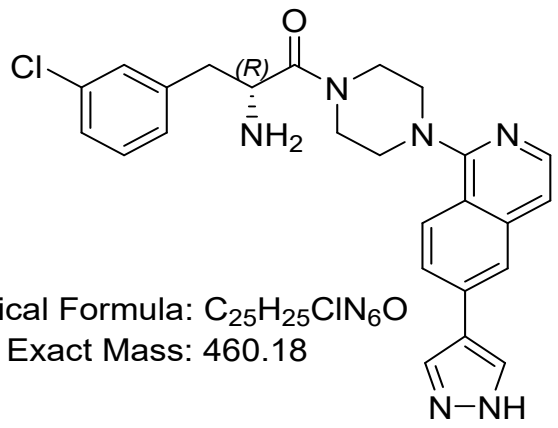

Chemical Formula: $C_{25}H_{25}ClN_6O$
Exact Mass: 460.18

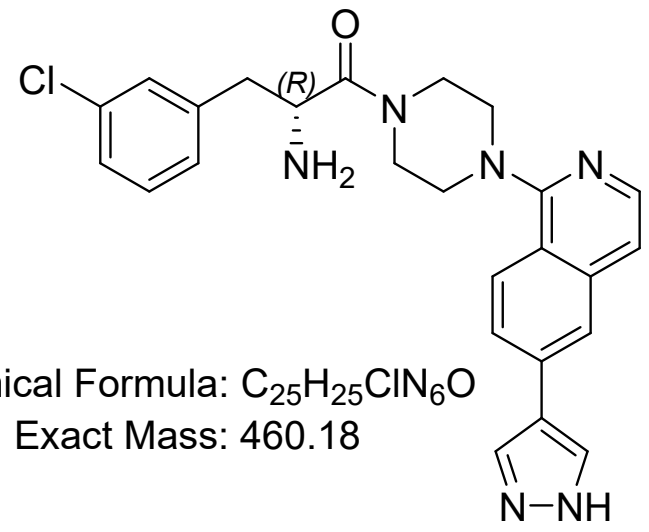

Chemical Formula: $C_{25}H_{25}ClN_6O$
Exact Mass: 460.18

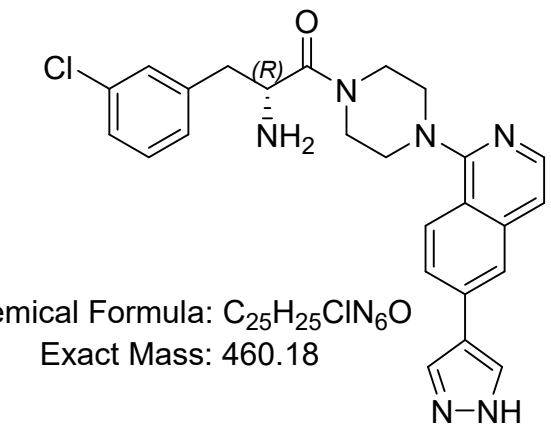

Chemical Formula: $C_{25}H_{25}ClN_6O$
Exact Mass: 460.18

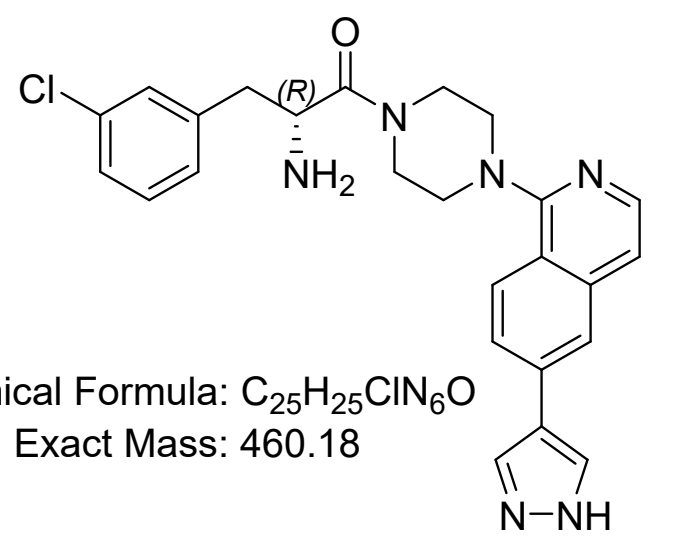

Chemical Formula: $C_{25}H_{25}ClN_6O$
Exact Mass: 460.18

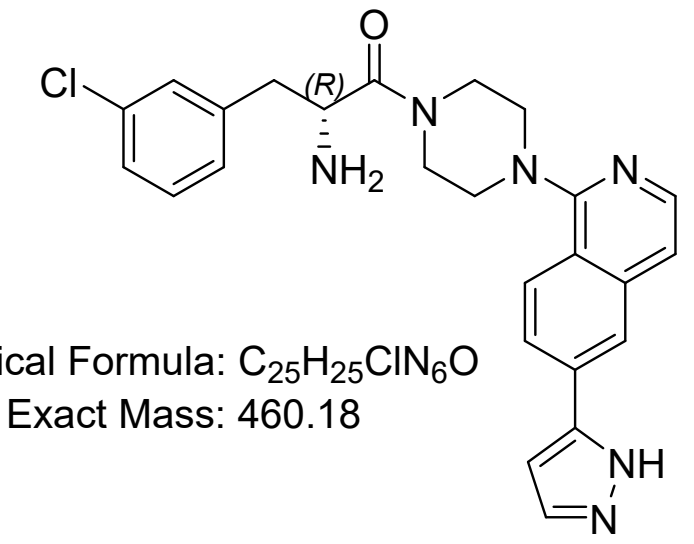

Chemical Formula: $C_{25}H_{25}ClN_6O$
Exact Mass: 460.18

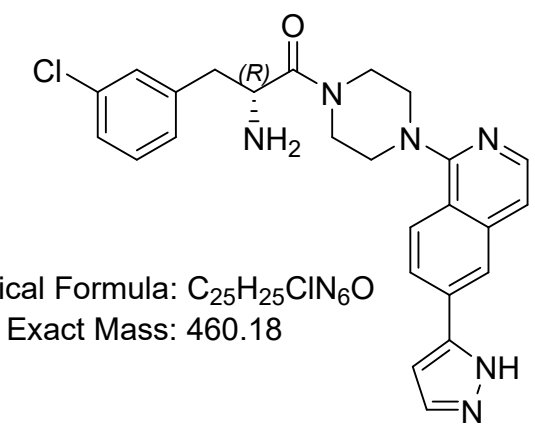

Chemical Formula: $C_{25}H_{25}ClN_6O$
Exact Mass: 460.18

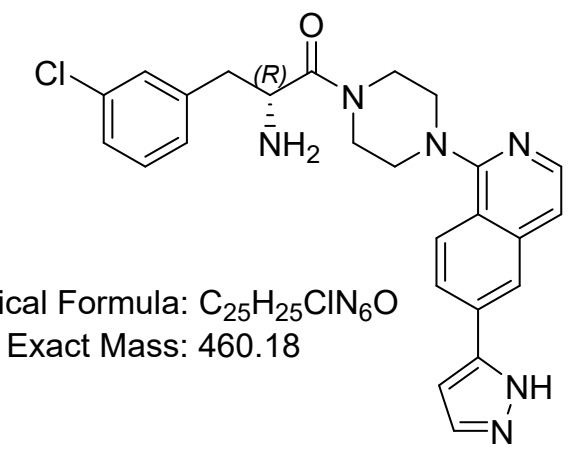

Chemical Formula: $C_{25}H_{25}ClN_6O$
Exact Mass: 460.18

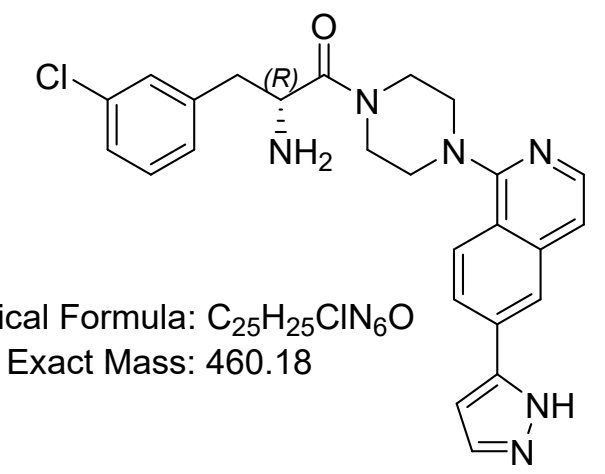

Chemical Formula: $C_{25}H_{25}ClN_6O$
Exact Mass: 460.18

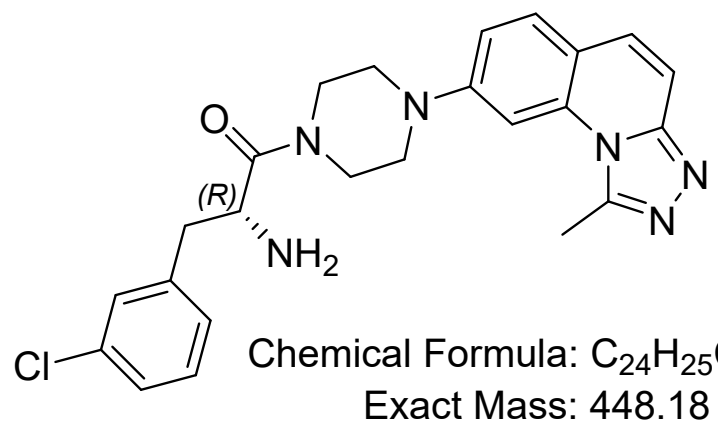

Chemical Formula: $C_{24}H_{25}ClN_6O$
Exact Mass: 448.18

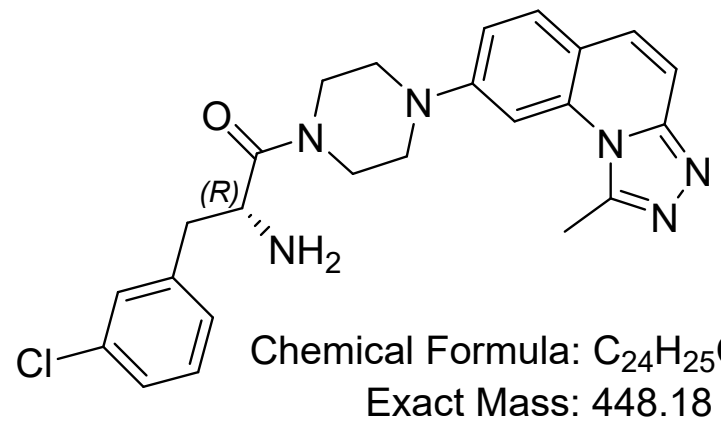

Chemical Formula: $C_{24}H_{25}ClN_6O$
Exact Mass: 448.18

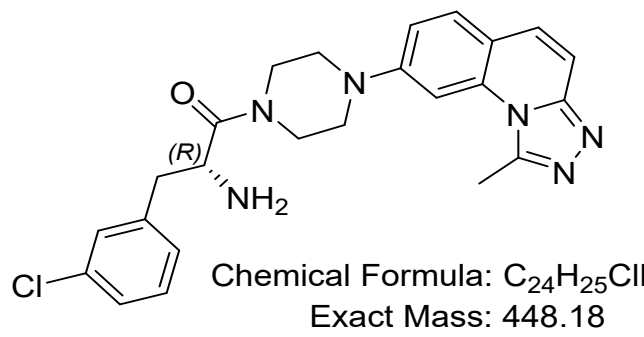

Chemical Formula: $C_{24}H_{25}ClN_6O$
Exact Mass: 448.18

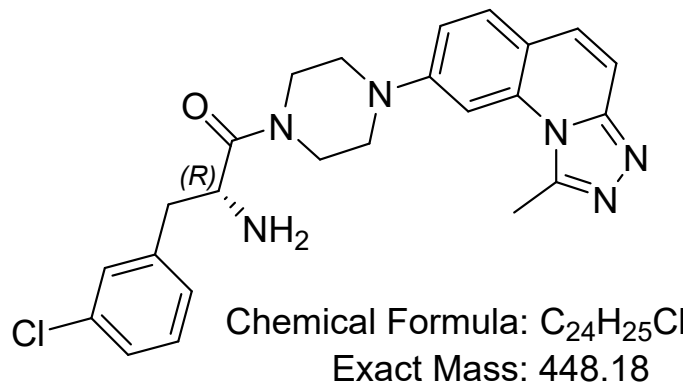

Chemical Formula: $C_{24}H_{25}ClN_6O$
Exact Mass: 448.18

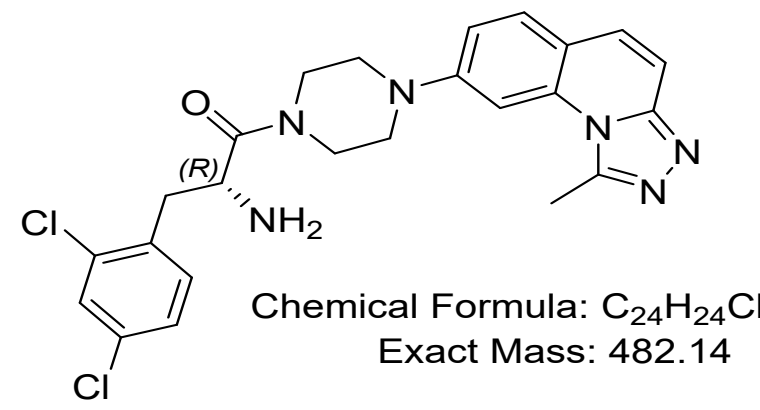

Chemical Formula: $C_{24}H_{24}Cl_2N_6O$
Exact Mass: 482.14

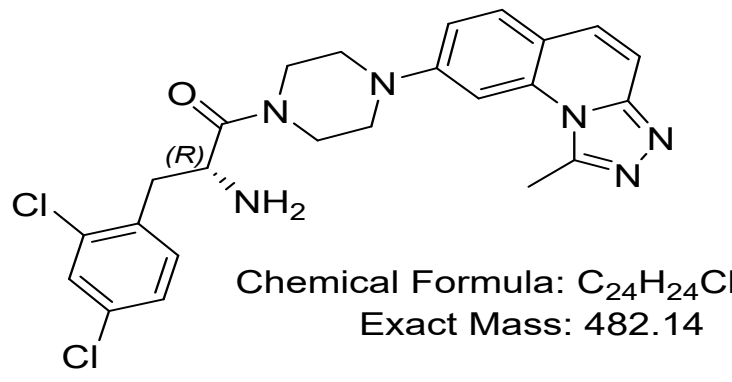

Chemical Formula: $C_{24}H_{24}Cl_2N_6O$
Exact Mass: 482.14

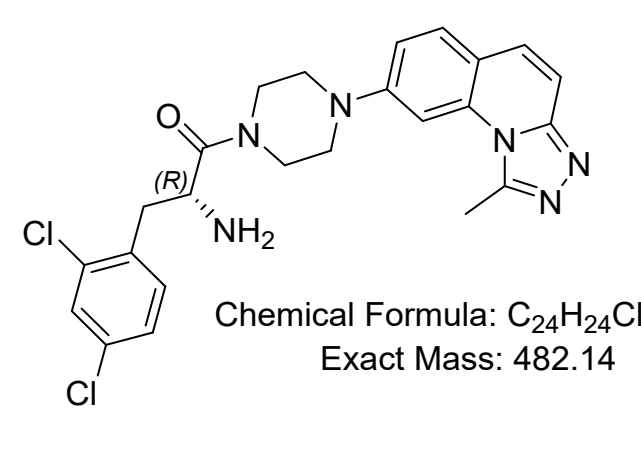

Chemical Formula: $C_{24}H_{24}Cl_2N_6O$
Exact Mass: 482.14

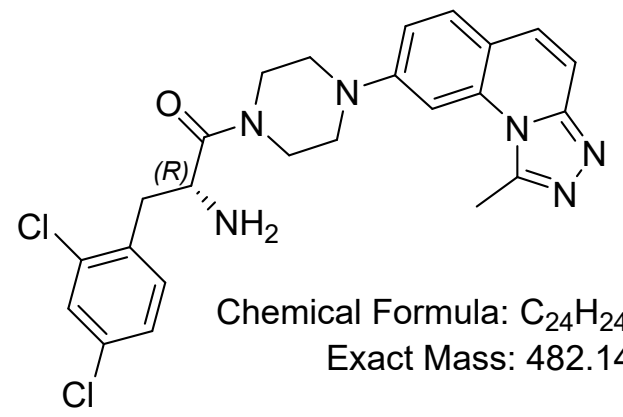

Chemical Formula: $C_{24}H_{24}Cl_2N_6O$
Exact Mass: 482.14

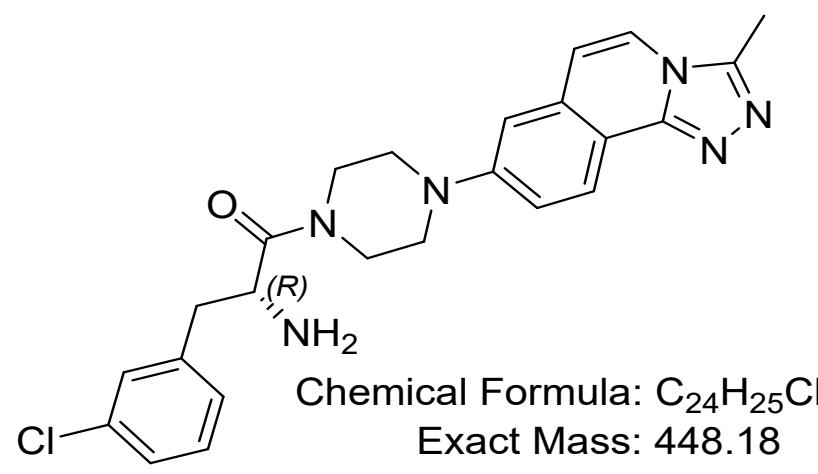

Chemical Formula: $C_{24}H_{25}ClN_6O$
Exact Mass: 448.18

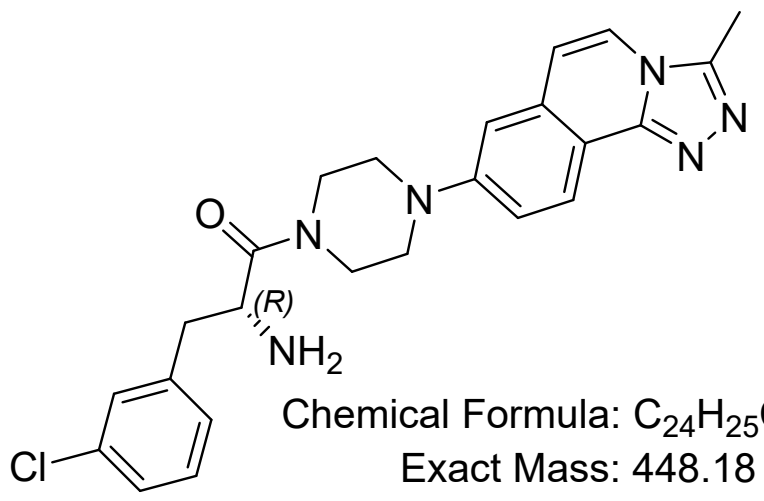

Chemical Formula: $C_{24}H_{25}ClN_6O$
Exact Mass: 448.18

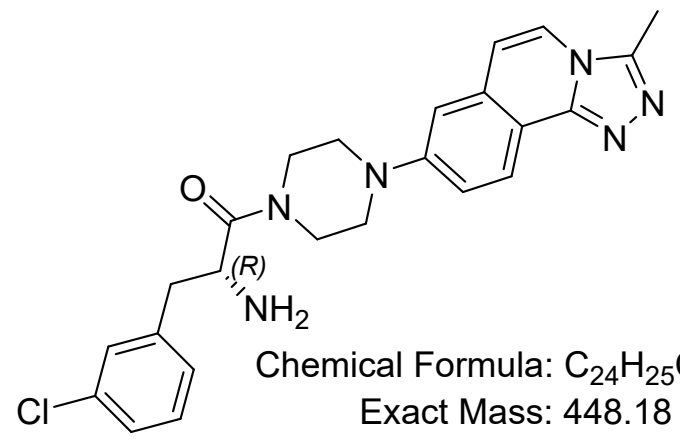

Chemical Formula: $C_{24}H_{25}ClN_6O$
Exact Mass: 448.18

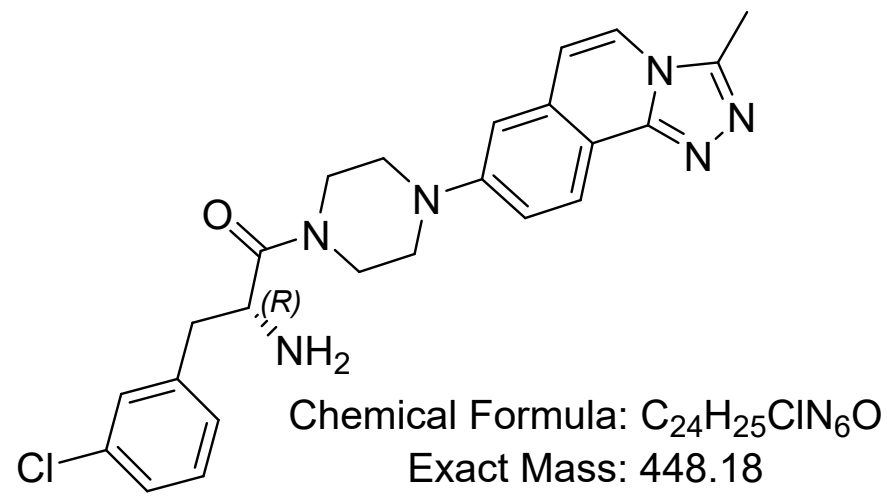

Chemical Formula: $C_{24}H_{25}ClN_6O$
Exact Mass: 448.18

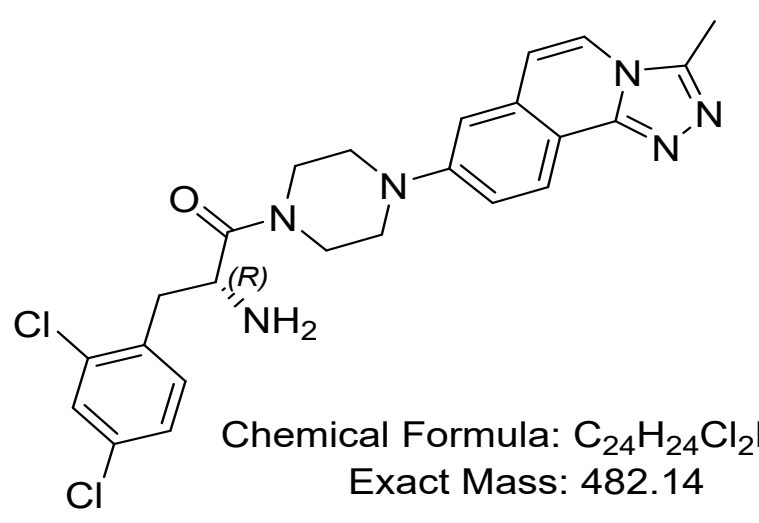

Chemical Formula: $C_{24}H_{24}Cl_2N_6O$
Exact Mass: 482.14

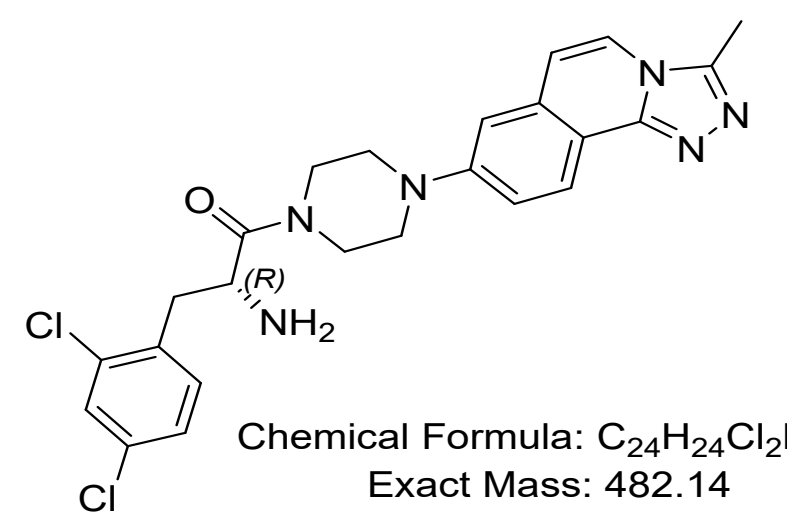

Chemical Formula: $C_{24}H_{24}Cl_2N_6O$
Exact Mass: 482.14

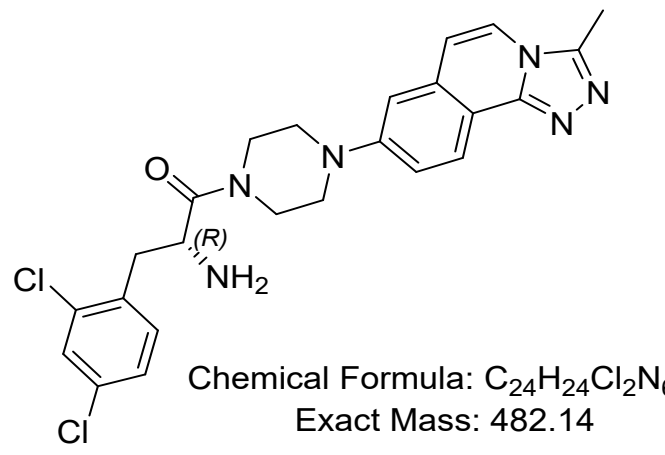

Chemical Formula: $C_{24}H_{24}Cl_2N_6O$
Exact Mass: 482.14

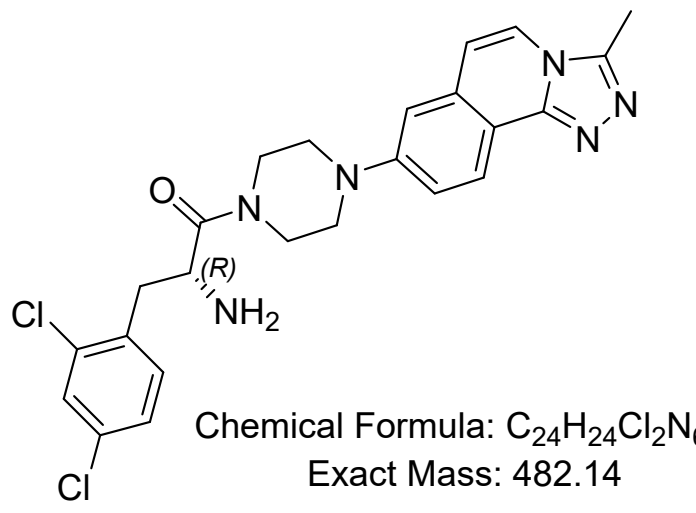

Chemical Formula: $C_{24}H_{24}Cl_2N_6O$
Exact Mass: 482.14

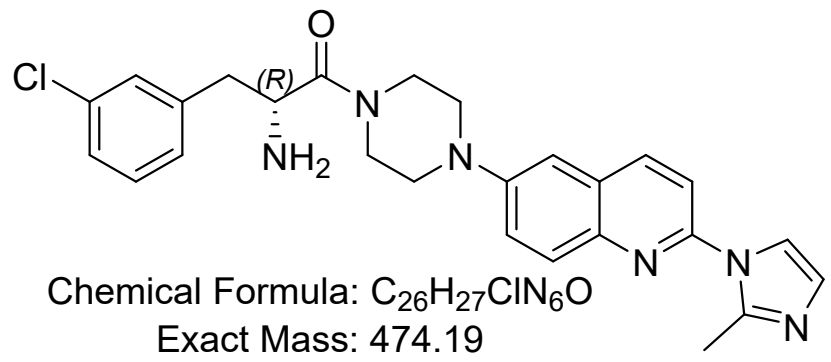

Chemical Formula: $C_{26}H_{27}ClN_6O$
Exact Mass: 474.19

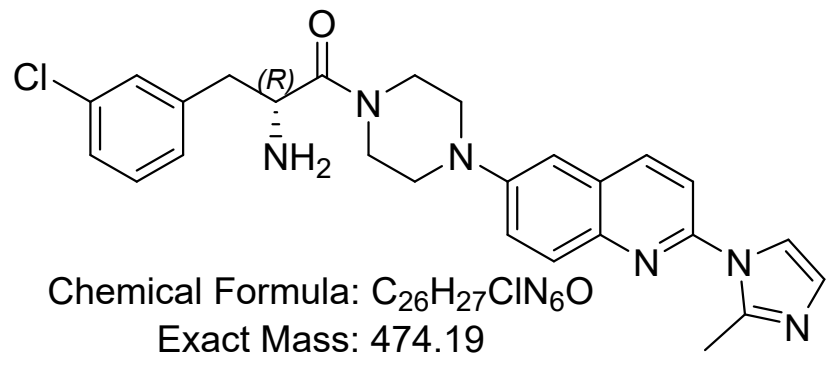

Chemical Formula: $C_{26}H_{27}ClN_6O$
Exact Mass: 474.19

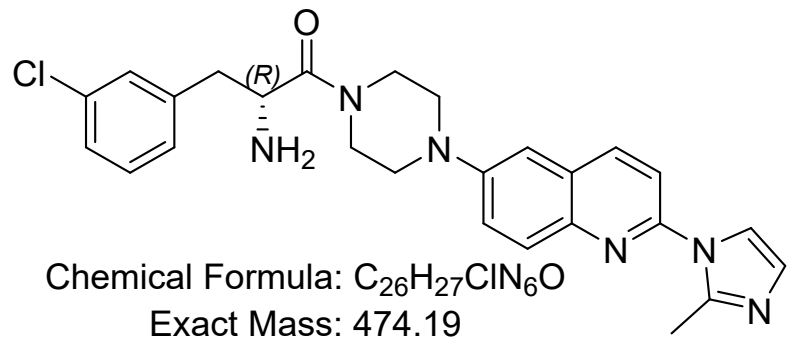

Chemical Formula: $C_{26}H_{27}ClN_6O$
Exact Mass: 474.19

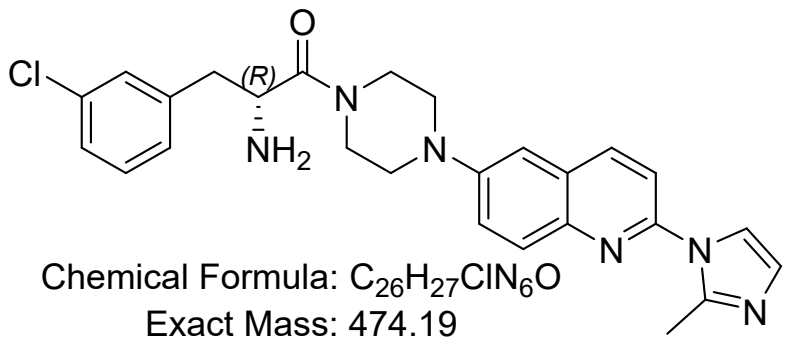

Chemical Formula: $C_{26}H_{27}ClN_6O$
Exact Mass: 474.19

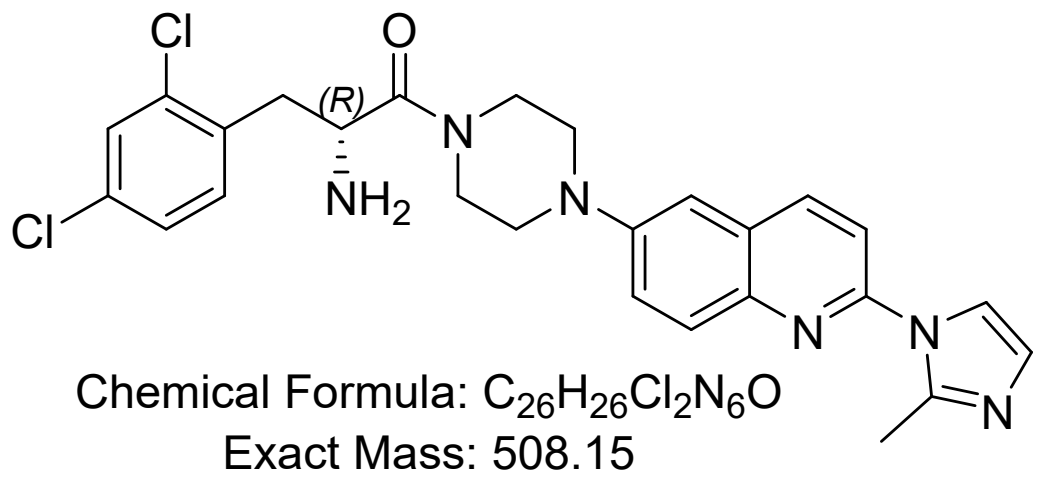

Chemical Formula: $C_{26}H_{26}Cl_2N_6O$
Exact Mass: 508.15

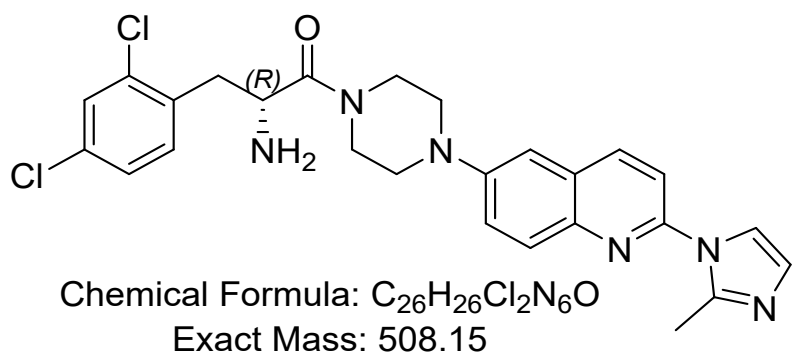

Chemical Formula: $C_{26}H_{26}Cl_2N_6O$
Exact Mass: 508.15

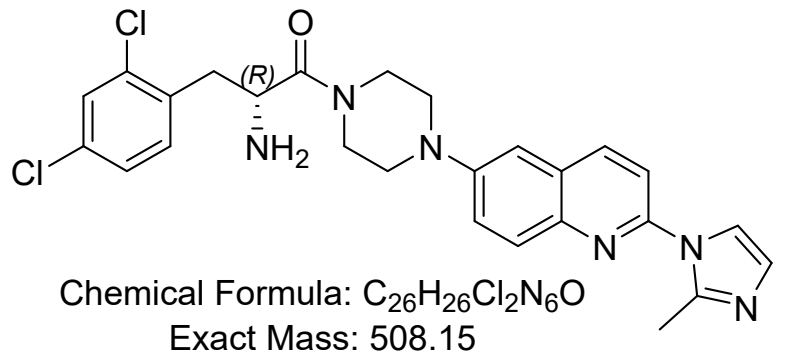

Chemical Formula: $C_{26}H_{26}Cl_2N_6O$
Exact Mass: 508.15

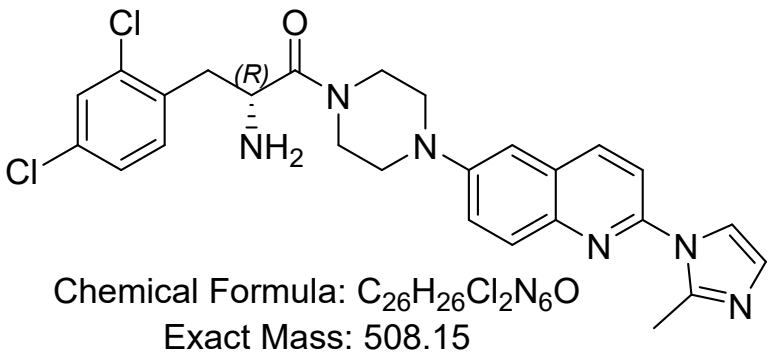

Chemical Formula: $C_{26}H_{26}Cl_2N_6O$
Exact Mass: 508.15

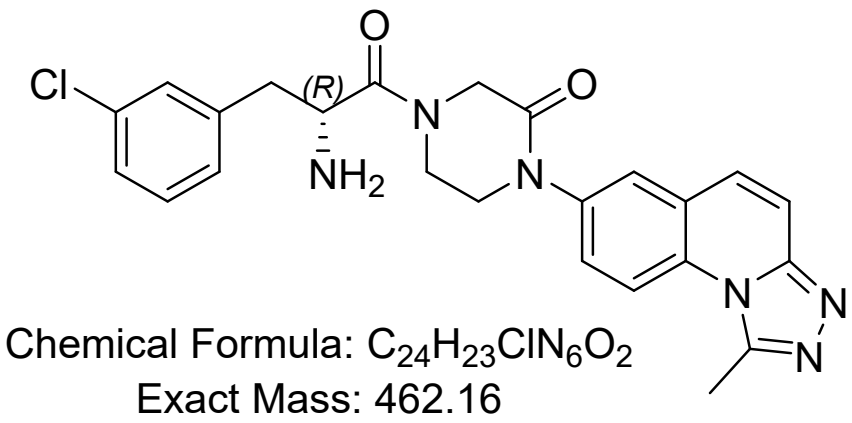

Chemical Formula: $C_{24}H_{23}ClN_6O_2$
Exact Mass: 462.16

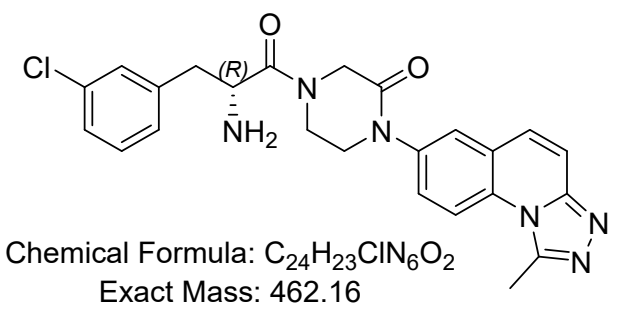

Chemical Formula: $C_{24}H_{23}ClN_6O_2$
Exact Mass: 462.16

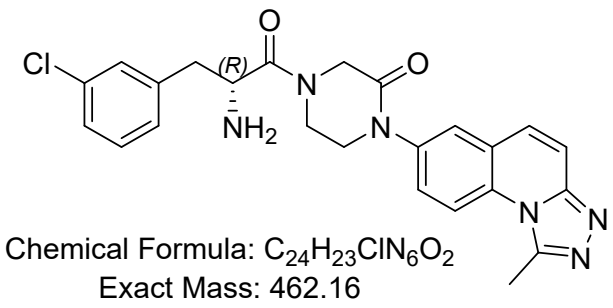

Chemical Formula: $C_{24}H_{23}ClN_6O_2$
Exact Mass: 462.16

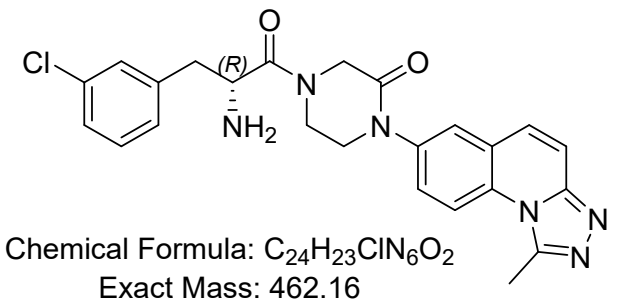

Chemical Formula: $C_{24}H_{23}ClN_6O_2$
Exact Mass: 462.16

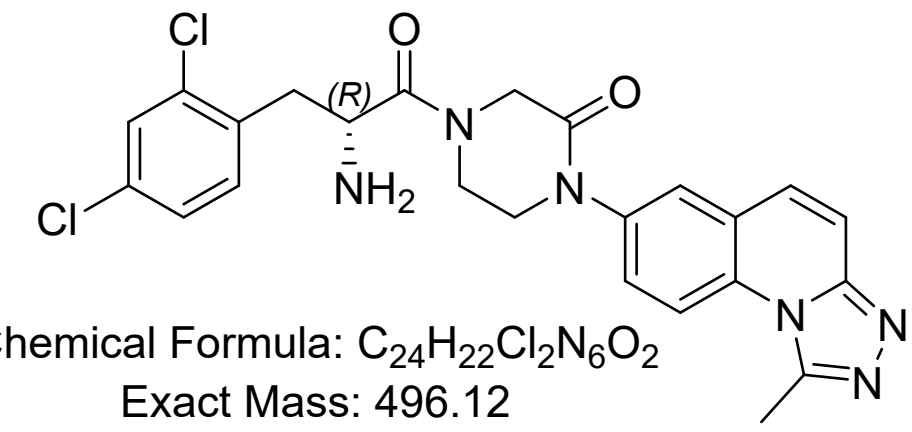

Chemical Formula: $C_{24}H_{22}Cl_2N_6O_2$
Exact Mass: 496.12

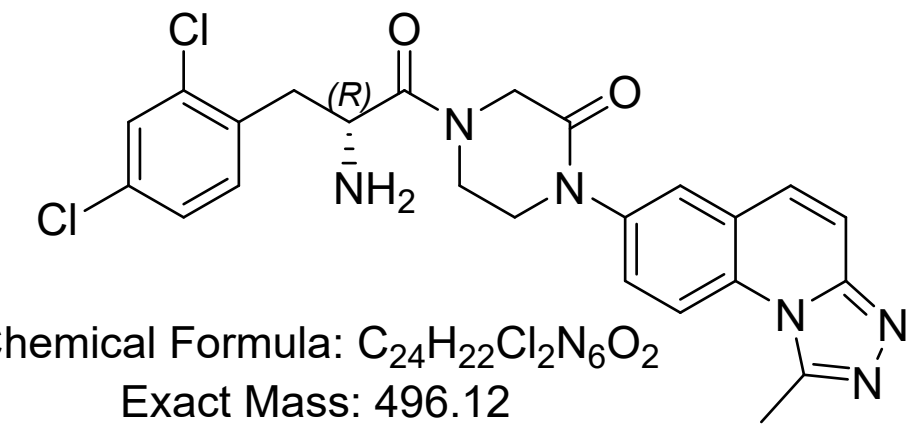

Chemical Formula: $C_{24}H_{22}Cl_2N_6O_2$
Exact Mass: 496.12

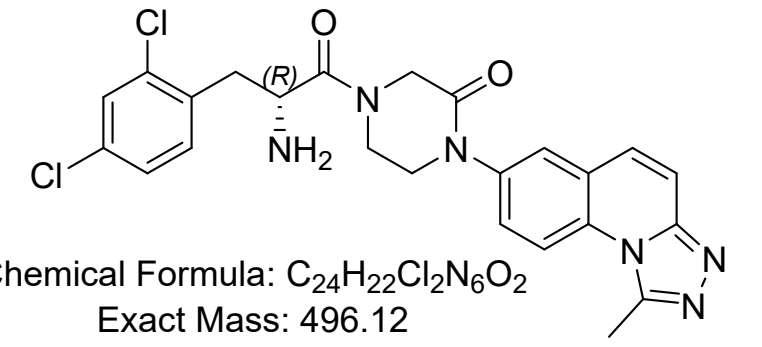

Chemical Formula: $C_{24}H_{22}Cl_2N_6O_2$
Exact Mass: 496.12

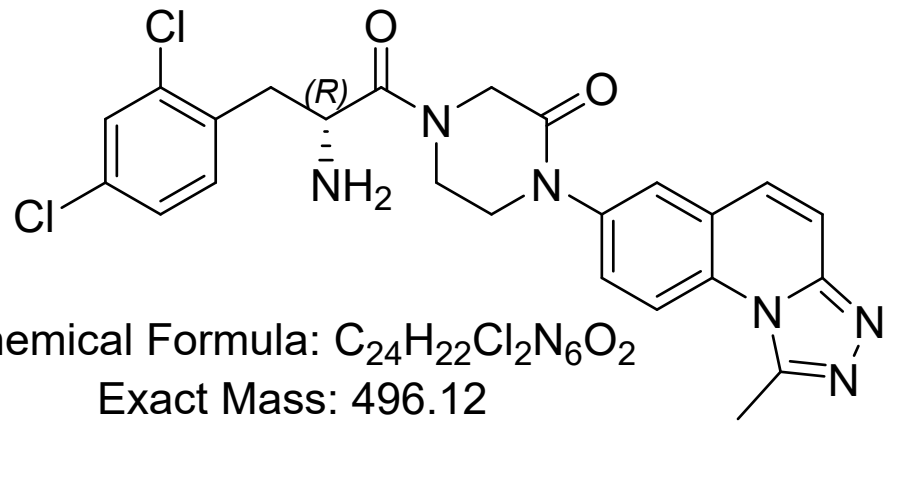

Chemical Formula: $C_{24}H_{22}Cl_2N_6O_2$

Exact Mass: 496.12

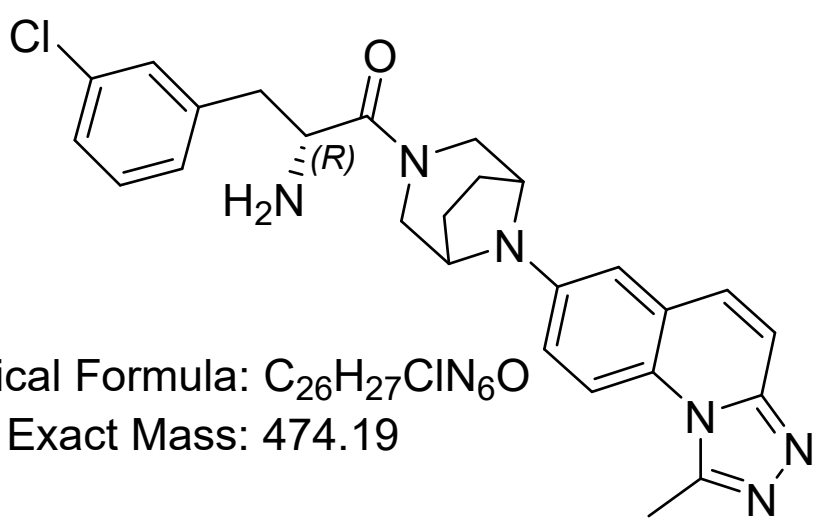

Chemical Formula: $C_{26}H_{27}ClN_6O$
Exact Mass: 474.19

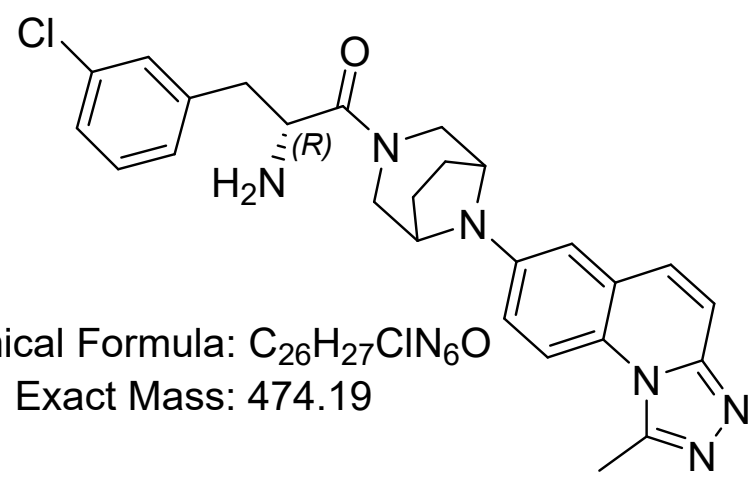

Chemical Formula: $C_{26}H_{27}ClN_6O$
Exact Mass: 474.19

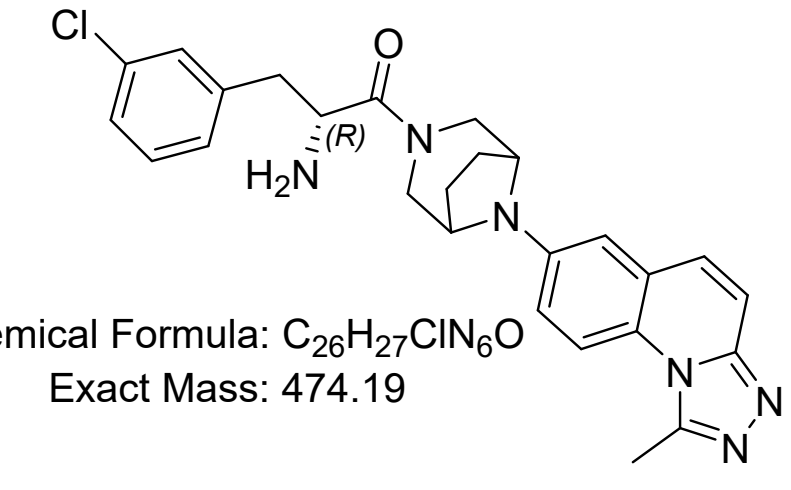

Chemical Formula: $C_{26}H_{27}ClN_6O$
Exact Mass: 474.19

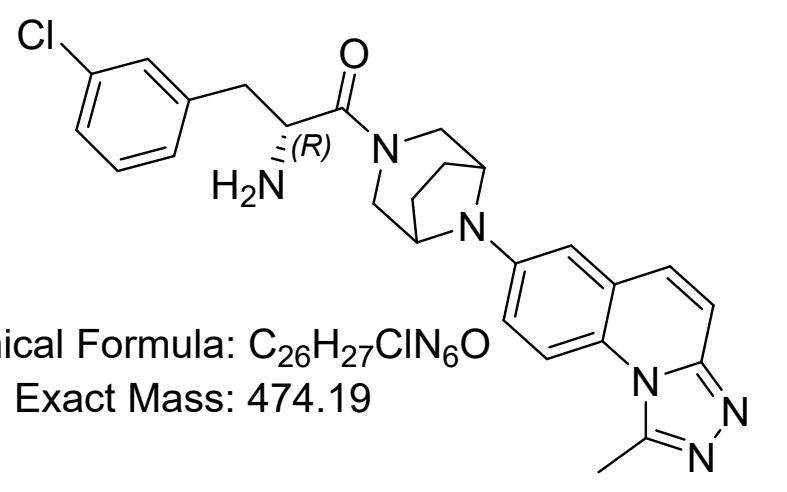

Chemical Formula: $C_{26}H_{27}ClN_6O$
Exact Mass: 474.19

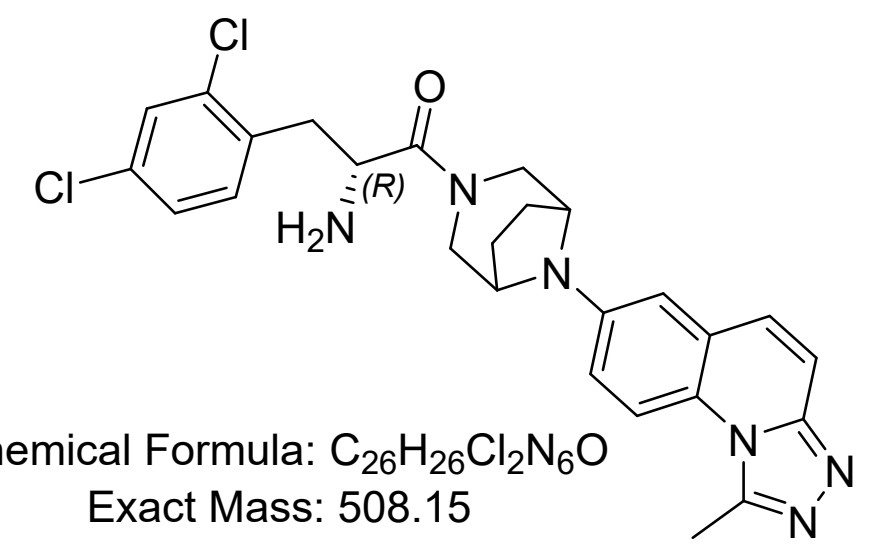

Chemical Formula: $C_{26}H_{26}Cl_2N_6O$
Exact Mass: 508.15

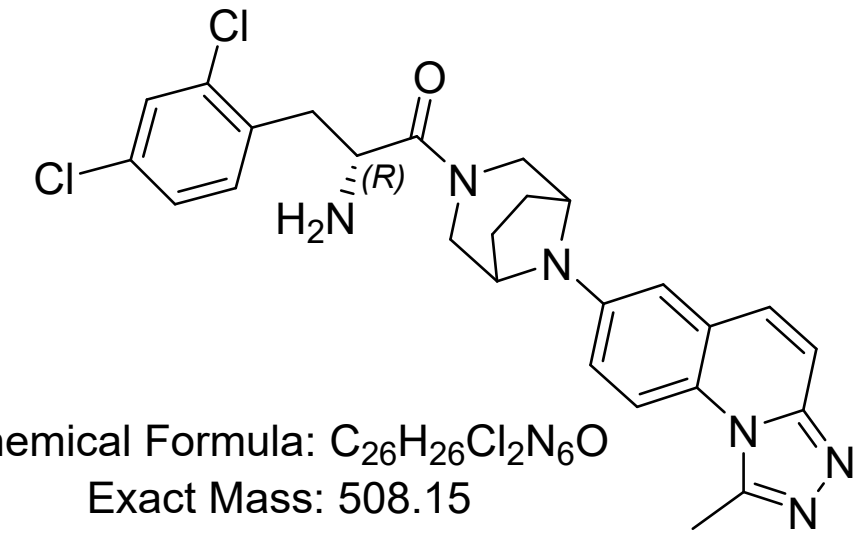

Chemical Formula: $C_{26}H_{26}Cl_2N_6O$
Exact Mass: 508.15

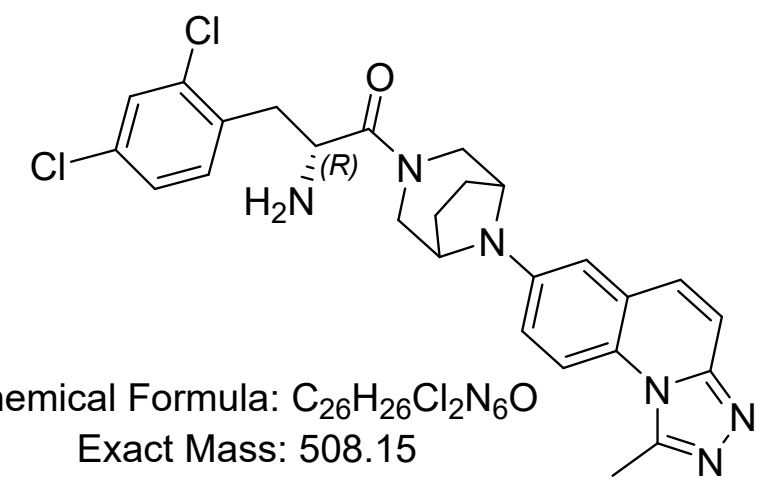

Chemical Formula: $C_{26}H_{26}Cl_2N_6O$
Exact Mass: 508.15

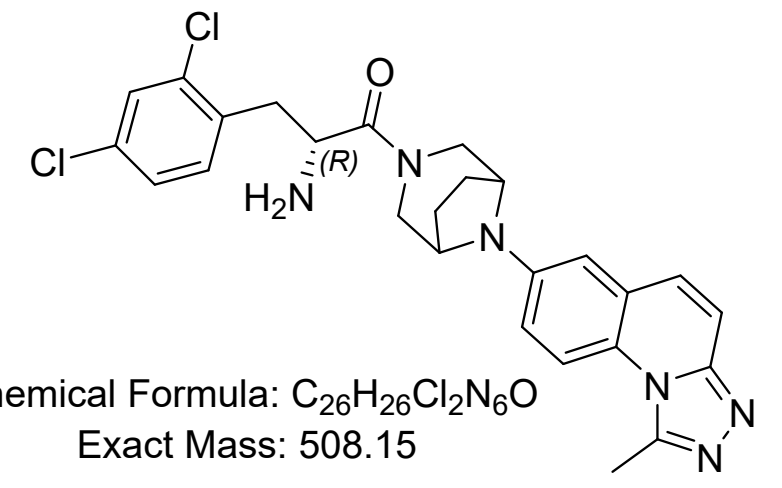

Chemical Formula: $C_{26}H_{26}Cl_2N_6O$
Exact Mass: 508.15

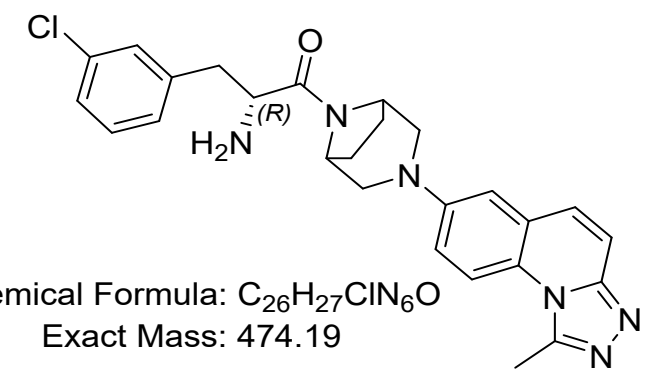

Chemical Formula: $C_{26}H_{27}ClN_6O$
Exact Mass: 474.19

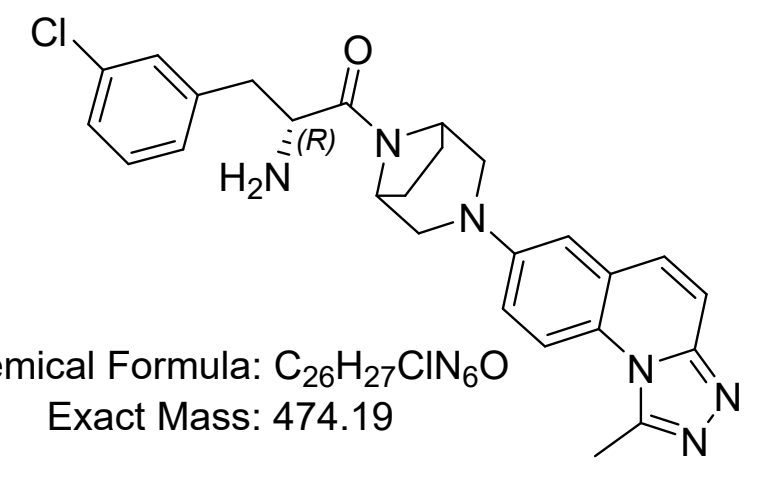

Chemical Formula: $C_{26}H_{27}ClN_6O$
Exact Mass: 474.19

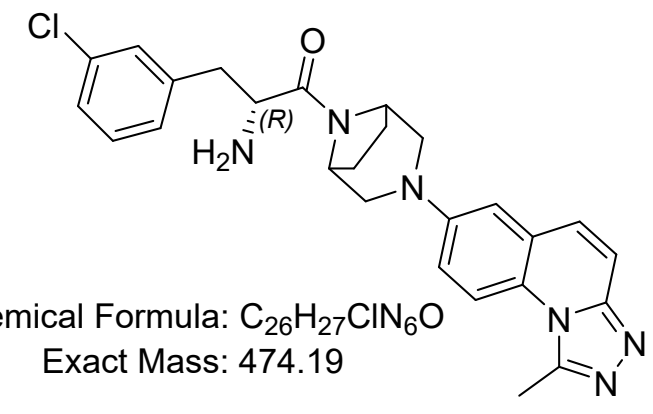

Chemical Formula: $C_{26}H_{27}ClN_6O$
Exact Mass: 474.19

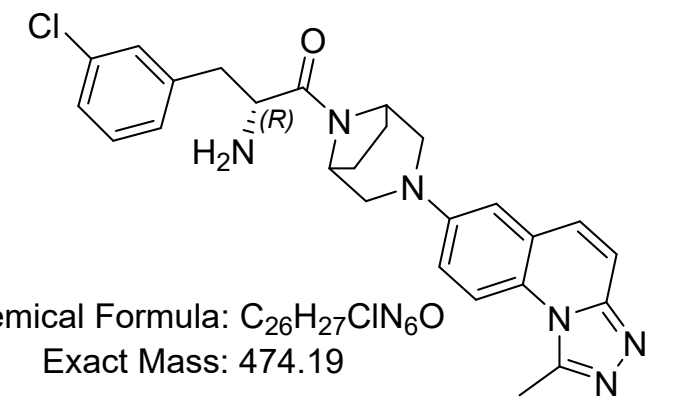

Chemical Formula: $C_{26}H_{27}ClN_6O$
Exact Mass: 474.19

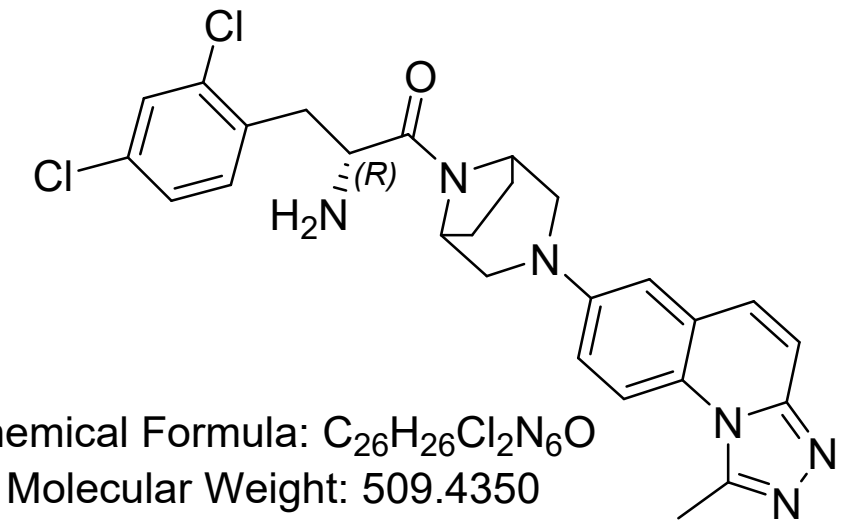

Chemical Formula: $C_{26}H_{26}Cl_2N_6O$
Molecular Weight: 509.4350

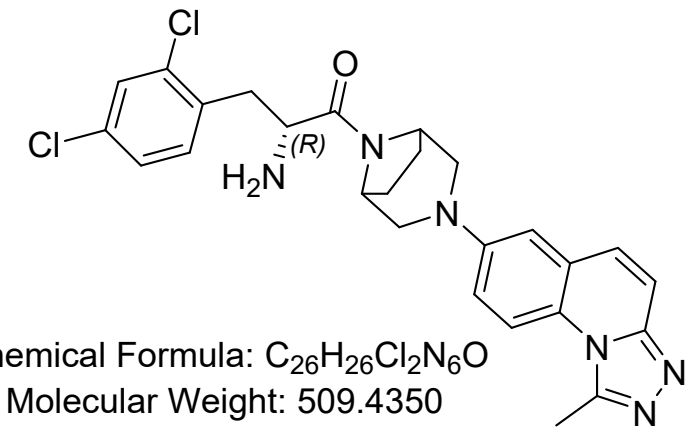

Chemical Formula: $C_{26}H_{26}Cl_2N_6O$
Molecular Weight: 509.4350

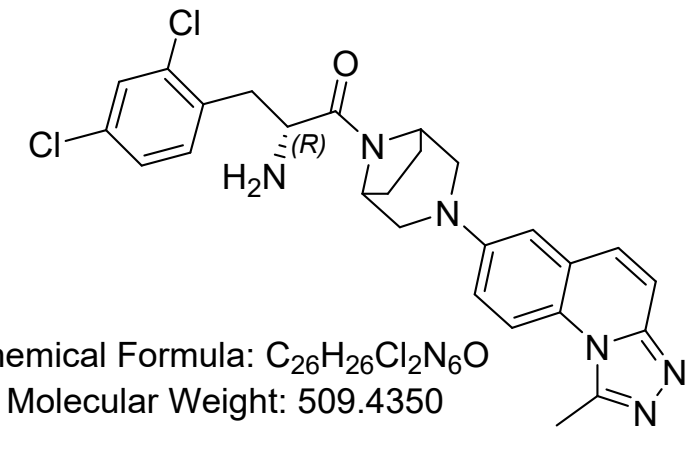

Chemical Formula: $C_{26}H_{26}Cl_2N_6O$
Molecular Weight: 509.4350

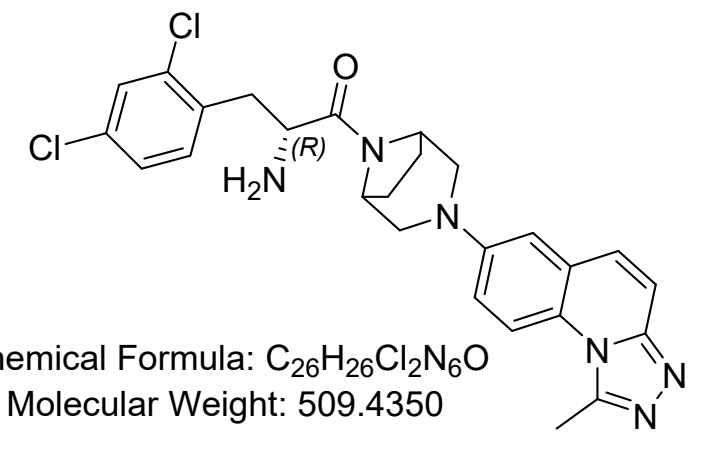

Chemical Formula: $C_{26}H_{26}Cl_2N_6O$
Molecular Weight: 509.4350

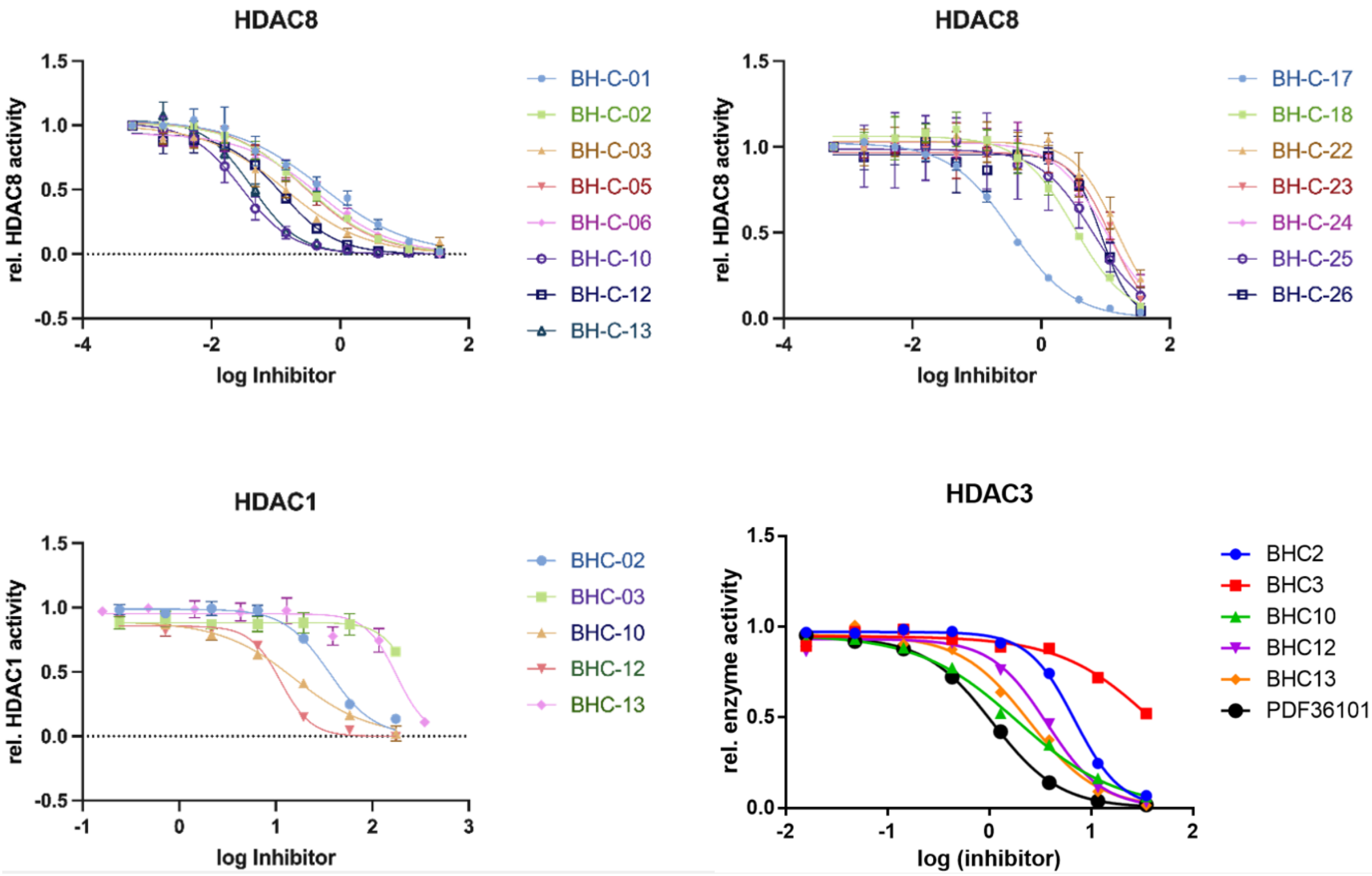

Figure S105: Dose-response curves against indicated HDAC-isoenzymes.

A)

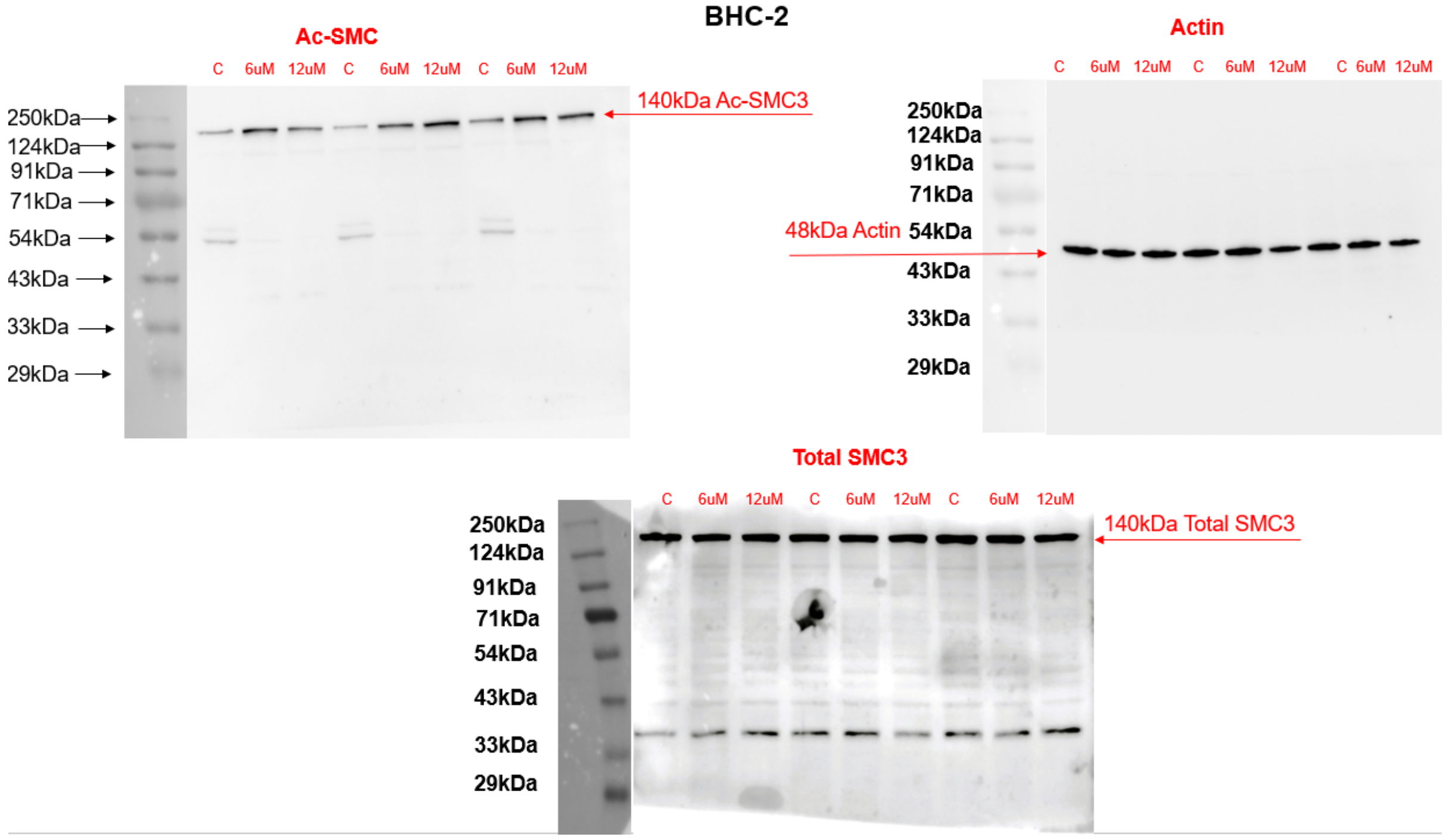
BHC-2
Ac-SMC
C 6uM 12uM C 6uM 12uM C 6uM 12uM
140kDa Ac-SMC3
250kDa
124kDa
91kDa
71kDa
54kDa
43kDa
33kDa
29kDa
Actin
C 6uM 12uM C 6uM 12uM C 6uM 12uM
48kDa Actin
Total SMC3
C 6uM 12uM C 6uM 12uM C 6uM 12uM
140kDa Total SMC3

B)

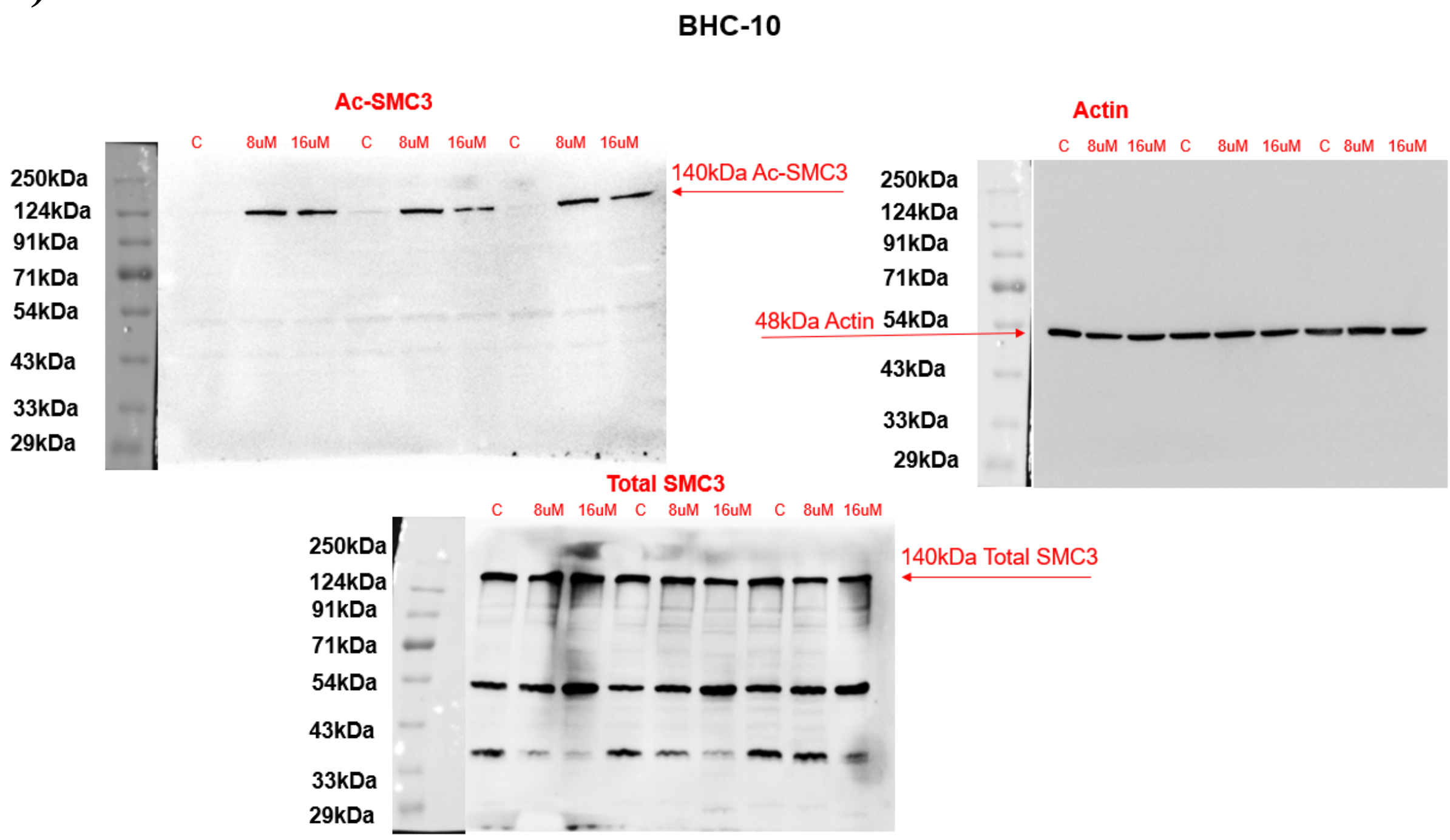
BHC-10
Ac-SMC3
C 8uM 16uM C 8uM 16uM C 8uM 16uM
140kDa Ac-SMC3
250kDa
124kDa
91kDa
71kDa
54kDa
43kDa
33kDa
29kDa
Actin
C 8uM 16uM C 8uM 16uM C 8uM 16uM
48kDa Actin
Total SMC3
C 8uM 16uM C 8uM 16uM C 8uM 16uM
140kDa Total SMC3

C)

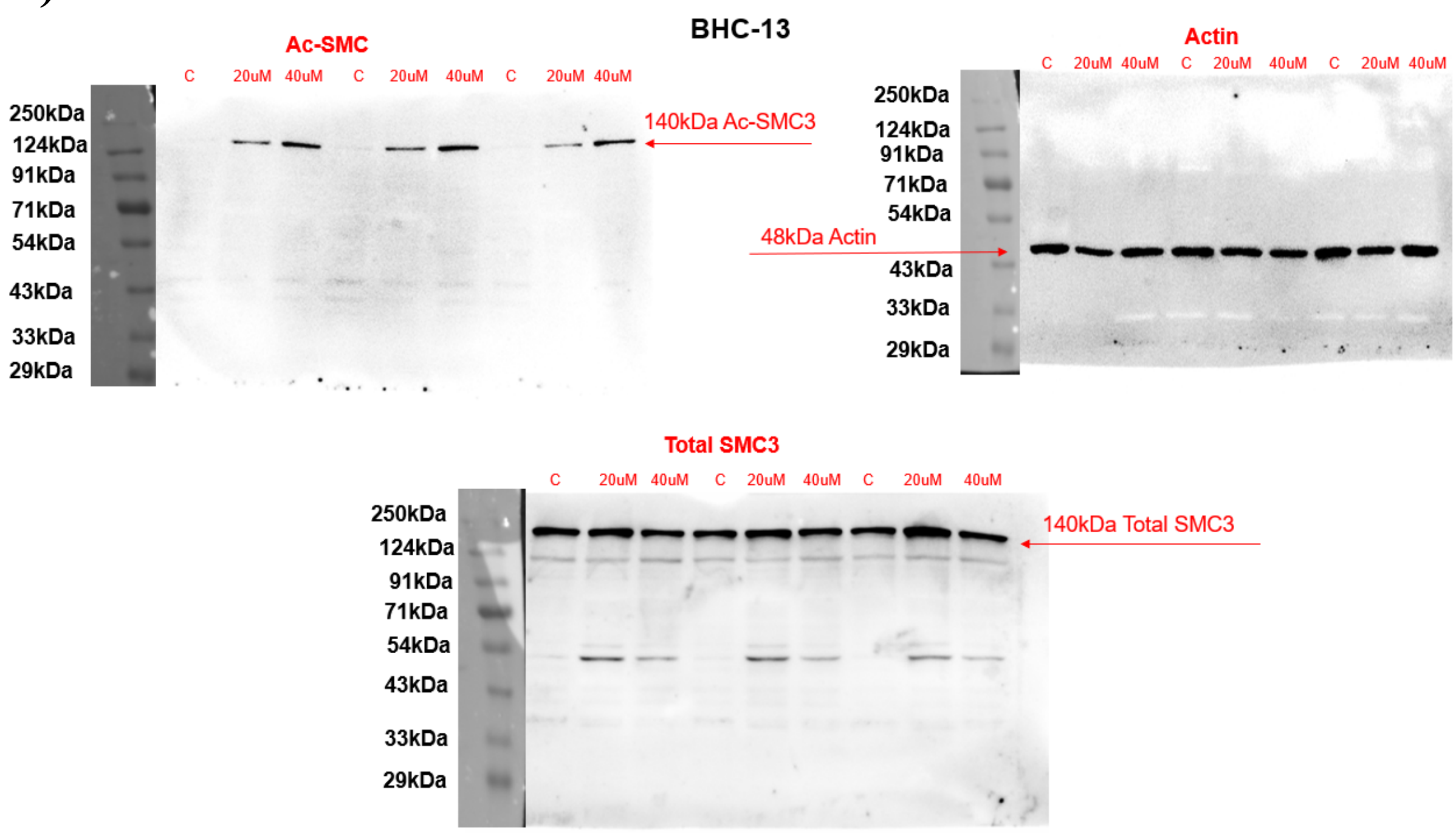

Figure S106: Uncropped images of Western Blot analysis showing the effects of A) BHC2, B) BHC10, and C) BHC13 on SMC3 acetylation and total SMC3 levels. (see Figure 9)
